# A Critical Review of Organic Ultraviolet Filter Exposure, Hazard, and Risk to Corals

**DOI:** 10.1002/etc.4948

**Published:** 2021-02-02

**Authors:** Carys L. Mitchelmore, Emily E. Burns, Annaleise Conway, Andrew Heyes, Iain A. Davies

**Affiliations:** ^1^ University of Maryland Center for Environmental Science Chesapeake Biological Laboratory, Solomons Maryland USA; ^2^ Personal Care Products Council Washington DC USA

**Keywords:** UV filters, Corals, Sunscreen, Environmental chemistry, Hazard/risk assessment, Personal care products

## Abstract

There has been a rapid increase in public, political, and scientific interest regarding the impact of organic ultraviolet (UV) filters to coral reefs. Such filters are found in sunscreens and other consumer products and enter the aquatic environment via direct (i.e., recreational activities, effluents) or indirect (i.e., land runoff) pathways. This review summarizes the current state of the science regarding the concentration of organic UV filters in seawater and sediment near coral reef ecosystems and in coral tissues, toxicological data from early and adult life stages of coral species, and preliminary environmental risk characterizations. Up to 14 different organic UV filters in seawater near coral reefs have been reported across 12 studies, with the majority of concentrations in the nanograms per liter range. Nine papers report toxicological findings from no response to a variety of biological effects occurring in the micrograms per liter to milligrams per liter range, in part given the wide variations in experimental design and coral species and/or life stage used. This review presents key findings; scientific data gaps; flaws in assumptions, practice, and inference; and a number of recommendations for future studies to assess the environmental risk of organic UV filters to coral reef ecosystems. *Environ Toxicol Chem* 2021;40:967–988. © 2021 The Authors. *Environmental Toxicology and Chemistry* published by Wiley Periodicals LLC on behalf of SETAC.

## Graphical abstract

Limited and variable ultraviolet (UV) filter monitoring data in coral reef environments in conjunction with limited toxicity information with significant methodological issues have led to limited and unreliable risk assessment of UV filters to corals. It is our recommendation that increased monitoring and toxicity testing along with harmonized guidelines for coral toxicity testing and risk assessment calculation would help alleviate these issues.

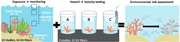

## INTRODUCTION

Organic ultraviolet (UV) filters are used in a diverse array of consumer products to inhibit the infiltration of UV light to prevent sunburns or photodegradation. Examples include sun protection products (e.g., sunscreens), personal care products, plastics, paints, and textiles (Fent et al. 2010; Ramos et al. 2015). Recently, growing scientific, public, and regulatory concern over the presence of organic UV filters, primarily those used in sun protection products, in the environment has emerged (Kim and Choi 2014; Wood 2018; Schneider and Lim 2019). The presence of organic UV filters in the marine environment, primarily released during recreational activities (e.g., swimming), has been highlighted because they are suspected of adversely impacting ecologically important coral communities (Raffa et al. 2019).

Coral reefs are highly productive and economically vital ecosystems, providing an array of ecosystem services and biodiversity (Moberg and Folke 1999; Woodhead et al. 2019). In recent years, coral reef health globally has significantly declined as a result of climate change impacts (sea level rise, ocean acidification), and repeated bleaching events from sustained elevated temperature events have occurred (Hoegh‐Guldberg et al. 2017; Hughes et al. 2018). Meanwhile, local‐scale stressors including municipal and industrial wastewater effluents, overfishing, recreational activities, and overland runoff (urban and agricultural inputs) have also been shown to directly contribute to coral decline and/or reduce the resilience of corals to global stressors (Owen et al. 2005; Negri and Hoogenboom 2011; Spalding and Brown 2015; Duprey et al. 2016). In particular, heavy metals, nutrients, and various organic chemicals can adversely impact corals at potentially environmentally relevant levels (e.g., van Dam et al. 2011; Forbes et al. 2016; Kroon et al. 2020), particularly in densely populated areas or those that experience significant tourism, especially when combined with sheltered beach environments (Wood 2018).

Toxicological effects resulting from coral exposure to organic UV filters is an emerging hypothesis first proposed by Danovaro et al. (2008) and subsequently explored by Downs et al. (2016) and McCoshum et al. (2016). In response to these findings, legislators in the United States have invoked the precautionary principle to ban the use of 2 organic UV filters in beach sunscreen products, namely, oxybenzone (benzophenone‐3 [BP‐3]) and octinoxate (ethylhexyl methoxycinnamate [EHMC]; Sirois 2019). These bans include Hawaii (SB 2571; State of Hawaii Senate 2018), the US Virgin Islands (Bill 33‐0043; US Virgin Islands 2019), and a locally proposed ban in Key West, Florida (Ordinance File 18‐3253; Key West City Commission 2019). Palau (Remengesau 2018) and Bonaire (Ministries of The Netherlands 2020) have enacted similar sunscreen ingredient bans.

Since the initial 3 organic UV filter coral toxicity papers were published, 6 additional investigations have increased the amount of data available and expanded the number of organic UV filters studied (Fel et al. 2019; He et al. 2019a, 2019b; Stien et al. 2019, 2020; Wijgerde et al. 2020). Two studies have reported on the concentrations of UV filters in coral tissues (Tsui et al. 2017; Mitchelmore et al. 2019). In addition, 12 exposure studies (i.e., chemical monitoring) that collected seawater samples near coral reefs have been reported (Goksøyr et al. 2009; Tashiro and Kameda 2013; Bargar et al. 2015; Downs et al. 2016; Kung et al. 2018; Schaap and Slijkerman 2018; Mitchelmore et al. 2019; Tsui et al. 2019), with a handful of them also conducting preliminary coral risk assessments using the existing empirical data (Tsui et al. 2014, 2017; He et al. 2019a, 2019b). A few studies have also reported concentrations of organic UV filters in sediment near coral reefs (Tsui et al. 2015, 2017; Apel et al. 2018; Mitchelmore et al. 2019).

In this article, we critically review the exposure, hazard, and risk that organic UV filters pose to coral reefs. Major findings are summarized along with recommendations for future research to enhance our understanding of the sources, exposure, fate, and toxicity of organic UV filters on coral environments. It is our hope that the results will help focus future research efforts toward critical knowledge gaps and provide decision‐makers with a state‐of‐the‐science summary to aid in the protection of coral reefs.

## METHODS

A review was conducted of papers published up to the end of June 2020, as described in Supplemental Data, Text S1. Papers that reported toxicological effects on coral (*n* = 9), UV filter exposure in seawater (*n* = 12) or sediment (*n* = 4) near reefs, or UV filter concentrations within coral tissues (*n* = 2) or conducted coral‐specific risk assessments (*n* = 5) were included. To assess trends in UV filter exposure, data were summarized in box plots containing the range and median of concentrations reported per UV filter, per study, as detailed in Supplemental Data, Text S2. To summarize toxicity data, lowest‐observable‐effect concentrations (LOECs) were converted to no‐observable‐effect concentrations (NOECs) for consistency and to be more suitable for risk assessment according to guidance provided by the European Chemicals Agency (2008), as detailed in Supplemental Data, Text S3. Median effective concentrations (EC50s) and median lethal concentrations (LC50s) were not converted because these endpoints are suitable for risk assessment. A cumulative endpoint ecotoxicity distribution was created to help visualize the variability in effect concentrations based on UV filter and endpoint studied. Finally, risk assessments were summarized in a single figure by plotting the risk quotient reported for each compound assessed, as described in Supplemental Data, Text S4.

## SOURCES AND OCCURRENCE OF UV FILTERS IN THE MARINE ENVIRONMENT

**Figure 1:**
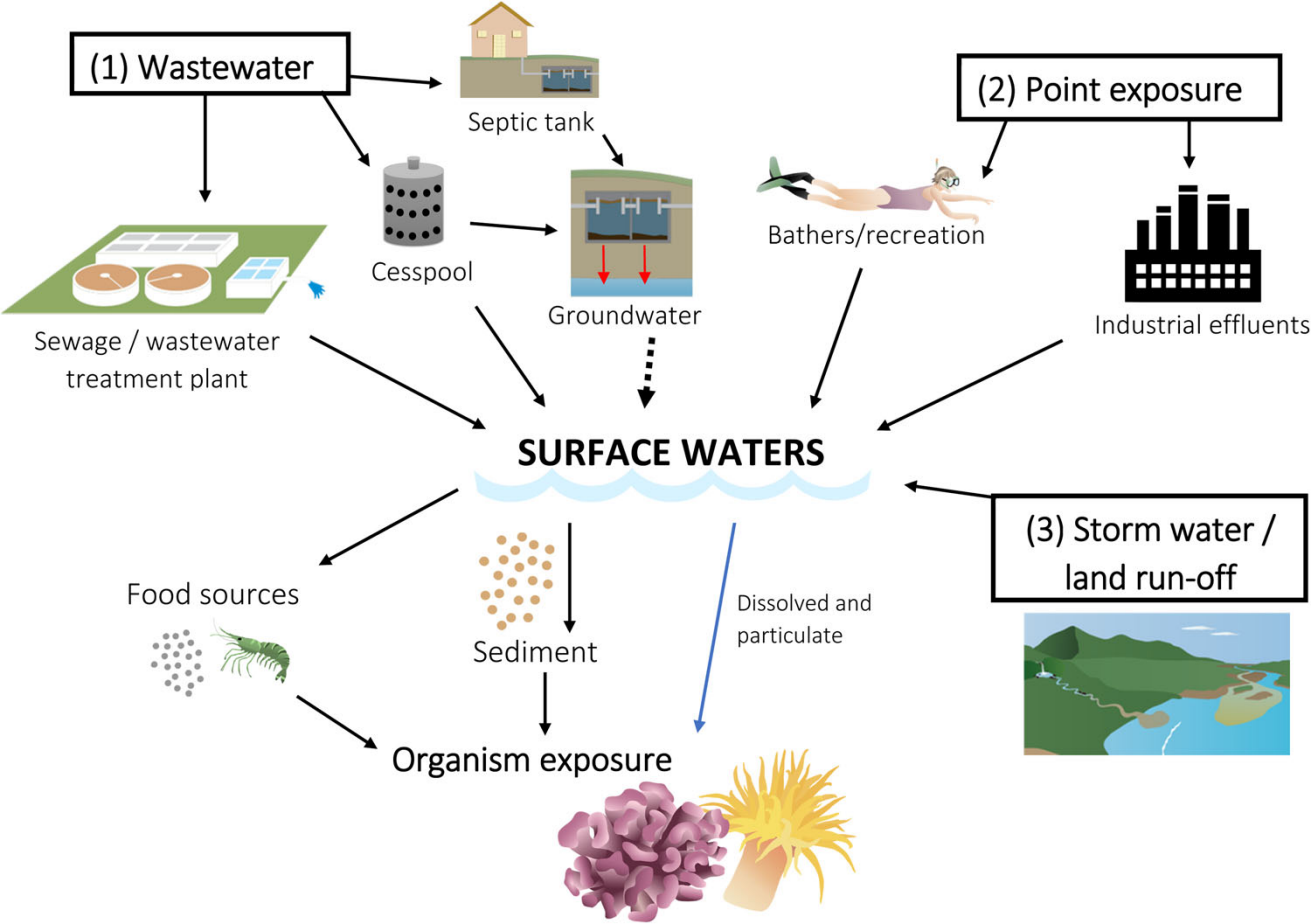
Potential sources and routes of entry of organic ultraviolet filters into the aquatic environment. Images are from the Integration and Application Network, University of Maryland Center for Environmental Science (ian.umces.edu/imagelibrary/), with specific author credits being Caroline Wicks (wastewater treatment plant), Jane Thomas (septic), Joanna Woerner (snorkler and coral), Jane Hawkey (factory, sediment, and shrimp), and Tracey Saxby (runoff and coral).

The pathways of UV filter environmental exposure are varied and source‐dependent. In this review, we focus on UV filters used in sun care products (i.e., sunscreens), which aligns with the findings from a recent monitoring and modeling study (Labille et al. 2020), although efforts to verify this assumption should be undertaken. However, it is important to note that UV filters are used in a wide range of products and that their UV filter content, their leaching potential, and the extent of their contribution to environmental concentrations are virtually unknown, complicating the environmental source apportionment of these compounds from sunscreens. What we can say is that given the diversity of organic UV filter sources, there are multiple point and diffuse sources of UV filters to the aquatic environment (e.g., see Giokas et al. 2007) including from recreational use (i.e., swimmers), wastewater‐treatment plant effluents, industrial effluents, and terrestrial runoff (Figure 1).

In sun protection products, UV filters are usually added as a mixture at various concentrations to protect the skin from the negative consequences of UVA (315–400 nm) and UVB (280–315 nm) light exposure including sunburn, premature aging, and skin cancer (Chisvert and Salvador 2007; Giokas et al. 2007). Organic UV filters protect skin by absorbing UV light, and those available for formulation in sun care products vary regionally along with their percentage permitted. For example, US cosmetic products can contain up to 6% oxybenzone (BP‐3) and 7.5% octinoxate (EHMC), whereas their inclusion levels are higher in the European Union (i.e., 10%, for BP‐3 and EHMC; Table 1). However, ingredients not used in a certain country may be brought in and used by visitors and tourists from other countries (Schaap and Slijkerman 2018). Many of the UV filters in Table 1 have yet to be investigated in terms of coral toxicity (48%) or appear in an environmental exposure study (45%). For those that have been studied, different names and acronyms have been used to identify them. For example, the acronym for “octisalate,” also “ethylhexyl salicylate,” has been cited as “OS” (Mitchelmore et al. 2019) and “EHS” (ethylhexyl salicylate; Danovaro et al. 2008; Tsui et al. 2014). We propose identifying all UV filters by their International Nomenclature of Cosmetic Ingredients (INCI) name and using the proposed acronyms in subsequent research efforts (Table 1).

**Table 1:** Detailed summary of the common types of organic ultraviolet (UV) filters used in sunscreens and cosmetics globally^a^

INCI name (INN/USAN/ANN)	Standardized abbreviation	Maximum product inclusion levels (%)				Physicochemical properties^b^		Abbreviations reported in other studies
		USA	EU	AUS	JPN	Log *K*_OW_	Solubility (mg/L)	
Butyl methoxydibenzoylmethane (avobenzone)	AVO	3	5	5	10	6.1	0.027	BMDMB^f^; BMDM^i^
Menthyl anthranilate (meradimate)	MA	5	NA	5	NA	6.28^c^	0.074^c^	
Disodium phenyl dibenzimidazole tetrasulfonate (bisdisulizole disodium)	BPDT	NA	10	10	10	–2	2000	
Diethylamino hydroxybenzoyl hexyl benzoate (none listed)	DHHB	NA	10	10	10	6.2	0.016	
Ethylhexyl dimethyl PABA (padimate O)	EDP	8	8	8	10	6.2	0.11	ODPABA^e^, ^g^; OD‐PABA^g^
Cinoxate (cinoxate)	CIN	3	NA	6	5	2.65^c^	127.4^c^	CIN^f^
Homosalate (homosalate)	HMS	15	10	15	10	6.34	0.5	HMS^f^ ^–h^
Ethylhexyl methoxycinnamate (octinoxate)	EHMC	7.5	10	10	10	6.1	0.051	EHMC^g^, ^h^; OCT^g^; OMC^i^, ^j^
Ethylhexyl salicylate (octisalate)	EHS	5	5	5	10	6.36	0.5	OS^f^; EHS^g^
Phenylbenzimidazole sulfonic acid (ensulizole)	PSA	4	8^i^	4	3	–1.42	109	ESZ^f^
Triethanolamine salt of salicylate (trolamine salicylate)	TEAS	12	NA	12	NA	2.06^d^	—	TEAS^f^
Benzylidene camphor sulfonic acid (none listed)	BCSA	NA	8	6	6	2.22^c^	119.5^c^	
Terephthalylidene dicamphor sulfonic acid (ecamsule)	TDSA	NA	10	10	10	–1.8^e^	6 × 10^5^ e	
Para‐aminobenzoic acid (aminobenzoic acid)	PABA	15	NA	15	4	0.68	6100	
PEG‐25 PABA (none listed)	PEG‐25	NA	10	10	10	—	—	
4‐Methylbenzylidene camphor (enzacamene)	4‐MBC	NA	4	4	NA	5.1	1.08	4MBC^f^; 4‐MBC^g^, ^h^, ^i^
Camphor benzalkonium methosulfate (none listed)	CBM	NA	6	6	6	—	—	
Ethylhexyl triazone (octyl triazone)	ET	NA	5	5	3	7^e^	0.006^e^	
Polysilicone‐15 (polysilicone‐15)	PS‐15	NA	10	10	10	—	0.1	
Polyacrylamidomethyl benzylidene camphor (none listed)	PBC	NA	6	6	6	—	—	
Isoamyl p‐methoxycinnamate (amiloxate)	IPM	NA	10	10	NA	4.78	0.8	IAMC^h^
Octocrylene (octocrilene)	OC	10	10	10	10	6.1	0.04	OC^f^, ^h^; OCT^j^
Bis‐ethylhexyloxyphenol methoxyphenyl triazine (bemotrizinol)	BEMT	NA	10	10	3	—	—	
Phenylene bis‐diphenyltriazine (none listed)	PBD	NA	5	NA	NA	—	—	
Tris‐biphenyl triazine (none listed)	TBPT	NA	10	10	10	5.6	0.00003	
Drometrizole trisiloxane (drometrizole trisiloxane)	DT	NA	15	15	NA	>6^e^	<0.04^e^	
Diethylhexyl butamido triazone (iscotrizinol)	DBT	NA	10	NA	5	4.1–5.9	0.005	
Benzophenone‐3 (oxybenzone)	BP‐3	6	10	10	5	3.45	6	BP‐3^f^, ^g^, ^h^; BZ^j^
Benzophenone‐4 (sulisobenzone)	BP‐4	10	5	10	10	0.52	300 000	SSB^f^; BP‐4^h^
Benzophenone‐8 (dioxybenzone)	BP‐8	3	NA	3	NA	2.33	0.013	BP8^g^; BP‐8^h^
Methylene bis‐benzotriazolyl tetramethylbutylphenol (bisoctrizole)	MBBT	NA	10	10	10	4.2	0.007	

^a^ Organic UV filters are listed in terms of their International Nomenclature of Cosmetic Ingredients, alternative names, and acronym; select physicochemical properties and their maximal product inclusion levels in the countries they are approved are also reported, and organic UV filters studied thus far are identified by the abbreviation used by the respective authors.

^b^ Experimental physicochemical properties obtained from publicly available Registration, Evaluation, Authorization and Restriction of Chemicals technical registration dossiers maintained by the European Chemicals Agency unless otherwise noted (European Chemicals Agency 2020).

^c^ Physicohemical property estimated using EPISuite Software (US Environmental Protection Agency 2020a).

^d^ Predicted physicochemical property reported by the US Environmental Protection Agency Chemistry Dashboard (US Environmental Protection Agency 2020b).

^e^ Reported by Fel et al. (2019; names used in paper are uvinul T150 [for ET], mexoryl XL [for DT], mexoryl SX [for TSDA]).

^f^ Reported by Mitchelmore et al. (2019).

^g^ Reported by Bargar et al. (2015).

^h^ Reported by Tsui et al. (2014).

^i^ Reported by Horricks et al. (2019).

^j^ Reported by Danovaro et al. (2008).

Benzophenone‐1 is not marketed as a sunscreen agent and therefore not included in this table. It is included in subsequent analysis because it is a known metabolite of BP‐3 (He et al. 2019b).

AUS = Australia; EU = European Union; INCI = International Nomenclature of Cosmetic Ingredients; INN/USAN/ANN = International nonproprietary name/United States adopted name/Australian approved name; JPN = Japan; *K*_OW_ = octanol–water partition coefficient; NA = not available.

Finally, Table 1 and Supplemental Data, Table S1, demonstrate that UV filters are a physiochemically diverse group of chemicals, indicating that once in the environment their fate will be UV filter‐specific. This is evidenced by the large range in log octanol–water partition coefficients (*K*_OW_) and solubility. Solubility estimates (predicted or measured) are variable even for the same UV filter and are reported for freshwater rather than seawater, which is expected to result in lowered UV filter solubility (Xie et al. 1997). Furthermore, the fate of UV filters in the environment is influenced by a number of chemical and physical factors (e.g., salinity), as described in Supplemental Data, Text S5. The differences in physicochemical parameters in a particular environment, in addition to the structural dissimilarity between UV filters (Supplemental Data, Table S1), are likely to affect the relative toxicity of these compounds, limiting the ability to “read across” effect or fate information between data‐rich and data‐poor compounds. Overall, relatively little is known about the fate of UV filters in the environment despite the importance of these fate processes in determining their bioavailability to aquatic organisms, and further studies are recommended.

### UV filter environmental occurrence near coral reefs

We have identified only a handful of exposure studies reporting measured environmental concentrations (MECs) of UV filters in the water column near coral reefs (*n* = 12), in sediment (*n* = 4), and within coral tissue (*n* = 2), which are summarized in Tables 2 and 3 and Supplemental Data, Table S2. The current data set is limited but can still provide initial insight into the environmental occurrence and distribution of UV filters in and around reef systems. By limiting the data set to relevant reef exposure, we aim to characterize the exposure that coral is most likely to experience, which from a risk‐assessment perspective is desirable.

**Table 2:** Concentrations of organic ultraviolet filters reported in sediments (nanograms per gram dry wt) at sites near coral reefs: The median, range, and detection frequency are provided for each compound at each site reported

	Mitchelmore et al. (2019)^a^			Tsui et al. (2015)			Tsui et al. (2017)			Apel et al. (2018)^b^		
Compound	Median	(Range)	DF (%)	Median	(Range)	DF (%)	Median	(Range)	DF (%)	Median	(Range)	DF (%)
BP‐3	0.05	(<LOD–4.3)	79	4.1	(<LOD–39.8)	32	8.1	(4.2–17.8)	100	<LOD		0
EHMC	<LOD	(<LOD–12.7)	37	7.4	(<LOD–447)	85	<LOD		0	<LOQ	(<LOD–0.24)	21
OC	0.64	(<LOD–19.8)	68	5.0	(<LOD–15.6)	19	2.1	(<LOD–3.1)	57	0.83	(<LOD–25)	46
HMS	5.05	(0.08–38.5)	100	<LOD		0				<LOQ	(<LOD–0.94)	32
EDP	<LOD		0	19.5	(<LOD–150)	70	3.4	(<LOD–8)	57	<LOD	(<LOD–0.004)	4
4‐MBC	<LOD	(<LOD–<LOQ)	11	<LOD		0	<LOD		0	<LOD		0
EHS	2.65	(0.16–19.6)	100	<LOD		0				0.16	(<LOD–1.35)	45
AVO	<LOQ	(<LOD–6.9)	53	9.7	(<LOD–64.5)	72						
TEAS	<LOD	(<LOD–<LOQ)	5									
BP‐1				2.1	(<LOD–14.6)	66	<LOD		0			
BP‐8	<LOD		0	10.5	(<LOD–62.2)	81	<LOD		0			

^a^ Mitchelmore et al. (2019) found <LOD for cinoxate, phenylbenzimidazole sulfonic acid, BP‐4, and isoamyl p‐methoxycinnamate (IPM).

^b^ Apel et al. (2018) also found <LOD for IPM.

Sample sites included per study: *n* = 19, Mitchelmore et al. (2019); *n* = 47, Tsui et al. (2015); *n* = 7, Tsui et al. (2017); *n* = 74, Apel et al. (2018). A detailed explanation for how these data were summarized is provided in Supplemental Data, Text S6.

AVO = avobenzone; BP = benzophenone; DF = detection frequency; EDP = ethylhexyl dimethyl para‐aminobenzoic acid; EHMC = ethylhexyl methoxycinnamate; EHS = ethylhexyl salicylate; HMS = homosalate; LOD = limit of detection; LOQ = limit of quantification; 4‐MBC = 4‐methylbenzylidene camphor; OC = octocrylene; TEAS = triethanolamine salt of salicylate.

**Table 3:** Concentrations of organic ultraviolet filters reported in corals (nanograms per gram wet wt or dry wt)^a^

	Mitchelmore et al. (2019)^b^			Tsui et al. (2017)^c^		
Compound	Median	(Range)	DF (%)	Median	(Range)	DF (%)
BP‐3	33.8	(5.8–241)	100	9.9	(5.1–21.4)	100
EHMC	<LOD		0	<LOD		0
OC	48.4	(31.3–262)	100	<LOD	(<LOD–3.7)	43
HMS	341	(189–441)	100			
EDP	<LOD		0	<LOD	(<LOD–4.1)	14
4‐MBC	<LOD	(<LOD–32)	21	<LOD		0
EHS	331	(210–527)	100			
AVO	43.5	(<LOD–170)	63			
BP‐8	<LOD		0	8.3	(3.8–12.3)	100

^a^ The median, range, and detection frequency are provided for each compound at each site reported.

^b^ Mitchelmore et al. (2019) found <LOD for cinoxate, triethanolamine salt of salicylate, phenylbenzimidazole sulfonic acid, and BP‐4. Values are for dry weight.

^c^ Tsui et al. (2017) also found <LOD for BP‐1 and BP‐4. Values are for wet weight.

Sample sites included per study: *n* = 19, Mitchelmore et al. (2019); *n* = 7, Tsui et al. (2017). A detailed explanation of how these data were summarized is provided in Supplemental Data, Text S7.

AVO = avobenzone; BP = benzophenone; DF = detection frequency; EDP = ethylhexyl dimethyl para‐aminobenzoic acid; EHMC = ethylhexyl methoxycinnamate; EHS = ethylhexyl salicylate; HMS = homosalate; LOD = limit of detection; 4‐MBC = 4‐methylbenzylidene camphor; OC = octocrylene.

#### Methods of UV filter sampling and analysis

The most common analytical instrument used to detect and quantify UV filters was liquid chromatography coupled with tandem mass spectrometry (LC‐MS/MS), accounting for 75% of studies, with the remainder analyzed by gas chromatography‐MS. Details on all analytical detection and extraction methods used for seawater analyses can be found in Supplemental Data, Table S3. 33% of these studies were based on the methods reported by Tsui et al. (2014). Limits of detection (LOD) were largely similar among studies, although varying levels of analyte recovery (as percentages) were observed when reported (Supplemental Data, Table S4). Differences in recovery could be explained by the specific extraction method employed (see Cadena‐Aizaga et al. 2020). Ideally, recovery should fall between 70 and 120%, and outside this range the reliability of results is impaired (Boix et al. 2015). Bargar et al. (2015) noted significant analyte losses for 3 analytes (ethylhexyl dimethyl para‐aminobenzoic acid [EDP], octocrylene [OC], and homosalate [HMS]), with recoveries falling below 11%, and so did not correct data sets for analyte recovery. Tsui et al. (2014) reported acceptable recoveries for all compounds except EHS (63%), whereas Mitchelmore et al. (2019) reported low recoveries for both EHS and HMS in seawater from some sites, although actual recoveries for all UV filters were not provided. Interestingly, analytical difficulties with both EHS and HMS were also reported in a recent freshwater study by O'Malley et al. (2021); neither could be quantified because of poor calibration and sensitivity resulting in LODs >10 000 ng/L.

**Figure 2:**
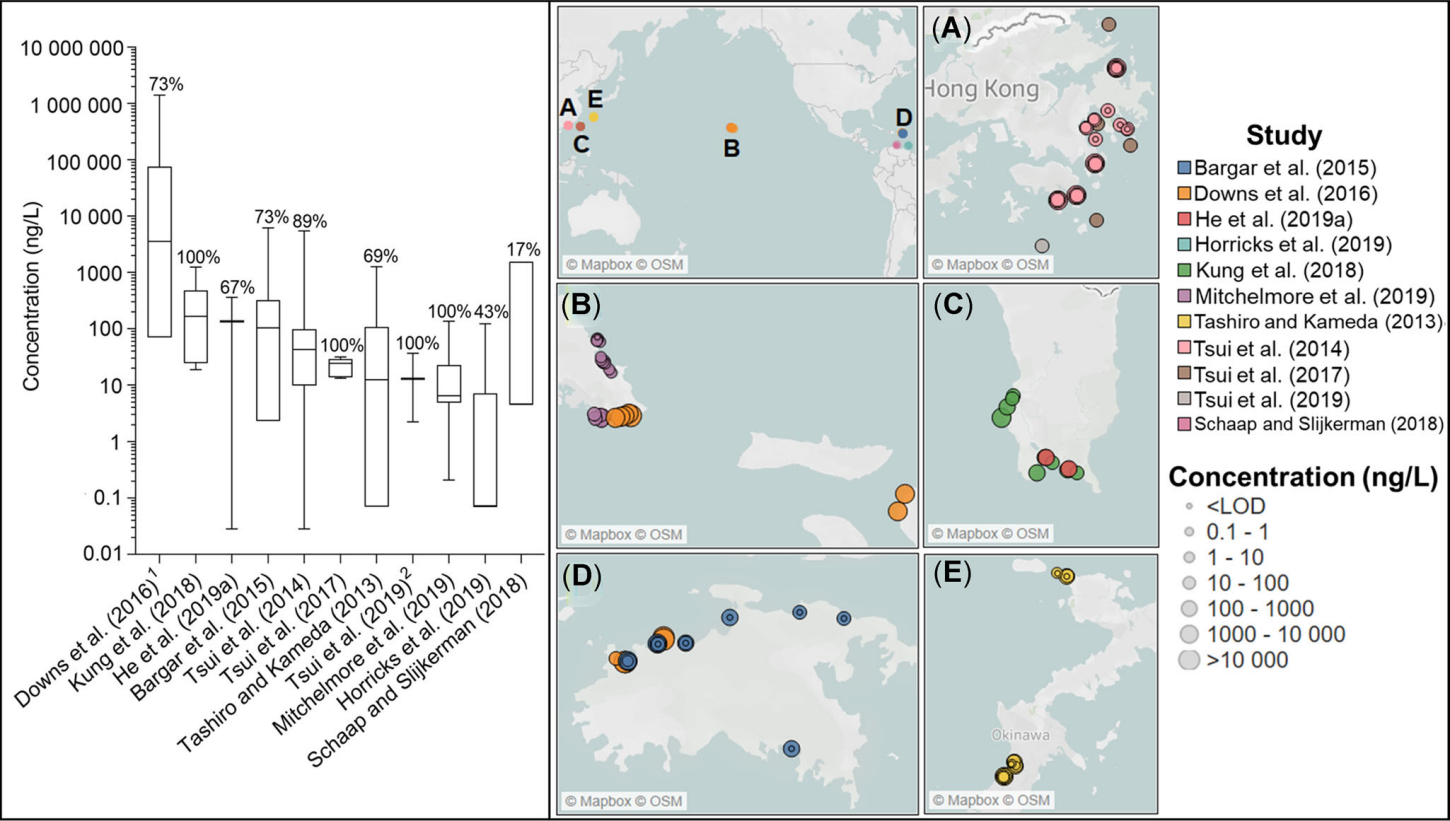
Summary of oxybenzone near‐reef water column concentrations globally. Left: Box plots include the median and are comprised of the minimum to maximum sample concentrations reported in each study (for values, see Supplemental Data, Table S2). The percentage detection frequency is provided above each box. Samples per study: *n* = 12, Downs et al. (2015); *n* = 8, Kung et al. (2018); *n* = 3, He et al. (2019b); *n* = 22, Bargar et al. (2015); *n* = 36, Tsui et al. (2014); *n* = 7, Tsui et al. (2017); *n* = 32, Tashiro and Kameda (2013); *n* = 36, Tsui et al. (2019); *n* = 19, Mitchelmore et al. (2019); *n* = 7, Horricks et al. (2019). 1) Six values fell between the limit of detection (100 ng/L) and the limit of quantification (5000 ng/L); 2) 36 samples were collected, but the paper only reported median, range, and detection frequency. Right: Global distribution of oxybenzone samples collected: (**A**) Hong Kong, (**B**) Hawaii, (**C**) Taiwan, (**D**) US Virgin Islands, and (**E**) Japan. Circle colors correspond to the study, while size provides an indication of concentration magnitude. LOD = limit of detection.

The reporting of key elements needed to determine the reliability of the analytical methods and, hence, data quality varies. The LOD or method detection limit (MDL) was either missing (Horricks et al. 2019) or reported as a range for all compounds (Goksøyr et al. 2009; Tashiro and Kameda 2013; Kung et al. 2018), both of which are problematic because an LOD is essential to put nondetect results in context or to left‐censor data (Antweiler 2015). Also absent from several studies was a limit of quantification (LOQ), the lowest concentration at which acceptable repeatability of measurements is achieved. Many calculations are used to generate LOQs, but generally they are 2 to 5 times higher than the LOD (Furlong et al. 2014; Supplemental Data, Text S2 and Table S4). The study of Downs et al. (2016) exemplifies why defining both parameters is critical. For US Virgin Island seawater samples analyzed via LC‐MS, the reported LOD was 100 ng/L, whereas the LOQ was 50 times higher (5000 ng/L). Firstly, this is an extraordinarily large margin between these parameters; and secondly, it meant that 46% of their samples could not be confidently quantified, just detected, albeit at much higher concentrations than reported in other studies (Figure 2). Another common issue was a lack of blank reporting, necessary to distinguish cross‐laboratory or field contamination. Only 25% of studies reported a methodological blank, whereas a field blank was only reported in a single study (Mitchelmore et al. 2019). Furthermore, matrix spikes, essential for quantifying matrix effects that commonly occur in environmental samples and can significantly impair the reliability of quantitative results, were conducted in only 50% of studies (see Supplemental Data, Table S3).

Important components of analytical method quality assurance and control, such as internal standards, standard addition, or matrix‐matched calibration, were lacking in 5 studies, raising concerns over the reliability of their results (i.e., Tashiro and Kameda 2013; Downs et al. 2016; Shaap and Slijkerman 2016; Kung et al. 2018; Horricks et al. 2019). Issues with sample storage and collection were also noted. For example, precleaned glass bottles should be used to collect organic contaminant samples to reduce the possibility of losses due to sorption to the container walls or leaching of chemicals from the container. Horricks et al. (2019) reported using a plastic container, whereas this detail was absent from 2 studies (Tashiro and Kameda 2013; Tsui et al. 2017). Furthermore, storage temperatures and timing between collection, processing, and analysis are often not reported. In some studies, excessive storage times may have influenced the results (e.g., Tsui et al. 2019).

Another major analytical issue is whether concentrations reported in seawater represent the total or dissolved fraction. This is particularly important because several of the UV filters (e.g., OC, EHMC, EHS, and HMS) are highly hydrophobic (log *K*_OW_ > 6), indicating a high likelihood of partitioning to particulates or organic matter. Method 1694 (US Environmental Protection Agency 2007) recommends filtration if there are any visible particles and that the sample should be treated as a 2‐phase sample, dissolved and particulate fractions. Many studies did not conduct this filtration or report whether filtration had been conducted (see Supplemental Data, Table S3). Therefore, comparisons between studies are complicated because hydrophobic UV filters are likely to be present in both the dissolved and the particulate fractions (Benedé et al. 2014). Extracting whole (total fraction) water samples represents an unknown exposure given that the proportion from each fraction is not known and analytical issues including column blockage or incomplete extraction may occur. Furthermore, bioavailability for filter feeders (e.g., corals) could constitute both fractions. Ultimately, implications include the under‐ or overreporting of coral exposure and ultimately toxicity through passive or dietary routes. However, it is common for toxicity thresholds to be related to the dissolved fraction (e.g., Organisation for Economic Co‐operation and Development [OECD] standard tests on daphnia, fish, and algae) because this is considered to be the readily bioavailable fraction.

Although sampling designs varied, all studies collected individual grab samples, in most cases without replication (Supplemental Data, Table S2). This is problematic because replicate samples are necessary to help determine whether sample contamination or degradation has occurred during collection, storage, processing, and analysis. In addition, replicates capture the inherent variability within the system over short time frames and/or distances, improving sample representativeness (US Geological Survey 2006). The grab sampling approach is considered a “snapshot sampling” (Imhof et al. 2017); therefore, it is important to improve the representativeness of this approach by including replicates. To date, only 2 studies have included sample replicates: Tsui et al. (2014) used duplicates, and Mitchelmore et al. (2019) used a triplicated sampling regime at each site (Supplemental Data, Tables S2 and S3).

Overall significant issues were identified in the reporting and sampling design of several monitoring studies, reducing confidence in the concentrations reported and significantly diminishing their appropriateness for characterizing exposure within a risk assessment (Leonards et al. 2013).

#### Occurrence of organic UV filters in seawater near coral reefs

The occurrence (exposure) data for UV filters in seawater near coral reefs are mainly shallow and nearshore; 2 studies (Tsui et al. 2017; Horricks et al. 2019) reported concentrations at the depth of the coral, and an additional 3 studies collected surface microlayer samples (Goksøyr et al. 2009; Bargar et al. 2015; Schaap and Slijkerman 2018). In total, the occurrence of 14 UV filters has been investigated, with BP‐3 being the only compound to be included in all studies (see Supplemental Data, Table S2).

**Figure 3:**
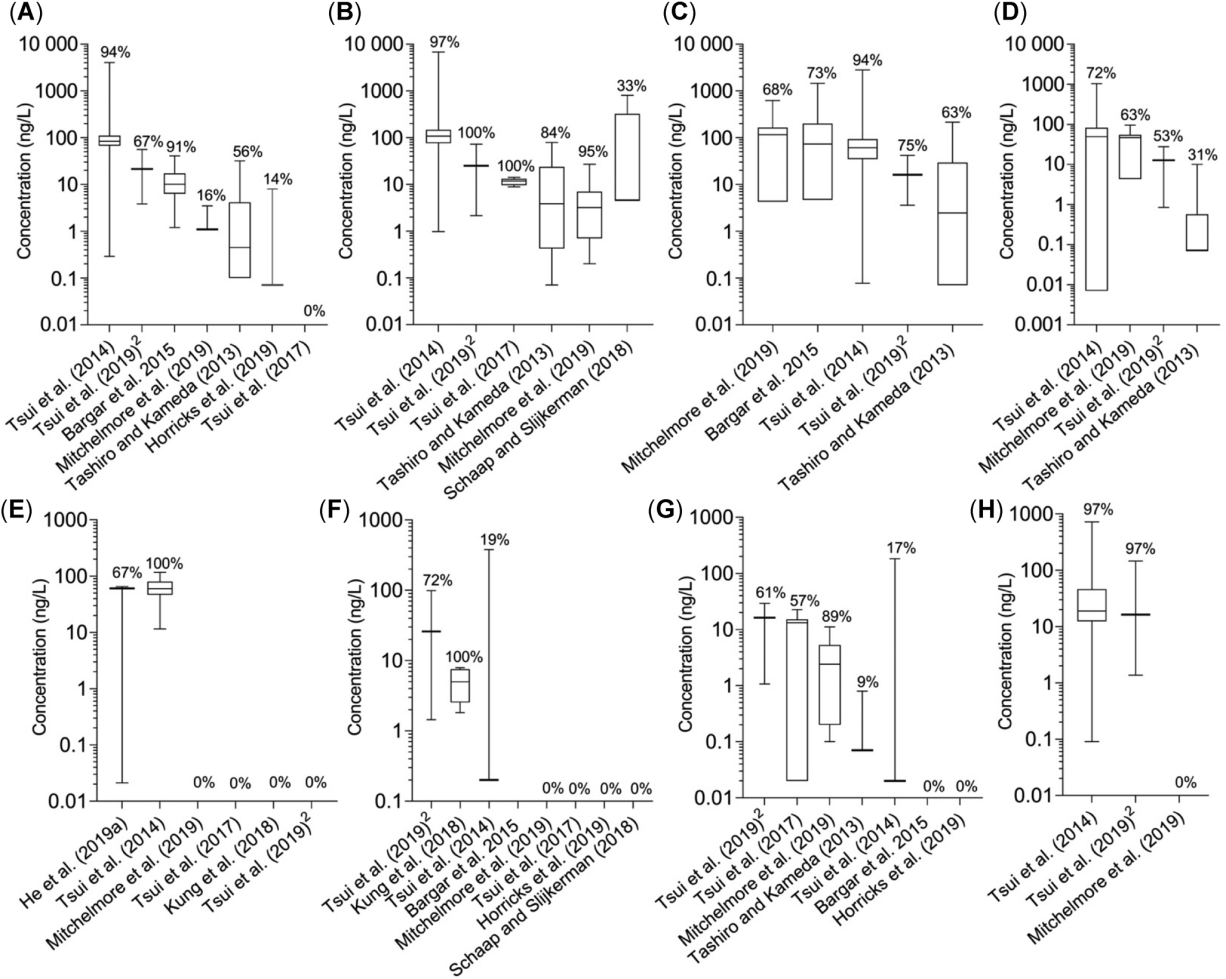
Summary of near‐reef water column ultraviolet (UV) filter concentrations globally for (**A**) ethylhexyl methoxycinnamate, (**B**) octocrylene, (**C**) homosalate, (**D**) ethylhexyl salicylate, (**E**) benzophenone‐8, (**F**) 4‐methylbenzylidene camphor, (**G**) ethylhexyl dimethyl para‐aminobenzoic acid, and (**H**) avobenzone. Box plots include the median and are comprised of the minimum to maximum sample concentrations reported in each study (for values, see Supplemental Data, Table S2). The percentage detection frequency is provided above each box. Samples per study: *n* = 12, Downs et al. (2015); *n* = 8, Kung et al. (2018); *n* = 3, He et al. (2019b); *n* = 22, Bargar et al. (2015); *n* = 36, Tsui et al. (2014); *n* = 7, Tsui et al. (2017); *n* = 32, Tashiro and Kameda (2013); *n* = 36, Tsui et al. (2019); *n* = 19, Mitchelmore et al. (2019); *n* = 7, Horricks et al. (2019). 1) Six values fell between the limit of detection (100 ng/L) and the limit of quantification (5000 ng/L); 2) 36 samples were collected, but the paper only reported median, range, and detection frequency. The UV filter abbreviations are listed in Table 1.

Chemical monitoring (exposure) data for BP‐3 are summarized in the left panel of Figure 2 and the global distribution of these measurements in the right panel (e.g., Figure 2A–E). The average detection frequency of BP‐3 per study was 76%, making it the second most frequently detected compound; OC was higher, 85% (Figure 3B). The median concentration of BP‐3 generally fell roughly between 1 and 100 ng/L, with a couple exceptions (e.g., Downs et al. 2016; Kung et al. 2018), indicating that in general BP‐3 concentrations near reefs are low. Besides Downs et al. (2016), Tsui et al. (2014) reported the largest concentration range at their near reef sites near Hong Kong, but this level of variability was not observed in their later sampling of similar sites (Tsui et al. 2019). The study by Downs et al. (2016) appears to be an outlier because their 5 quantitative detections range from 1 to 3 orders of magnitude higher than any other BP‐3 measurement. Mitchelmore et al. (2019) studied similar sites in Hawaii and Bargar et al. (2015) in the US Virgin Islands (Figure 2B). Both reported significantly lower maximum BP‐3 concentrations, 143 and 6143 ng/L, respectively, compared with Downs et al. (2016) at 19 200 and 1 395 000 ng/L for Hawaii and the US Virgin Islands, respectively. This latter value is exceptionally high given that total dissolved organic carbon in seawater near coral reefs typically ranges from 0.8 to 1.0 mg/L (Hata et al. 2002; Yahel et al. 2003; de Goeij and van Duyl 2007; Nelson et al. 2011; Tanaka et al. 2011).

The remaining UV filter concentrations reported per study are summarized in Figure 3. The average detection frequency was variable between studies and compounds, ranging from 28% (BP‐8) to 85% (OC), but mainly they were below 60%. The only compounds with detections that exceeded 1000 ng/L (with the exception of BP‐3) were EHMC, OC, and HMS, all reported by Tsui et al. (2014) and, in the case of HMS, Bargar et al. (2015). The variable detection frequencies could be indicative of regional patterns of UV filter occurrence, possibly driven by differences in UV filter usage and/or environmental inputs. The median concentration for all compounds in all studies conducted fell near or below 100 ng/L, indicating that for all UV filters studied thus far, concentrations present near coral reefs are in the low nanograms per liter range (Figure 3; Supplemental Data, Table S2). However, it is clear that there is high variability among samples and studies. This variability could be attributed to numerous factors including sampling location, time, depth, and sample collection and analysis. A handful of authors have attempted to attribute their observed UV filter variability to anthropogenic activity. For example, Tsui et al. (2014) found higher concentrations in the dry as opposed to the wet season, which correlated with recreational activity. Bargar et al. (2015) also reported higher values in the high‐ versus low‐tourist season, and Mitchelmore et al. (2019) observed the highest concentration of BP‐3 at the most recreationally impacted site, Waikiki Beach (Oahu, HI, USA). On the other hand, the correlation between beachgoers and UV filter concentrations was not always present. For example, some of the highest concentrations of OC, EHS, and HMS were observed at a site where no people were present (Mitchelmore et al. 2019), which suggests other sources of UV filters at that location. Although temporal variations have been shown, seasonal and finer‐scale temporal analyses (i.e., diurnal) have been limited (e.g., Labille et al. 2020).

Although limited, investigations into sampling depth and distance from shore have also been conducted, highlighting spatial variation in UV filters. Two studies found concentrations decreasing with increasing distance from shore (Bargar et al. 2015; Mitchelmore et al. 2019). Tsui et al. (2017) found that BP‐3 concentrations at coral depth were 30 times less compared with their previous surface water study (i.e., Tsui et al. 2014), highlighting that using surface concentrations to estimate coral exposure is likely an overestimate and therefore conservative. It has been suggested that UV filter concentrations would be highest at the surface microlayer, but the evidence to date is limited and unclear (e.g., Goksøyr et al. 2009; Bargar et al. 2015; Schaap and Slijkerman 2018). The majority of coral reefs would not be exposed to this layer except possibly in shallow, well‐mixed, high‐energy locations; but the surface microlayer has direct implications for coral larvae because gametes and larvae are released into the very shallow surface layers at night during spawning activities (Downs et al. 2016; Schaap and Slijkerman 2018). These findings have implications for the robust design of coral monitoring programs because samples collected in the nearshore beach zone may not reflect those concentrations at reefs located farther from the immediate beach area, particularly in open and well‐mixed, high‐energy coastal locations. Shorter‐term temporal trends (e.g., diurnal) should also be considered, to gain a better understanding of local environmental fate (e.g., degradation, flushing time) and assessing exposure during key spawning activities in the surface microlayer.

#### Occurrence of UV filters in sediment

Currently, there are far fewer monitoring data available for UV filters in marine sediments rather than the water column, and they are summarized in Table 2 (also see Supplemental Data, Text S6). Coral uptake from sediment could be an important pathway of UV filter exposure, particularly in areas of high wave activity where sediments can be continually resuspended. Overall, 12 UV filters have been investigated in sediment among the 4 studies, with concentrations generally in the low to subnanograms per gram dry weight range. Tsui et al. (2015) reported the highest maximum sediment concentrations: EHMC (447 ng/g dry wt) followed by EDP (150 ng/g dry wt). Tsui et al. (2015) also reported the highest median sediment concentrations across studied UV filters with only one, EDP, exceeding 10 ng/g dry weight. A consistent pattern of detection for any one compound does not emerge across the studies; however, OC was the only compound detected frequently enough for the median concentration to be above the LOD in each study (Table 2). Generally, the more hydrophobic UV filters (EHMC, EDP, OC, and EHS) were more frequently detected, but this was not consistent. For example, HMS and EHS (log *K*_OW_ > 6.3) were detected in 100% of samples by Mitchelmore et al. (2019) but at a much lower frequency and concentration by Apel et al. (2019) and not at all by Tsui et al. (2015, 2017). This could in part be due to regional differences in UV filter emissions between these studies. The type of sediment sampled may also play a role. For example, Mitchelmore et al. (2019) reported no correlation between seawater and sediment concentrations. However, sediment concentrations did significantly differ between sites, likely as a result of variable sediment organic fractions. Mitchelmore et al. (2019) anecdotally observed that finer, siltier, more organic sediment corresponded with higher concentrations of many UV filters compared to the more sandy, organic material–poor sediments at the other sites.

Overall, in sediments more hydrophobic compounds appear in higher concentrations, but one of the least hydrophobic UV filters studied, BP‐3 (log *K*_OW_ 3.45), was detected in all 4 of the studies. Many questions in terms of UV filter sediment exposure and fate are yet to be answered, particularly from a risk‐assessment perspective where the implication of the sediment exposure pathway is unknown.

#### Occurrence of UV filters in coral

Concentrations found in the tissues of resident aquatic organisms can be seen as a necessary prerequisite for an adverse effect to occur. There are only 2 studies that have investigated the concentration of UV filters in field‐collected coral tissues (Table 3; Supplemental Data, Text S7; Tsui et al. 2017; Mitchelmore et al. 2019). Oxybenzone was detected in 100% of the coral samples collected by both authors, but neither study found a median concentration above detection limits for EHMC, EDP, 4‐methylbenzylidene camphor (4‐MBC), BP‐4, triethanolamine salt of salicylate, or phenylbenzimidazole sulfonic acid. This could be explained by relatively lower concentrations and more nondetects in the water column compared to BP‐3 (Figure 3). Further, relative coral tissue concentrations of EHS and HMS coincide with the trends observed in the water column (i.e., highest medians) in Mitchelmore et al. (2019), but they also have the highest log *K*_OW_ (i.e., >6.3). In contrast to Mitchelmore et al. (2019), Tsui et al. (2017) recorded fewer coral tissue UV filter detections for OC and 4‐MBC; but direct concentration comparisons are complicated because of different reporting values (i.e., concentration per dry or wet wt of coral tissue). When corrected to wet weight, similar tissue concentrations emerged despite regional differences in sampling location and species (see Mitchelmore et al. 2019). For example, BP‐3 concentrations per site reported by Mitchelmore et al. (2019) ranged from 5.8 to 241 ng/g dry weight (estimated as 1.1–46.3 ng/g wet wt). This is similar to the 2.8 to 31.8 ng/g wet weight range reported for BP‐3 by Tsui et al. (2017). The second highest detection and concentration reported by Tsui et al. (2017) was for BP‐8 (i.e., up to 24.7 ng/g; detection frequency 86%), which was not detected by Mitchelmore et al. (2019). It is possible that UV filter concentrations in corals may be influenced by biotic factors relating to the coral species or time of year; for example, lipid content, an important factor in driving uptake of organic chemicals, is known to vary both temporally and between species of coral (Imbs 2013). Furthermore, differences may relate to metabolism, as suggested by Tsui et al (2017), with BP‐8 and BP‐1 being derived from BP‐3. Additional research is recommended to address this metabolic potential and to determine the toxicity of these products in comparison to the parent compounds. Bioaccumulation of UV filters is beyond the scope of this review because modeling approaches and controlled laboratory uptake and depuration studies in coral species (e.g., Pawlowski et al. 2019) need to be conducted and analyzed along with the field data presented herein prior to refining and estimating coral uptake and bioaccumulation.

## TOXICITY OF ORGANIC UV FILTERS TO CORAL

A total of 9 studies (6 since 2019) have investigated the toxicity of organic UV filters on larval and adult (fragments/nubbins) life stages of intact corals employing a range of biological endpoints including mortality, growth, photosynthetic yield, and most commonly, bleaching (Supplemental Data, Table S5). Coral is a nonstandard ecotoxicological test species, and no standardized guidelines exist to conduct toxicity tests and identify or quantify the responses to chemical exposure. Furthermore, coral availability, permitting requirements, and suitability to laboratory culture may limit their use as well. In the present section we review the nature of coral toxicological test systems and the results obtained for UV filters.

#### Exposure and test conditions

**Figure 4:**
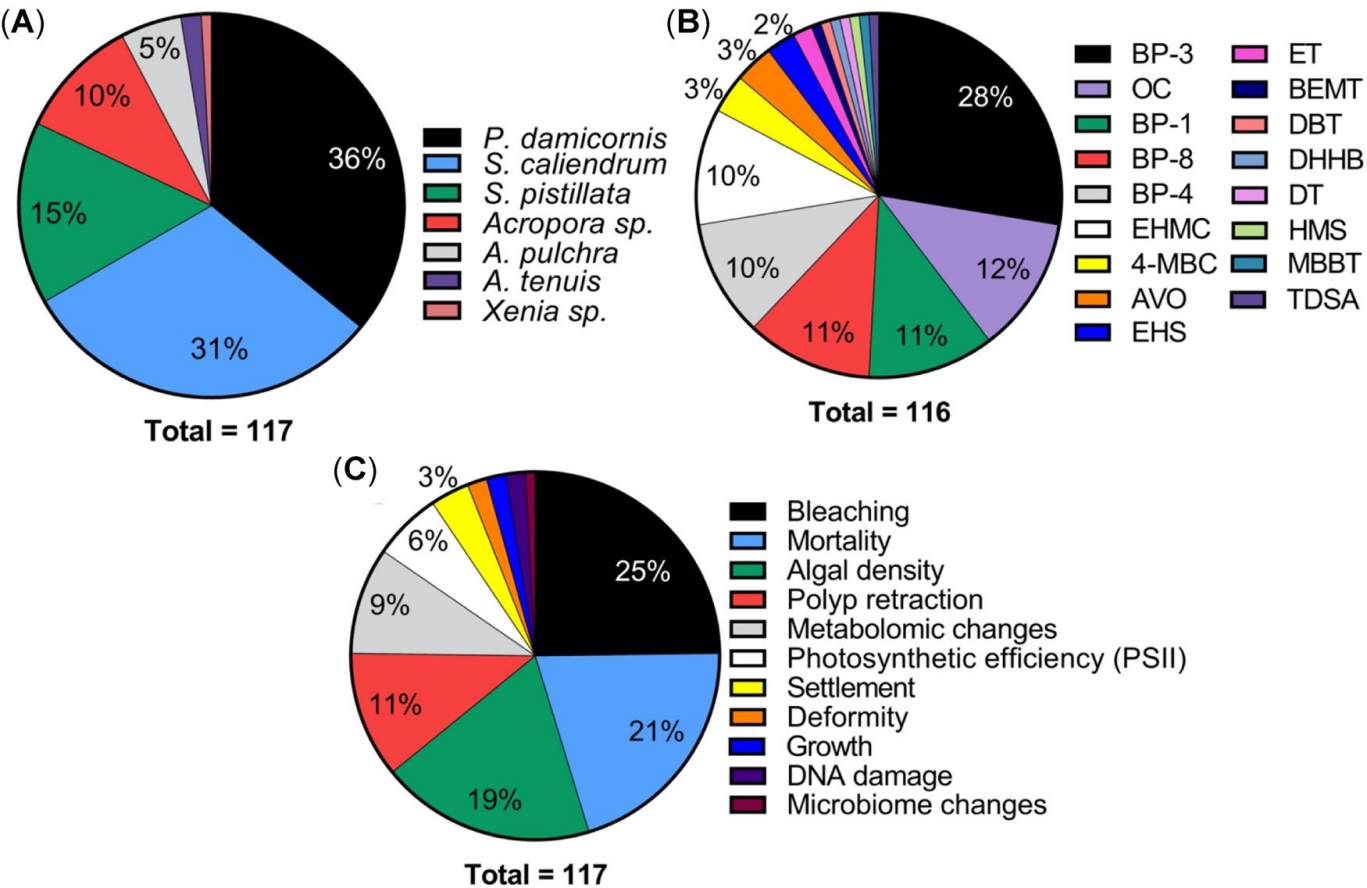
Summary pie charts of coral toxicity test characteristics including (**A**) species tested, (**B**) endpoints per ultraviolet (UV) filter, and (**C**) endpoints studied. Note: McCoshum et al. (2019) could not be included in plot B because they used a formulation which included multiple UV filters in their experiment. Species are *Pocillopora damicornis*, *Seriatopora caliendrum*, *Stylophora pistillata*, *Acropora pulchra*, and *Acropora tenuis*. AVO = avobenzone; BEMT = bis‐ethylhexyloxyphenol methoxyphenyl triazine; BP = benzophenone; DBT = diethylhexyl butamido triazone; DHHB = diethylamino hydroxybenzoyl hexyl benzoate; DT = drometrizole trisiloxane; EHMC = ethylhexyl methoxycinnamate; EHS = ethylhexyl salicylate; ET = ethylhexyl triazone; HMS = homosalate; MBBT = methylene bis‐benzotriazolyl tetramethylbutylphenol; 4‐MBC =4‐methylbenzylidene camphor; OC = octocrylene; TDSA = terephthalylidene dicamphor sulfonic acid.

**Figure 5:**
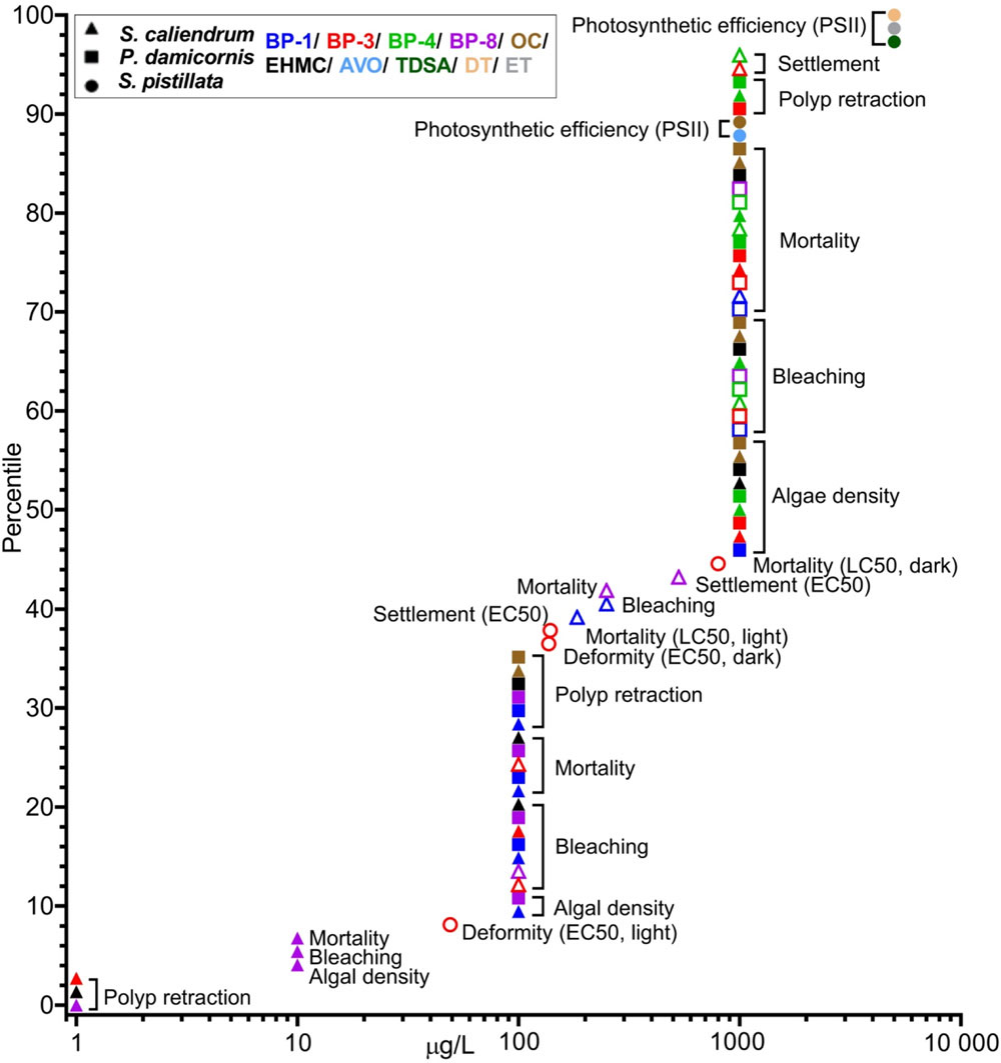
Coral cumulative endpoint distribution for all ultraviolet (UV) filters. Symbol shape indicates the test species, symbol color represents the test compound, and when a symbol is hollow it indicates the endpoint is a larva rather than adult. All endpoints are no‐observed‐effect concentrations (NOECs) unless otherwise stated in parentheses. A cumulative distribution can be interpreted as where along the *x*‐axis a NOEC is likely to fall. For example, the 25th percentile NOEC is approximately 100 µg/L, whereas the 75th is approximately 1000 µg/L. Endpoints pertain to both acute and chronic tests. Danovaro et al. (2008), McCoshum et al. (2019), Stien et al. (2019, 2020), and Wijgerde et al. (2020) did not derive suitable endpoints to include. See Supplemental Data for all endpoint details. All UV filter abbreviations are listed in Table 1. Species are *Pocillopora damicornis, Seriatopora caliendrum*, and *Stylophora pistillata*. AVO = avobenzone; BP = benzophenone; DT = drometrizole trisilioxane; EC50 = median effect concentration; EHMC = ethylhexyl methoxycinnamate; ET = ethylhexyl triazone; LC50 = median lethal concentration; OC = octocrylene; TDSA = terephthalylidene dicamphor sulfonic acid.

Toxicity studies have been conducted in 7 coral species using either adult fragments, early‐life stages (i.e., larvae/planula), or both. They include short‐term (acute) and longer‐term (chronic) exposures, although the concentrations used, exposure times, solution renewal, and endpoints assessed vary significantly among studies (see Figures 4 and 5; Supplemental Data, Table S5). The majority of experiments were conducted using *Pocillopora damicornis* (36% of endpoints) and *Seriatopora caliendrum* (31% of endpoints; Figure 4), which can be mainly be attributed to the studies of He et al. (2019a, 2019b) although Stien et al. (2019, 2020) also worked with *P. damicornis*. Fel et al. (2019), Downs et al. (2016), and Wijgerde et al. (2020) all worked with *Stylophora pistillata*. Danovaro et al. (2008) chose to work mainly with *Acropora* at the genus level, whereas Wijgerde et al. (2020) worked with *Acropora tenuis*. The variety of species and life stages tested is beneficial from a risk‐assessment perspective because they provide insight into the interspecies and life‐stage sensitivity of coral to UV filter exposure. Corals are often described as sensitive compared to other marine species, although species sensitivity distribution curves have not always shown that (see Bejarano 2018). Furthermore, the early life stages of organisms have also been described as more sensitive compared to adults. Differential impact of UV filters in 2 species was highlighted by He et al. (2019a, 2019b), who also concluded that adult was the most sensitive life stage, in contrast to the discussion by Downs et al. (2016). Similarly, although both species were unimpacted by exposure to 0.06 µg/L BP‐3 (i.e., no mortality or impact on growth after 6‐wk exposure), Wijgerde et al. (2020) demonstrated that *A. tenuis* was more sensitive to temperature elevations than was *S. pistillata*.

In terms of number of studies and endpoints reported, BP‐3 was studied the most often (i.e., in 67% of the studies, 28% of endpoints; Figure 4B), followed closely by OC, BP‐8, and BP‐1, largely because of the numerous ecotoxicological responses studied by He et al. (2019b) in both larvae and adults in 2 coral species. Five different authors studied OC, resulting in 12% of the total endpoints in Figure 4B. On the other hand, EHMC, despite being included in regulatory developments surrounding UV filters and coral, has only appeared in 2 studies (i.e., 22% of studies and 10% of endpoints in Figure 4B) to date (Danovaro et al. 2008; He et al. 2019a).

A variety of acute (i.e., mortality) and chronic endpoints were studied in the toxicity tests; however, the duration of these tests were variable, and the justification for these durations, biological endpoints, and toxicity thresholds reported is unclear. For example, the duration of the chronic tests varied from 35 to 41 d, and endpoints included mortality, growth, algal density, microbiome changes, and photosynthetic efficiency (Fel et al. 2019; Wijgerde et al. 2020). Meanwhile acute studies ranged from 8 h to 14 d and included the endpoints of mortality, larval settlement, algal density, bleaching, deformity, DNA damage, metabolomic changes, and polyp retraction (Danovaro et al. 2008; Downs et al. 2016; He et al. 2019a, 2019b; Stien et al. 2019, 2020). Chronic studies aim to capture a representative portion of a sensitive life stage and generally study sublethal endpoints pertaining to reproduction and growth. For example, 7‐ to 21‐d tests are conducted for invertebrates (Organisation for Economic Co‐operation and Development 2012; US Environmental Protection Agency 2016), 4‐d tests for algae (Organisation for Economic Co‐operation and Development 2011), and approximately 30‐d tests for fish (Organisation for Economic Co‐operation and Development 1992); and either a NOEC or an EC10 is derived. Interestingly, a growth endpoint was included in only 2 studies (McCoshum et al. 2016; Wijgerede et al. 2020), whereas He et al. (2019b) included endpoints related to reproduction in their acute exposures (i.e., larval settlement). The He et al. (2019a, 2019b) experiments, despite being 7 to 14 d in length, appear to be defined as an acute study given that assessment factors to convert their data in the risk assessment are used. In contrast, acute endpoints are normally associated with short‐term, up to 4‐d, exposures and aim to derive either an EC50 or more usually an LC50. Mortality was included in longer‐term exposures, for example, by He et al. (2019a, 2019b) in their 7‐ to 14‐d tests in both larvae and adult corals and by Wijgerde et al. (2020) in their 41‐d study. Meanwhile, Fel et al. (2019) reported mortality but instead as a validity criterion for their 5‐wk exposures rather than a NOEC or an LC50. Meanwhile, Downs et al. (2016) characterized mortality in coral larvae exposed to BP‐3 in an acute exposure (24 h), whereas Danovaro et al. (2008) failed to report exposure duration for characterization of their bleaching endpoint entirely. These differences in test length could be necessary depending on the particular coral species; however, studying lethal and sublethal endpoints over the same test duration signals an exploratory investigation rather than a definitive toxicological test.

The specific design of the toxicity test directly impacts the exposure concentration and duration of exposure, which are critical variables in defining toxicity. How test exposure solutions are made and how often they are renewed or maintained ultimately influence the concentration that the organism is exposed to, particularly for chemicals that may rapidly hydrolyze, photodegrade, or bind to test chambers or are taken up rapidly by the test organism. The majority of coral UV filter studies (89%) have employed a static (no renewal of test solution; e.g., Downs et al. 2016; He et al. 2019a, 2019b) or a static renewal‐type exposure (i.e., once a week; Fel et al. 2019). This is problematic for maintaining analyte concentration and potentially coral health (discussed in the section *Common methodological issues with characterizing UV filter hazard to coral*). An exception to this is the study by Wijgerde et al. (2020), which states using a flow‐through exposure system; however, the setup reported by the authors indicates a static renewal. A typical flow‐through system would renew the new test solutions for a complete exposure renewal of 100% a number of times a day and would be beneficial because it would reduce the complications of UV filter loss and potential increases in degradates/metabolites and maintain water quality parameters for optimal coral health.

#### Common methodological issues with characterizing UV filter hazard to coral

After reviewing the study designs and methodological details of the limited number of coral ecotoxicological studies, several major themes emerged that together could limit their usefulness for reliably characterizing the impact of UV filters on coral and thus their adequacy for risk assessment and ultimately decision‐making.

One of the most significant problems identified with the ecotoxicity studies was a lack of analytical verification of exposure concentrations with the exception of the 2019 and 2020 studies, although even these studies conducted verification at a frequency that does not permit accurate assessment of the exposure concentration over the duration of the exposure (Fel et al. 2019; He et al. 2019a, 2019b; Wijgerde et al. 2020). For example, Fel et al. (2019) collected a sample 2 h after analyte introduction to the test system (which was once a week), but the frequency of sample collection beyond that is unclear because it varied from 3 to 20 measures over the 5 wk depending on the UV filter under study. This is key information because significant losses in several analytes were observed, for example, 91 and 48% of avobenzone (AVO) and OC, respectively, from an initial 1000 µg/L exposure. He et al. (2019b) sampled at the beginning (day 0) and the end of the 7‐d adult and 14‐d larvae experiments. They demonstrated that day 0 values closely matched nominal values for the larval exposures, although they ranged from 100 to 280% in the adult tests. In the adult exposure after 7 d, all BP‐4, BP‐1, BP‐3, and BP‐8 treatments (i.e., up to 1000 µg/L) were <LOD. Similarly, He et al. (2019a) reported day 0 concentrations 90–265% of nominal, but loss over time was again apparent because EHMC concentrations were 50 to 87% of nominal on day 1 and <LOD to 2% on day 7. Loss of OC was not as great: at concentrations of 1 µg/L and higher values were 66 to 200% at day 1 and 24 to 61% at day 7. Loss of BP‐3 was also observed in the Wijgerde et al. (2020) study, reporting a measured value of 0.06 compared to the nominal 1 µg/L.

These results indicate 2 issues that need to be addressed in future toxicity testing. Firstly, UV filters are significantly lost from test systems, and strategies to maintain exposure concentrations throughout the test need to be undertaken. Otherwise, efforts to observe a significant dose–response relationship could become significantly hampered (Moermond et al. 2017). Secondly, the actual exposure concentration in studies that did not conduct analytical verification is unknown, which is particularly important considering the substantial derivations from nominal values at the start and end of tests observed. Because exposure is not known, over‐ or underestimations of toxicity could inadvertently be reported, substantially diminishing the reliability of the toxicity data for risk‐assessment purposes.

Further, compared to standard test species, coral are challenging organisms to keep healthy and thriving in a laboratory setting, particularly for chronic tests that often require longer durations for growth differences to be teased out. Usually hard coral symbiotic species are tested where the animal hosts are in tightly controlled symbiotic relationships with dinoflagellate algae of the genus *Symbiodinium* (in addition to bacteria forming the holobiont association). Stressors to either one of the partners have significant impact on the other; for example, under stress corals often lose their algal symbionts, resulting in bleaching. Corals require specific lighting conditions, temperatures, feeding and nutrition, water quality and flow for optimal health, which are often species‐ and life stage–specific; and acceptable variations are often very tightly bound ranges (e.g., Watanabe et al. 2007). For example, light quantity, quality (spectral range), and photoperiod impact coral growth rate, symbiont density (bleaching), photosynthetic efficiency, oxidative stress, and DNA damage (e.g., see Smith and Birkeland 2007; Schutter et al. 2012; Kuanui et al. 2020). An essential element many adult corals require is adequate water flow, influencing bioenergetics (metabolic rates) and ultimately rate of growth (Sebens et al. 2003; Smith and Birkland 2007; Schutter et al. 2010). Only 2 studies (Fel et al. 2019; Wijgerde et al. 2020) maintained flow with pumps and the rate stated (i.e., 4 or 2.7 mL/min, respectively). The studies by He et al. (2019a, 2019b) and Stien et al. (2019, 2020) used bottles or beakers and <1 L of exposure medium that was aerated, although it is not clear if the bubbling of air provided any significant water flow. Strict temperature control and monitoring are also required during coral toxicity tests. Brown et al. (2000) discussed the additive or synergistic influence of warming seawater and pollutant exposure in the process of coral bleaching. Indeed, Amid et al. (2018) found additive impacts in *Acropora formosa* on photosynthetic capacity when temperature and herbicide exposures were combined. Recently, Wijgerde et al. (2020) also investigated the impact of elevated temperature and BP‐3 toxicity to highlight the complexity of multiple stressor effects in coral and the argument for multiscale coral reef management (i.e., local through global).

Only in 2 chronic studies were the corals fed (i.e., with *Artemia* sp. once a day or week in the Fel et al. [2019] and Wijgerde et al. [2020] studies, respectively), yet feeding is known to impact growth and calcification rates and other biological parameters often reported in coral toxicity tests (i.e., chlorophyll content as a proxy for bleaching, protein content; Ferrier‐Pagès et al. 2003). Overall these findings highlight the essential need for the monitoring and reporting of water quality and test conditions, which have been limited or missing in many studies to date. As with other standard tests, both negative and positive controls should be included to ensure that coral health and response are optimal and within expected test ranges. To date, no study has included a positive control or repeated an experiment to ensure reproducibility.

Ultraviolet filters are challenging compounds to work with, as evidenced by their substantial losses from coral test systems, as previously discussed in this section. Several UV filters are also poorly soluble (Table 1), so a solvent may be required to get them into solution at the high concentrations required for the test (Organisation for Economic Co‐operation and Development 2019b). Solvents could exert an unintended effect; therefore, a solvent control in addition to a negative control (i.e., seawater only) is needed to distinguish between any unintentional abnormalities or effects induced by solvent exposure. A number of solvents have been employed; 3 studies used methanol (Fel et al. 2019; He et al. 2019a, 2019b), whereas Danovaro et al. (2008) used propylene glycol. Three studies (Downs et al. 2016; Stien et al. 2019; Wijgerde et al. 2020) used dimethysulfoxide (DMSO), which is problematic because it can enhance biological uptake of the test substance, an issue that cannot be addressed with a solvent control. For example, Kais et al. (2013) found that >0.1% DMSO increased the uptake of fluorescein into fish embryos and recommended that a maximum of 0.01% DMSO be used in the fish embryo test, as indicated by the Organisation for Economic Co‐operation and Development (2019b). The studies by Stien et al. (2019, 2020) used very high concentrations of DMSO (i.e., 0.25% v/v concentration) in comparison to the 5 × 10^−4^% v/v used by Downs et al. (2016) and the 0.01% v/v used by Wijgerde et al. (2020), although it should be noted that the latter study did not include a negative control in its experimental design. A 33% mortality of *Acropora tenuis* was observed in the solvent control for the study; but without a true negative control (i.e., no solvent), it is unclear if this mortality is due to the solvent or just the unsuitability of the species to long‐term laboratory exposures. This brings back the need for the development of a standard toxicity test for coral species because a >20% mortality rate is unacceptable in any chronic standard invertebrate test species test (e.g., US Environmental Protection Agency 1996, 2016; Organisation for Economic Co‐operation and Development 2012). Furthermore, DMSO is a powerful antioxidant (Sunda et al. 2002); therefore, its inclusion in a test investigating oxidative stress (e.g., Downs et al. 2016) is a confounding factor.

Another problem identified was a lack of clarity over the concentration and/or mixture of sunscreen ingredients to which coral were exposed. The Danovaro et al. (2008) study is of limited utility because the concentrations of BP‐3 and EHMC are unclear; they report nominal volume to volume concentrations with no information on preparation or purity of the dosing stock. A similar problem was encountered with McCoshum et al. (2016), where volume to volume exposure concentrations of a sunscreen are reported. Their study design also renders their data of limited value given that the coral is exposed to 2 concentrations of a sunscreen product containing BP‐3, HMS, EHS, OC, and AVO, so it is impossible to determine whether any effects observed were specifically attributable to any particular UV filter and/or other inactive ingredients.

Only 2 of the 3 acute studies derived an EC50 or LC50 (Downs et al. 2016; He et al. 2019b). This is a result of the concentration spacing, number, and range of test treatments included. For example, He et al. (2019b) included a variety of endpoints using a single dosing range (0.1–1000 µg/L) and number of treatments which resulted in either a NOEC at the highest concentration tested (HNOEC) or a LOEC, with the exception of larval settlement which resulted in an EC50 for BP‐1 and BP‐8. This demonstrates that, for the majority of their endpoints, the dosing range needed to be adjusted for definitive statistical endpoint derivation (i.e., EC[LC]50). Indeed, the authors report that their exposure is acute based on their risk assessment and derivation of a single EC50. On the other hand, the length of their exposure (7–14 d) and the variety of sublethal endpoints mixed in with the lethal adult and larval endpoints provide further confusion over the nature of this test. This is an important consideration because chronic and acute data are used differently in a risk assessment; different endpoints are derived, NOEC and EC10 (chronic test) or EC(LC)50 (acute test); and assessment factors are used, which could significantly impact the outcome of a risk assessment. Downs et al. (2016) studied mortality and deformation in planulae, deriving an LC50 and EC50 under both light and dark conditions. Under light conditions, BP‐3 was more toxic, but it should be noted that the reliability of these endpoints is diminished because the test concentrations do not adequately bracket the EC50/LC50 (e.g., the endpoints fall between the 2 lowest concentrations tested, whereas all higher‐concentration treatments resulted in 100% mortality or deformation) impairing the accuracy of their results (Moermond et al. 2016). Furthermore, the study highlights statistical problems and toxicological data reporting inconsistencies between the text, figures and tables. For example, in 8‐h BP‐3 in the light exposures a sub‐lethal endpoint (i.e. deformation EC50s) occurs at higher concentrations than mortality (LC50s) and a 24‐h light LC50 is listed at 2 orders of magnitude lower (i.e., 1.39 µg/L) than reported in the text (see Table 1 in Downs et al. 2016). These study designs highlight the need for a range‐finding test to be followed by a definitive test where a suitable spacing factor and treatment number can be applied that will appropriately bracket statistical endpoints. It also signals a need for a clear definition of suitable chronic and acute endpoints and exposure durations to derive coral toxicity thresholds.

A final issue observed is a lack of environmental relevance. For example, Danovaro et al. (2008) placed coral fragments in sealed plastic bags containing ultrafiltered (0.02 µm) seawater and left them in situ so that important parameters like temperature, dissolved oxygen, light, and flowing water could not be controlled. Another aspect not discussed by authors, with the exception of Fel et al. (2019), was the relevance of their results considering solubility. Several endpoints exceed the solubility of the UV filter under study, a situation highly unlikely to occur in the environment. For example, He et al. (2019a) reported a polyp retraction LOEC in response to OC exposure of 1000 µg/L, while the solubility of OC in freshwater is 40 µg/L (Table 1). Lastly, Downs et al. (2016) reported data from a coral cell toxicity assay using isolated calicoblast cells to demonstrate coral species sensitivity. The 4‐h LC50 values observed in the cell lines were much lower than the LC50 observed for planulae by the author for the same species, 42 and 139 µg/L, respectively. The inclusion of in vitro data is a novel approach, but without a positive control to demonstrate that the assay works, the validity of the cell lines as a surrogate measure for whole‐coral toxicity is uncertain, especially considering the use of the solvent DMSO. Furthermore, calicoblast cells do not contain the symbiont (see Downs et al. 2010), and implications for the health of the intact coral containing multiple cell types need further investigation. The use of in vitro data rather than whole organisms will in future become an important aspect of environmental risk assessment, as discussed in the 21st‐century toxicity testing paradigm (National Academies of Sciences, Engineering, and Medicine 2017); however, much work needs to be done before this is a robust alternative to whole‐organism testing. For example, the fish embryo test (OECD 236; Organisation for Economic Co‐operation and Development 2013) has taken 10 yr to develop, and a strong correlation with the whole‐fish acute test (OECD 203; Organisation for Economic Co‐operation and Development 2019a) has been demonstrated. Despite this, the test has yet to achieve ubiquitous regulatory acceptance (Sobanska et al. 2018). In addition to in vitro data, molecular endpoints were reported by Downs et al. (2016), such as DNA damage, are of limited use in their current form because correlations with known toxicological outcomes are missing. On the other hand, Stien et al. (2020) recently published a metabolomic profiling technique to detect stress in coral nubbins exposed to emerging pollutants. Two out of the 10 UV filters studied showed a metabolomic signature related to a stress response. Similar to the molecular endpoints reported by Downs et al. (2016), this stress response is presented without any correlation to a measurable toxicological outcome; and therefore, the information the assay provides is of limited use in a risk‐assessment context.

More generally, positive controls would have been a welcome addition to all of the studies to demonstrate that coral respond appropriately to a known toxicant for a particular endpoint because these test systems are not well established. Moving forward, incorporating ecotoxicological good practice (e.g., Harris et al. 2014; Moermond et al. 2016) would significantly improve the quality of future studies, making them more appropriate for risk assessment and decision‐making.

### Distribution and types of ecotoxicity endpoints and their toxicological significance

The distribution of all ecotoxicity endpoints from the studies reviewed are presented together in Figure 5 irrespective of test type (acute or chronic), exposure time, and biological endpoint tested. All data points correspond to nominal exposures and are NOECs based on nominal exposures unless otherwise stated (i.e., LC50, EC50). In total, endpoints from only 4 studies could be included (Downs et al. 2016; Fel et al. 2019; He et al. 2019a, 2019b). As discussed, the nature of the He et al. (2019a, 2019b) studies is not clear (i.e., acute or chronic). Therefore, in the interest of providing a conservative distribution of coral toxicological thresholds, the data from these studies are reported as NOECs, with the exception of the EC50 for larval settlement for BP‐1 and BP‐8 (Figure 5). Two studies could not be included because the exposure concentration was not reported in mass to volume or the exposure was to a sunscreen formulation (Danovaro et al. 2008; McCoshum et al. 2016). Wijgerde et al. (2020) could not be included because it consisted of a single UV filter treatment concentration (also the case for McCoshum et al. 2016) and was not a limit test; therefore, no meaningful statistical endpoints can be derived (e.g., NOEC, LOEC). Another 2 studies only qualitatively evaluated a polyp retraction endpoint because they focused on metabolomic changes rather than deriving a statistical ecotoxicological endpoint (Stien et al. 2019, 2020). The cell line data reported by Downs et al. (2016) were not included in Figure 5 because the assays were not validated and it is currently unclear how in vitro data relate to whole‐organism data, and this should be further investigated. Bleaching and DNA damage (8 h) NOECs were not included because the appropriate toxicological endpoint (EC50) could not be calculated. Conversely, the 24‐h deformity (EC50) and mortality (LC50) endpoints are included rather than the 8‐h counterparts because of problematic results and shorter test duration (discussed previously in the section *Common methodological issues with characterizing UV filter hazard to coral*).

The majority of NOECs from the reported endpoints fall within the range of 1 to 1000 µg/L (Figure 5). This is influenced heavily by the few studies that are available and the concentrations the authors chose to test. For example, of the 75 endpoints in Figure 5, 88% were derived by He et al. (2019a, 2019b). Based on the currently available data, *S. caliendrum* (triangles, Figure 5) appears to be more sensitive to UV filter exposure than *P. damicornis* (squares), for which a similar data set is available (He et al. 2019a, 2019b). The most sensitive endpoint for BP‐3, BP‐8, and EHMC was *S. caliendrum* polyp retraction (i.e., LOEC 10 µg/L, NOEC 1 µg/L; He et al. 2019a, 2019b). This is closely followed by algal density, bleaching, and mortality in *S. caliendrum* in response to BP‐8 exposure and *S. pistillata* deformity (24‐h EC50 in light) in response to BP‐3 exposure as reported by Downs et al. (2016). In terms of settlement, *S. caliendrum* larvae appear to be more sensitive to BP‐1 and BP‐8 (184 and 530 µg/L EC50, respectively) rather than BP‐3 or BP‐4 (1000 µg/L NOEC). With the exception of bleaching when exposed to BP‐3, adult coral do appear to be more sensitive to UV filter exposure; however, this trend is based on limited data. This result is surprising because it is usually the early life stages of species that are more sensitive to contaminants. This point was discussed in Downs et al. (2016), where the 24‐h LC50 for *S. pistillata* planulae exposed to BP‐3 in the light was 139 µg/L, although this is more sensitive than the LC50s for larval and adult corals exposed to BP‐3 in the He et al. (2019b) study (i.e., LC50 > 1000 µg/L) for longer‐duration exposures (7–14 d). Furthermore, the response of adult *S. pistillata* exposed to BP‐3 was recently investigated by Wijgerde et al. (2020), who found no mortality after a 6‐wk exposure to 0.06 µg/L measured (1 µg/L nominal). The least sensitive endpoint appears to be photosynthetic efficiency; Fel et al. (2019) reported a 5000 µg/L NOEC for terephthalylidene dicamphor sulfonic acid (TDSA), drometrizole trisiloxane (DT), and ethylhexyl triazone (ET), organic UV filters that are on the European Union market but not currently authorized for use in the United States (Table 1). Meanwhile, the AVO NOEC for photosynthetic efficiency, which is authorized for use in the United States, was 1000 µg/L (87 µg/L measured), which exceeds solubility for this compound (27 µg/L; Table 1). Unfortunately, data for this endpoint have not been reported for other UV filters for comparison. Overall, limited trends emerge from the ecotoxicity endpoint distribution, but generally, BP‐8 appears to be the most potent. What is clear is that many of the endpoints reported correspond to concentrations greater than the solubility of the UV filter (Table 1), indicating a very low likelihood of occurring in the environment.

**Figure 6:**
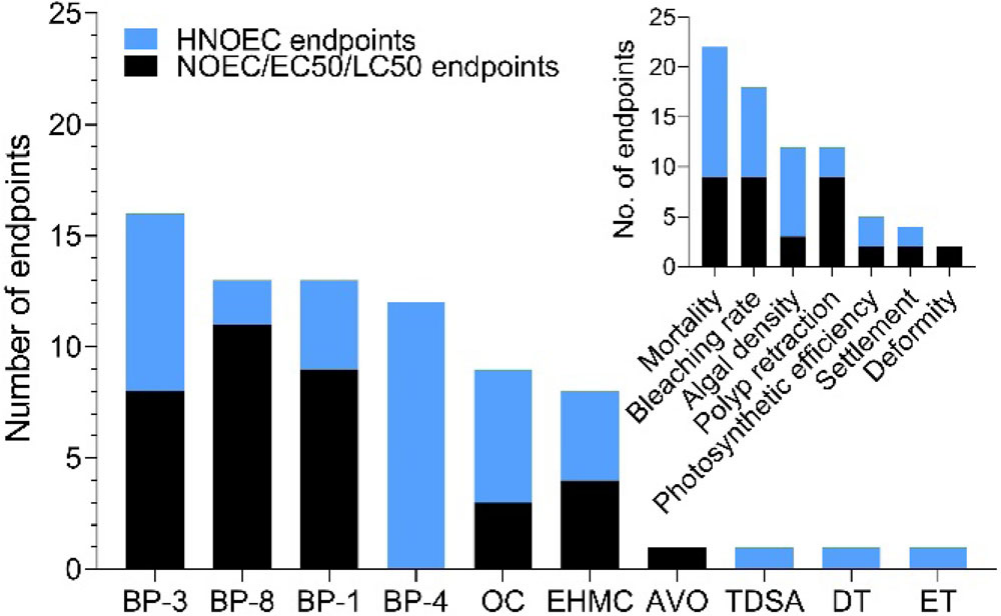
The proportion of coral no‐observed‐effect concentrations observed at the highest concentrations tested to the total number of endpoints presented in Figure 5 per ultraviolet (UV) filter. Data are presented by endpoint for all UV filters in the inset. The UV filter abbreviations are listed in Table 1. AVO = avobenzone; BP = benzophenone; DT = drometrizole trisilioxane; EC50 = median effect concentration; EHMC = ethylhexyl methoxycinnamate; ET = ethylhexyl triazone; HNOEC = highest NOEC; LC50 = median lethal concentration; NOEC = no‐observed‐effect concentration; OC = octocrylene; TDSA = terephthalylidene dicamphor sulfonic acid.

Many of the NOECs reported for coral actually correspond to the highest concentration tested (HNOEC), indicating that the true NOEC could be higher. Although not an ideal comparison, because of the limited amount of coral toxicity data currently available, we used this information to provide some insight into which UV filters and endpoints each coral species is most sensitive to. For all endpoints studied, exposure to BP‐4 resulted in an HNOEC (Figure 6). Also, DT, ET, and TDSA resulted in HNOECs; but because this only includes a single endpoint, it would be premature to comment on their relative potency in comparison to other reviewed UV filters. On the other hand, proportionally BP‐8 and BP‐1 resulted in the smallest fraction of HNOECs, 15 and 31%, respectively, providing further preliminary evidence that they might be more potent than other studied UV filters. In terms of the endpoints studied, only deformity had no HNOECs; but this was only included in the experiment by Downs et al. (2016; Figure 6, inset). Meanwhile, polyp retraction resulted in an HNOEC only 25% of the time, suggesting that this endpoint may be more sensitive than larval settlement or bleaching, which both resulted in an HNOEC for 50% of tests, although it is unclear what the ramifications of polyp retraction may be and the relationship with individual‐ or population‐level toxicity (Swain et al. 2015).

Overall conclusions regarding the toxicity of UV filters to corals are hampered by the limited amount of data, particularly given the variability in test designs. For risk identification it is important to identify the most sensitive species, life stage, and biological endpoint(s) for future testing in addition to assessing the relative sensitivity of new test species in relation to the commonly used standard test species.

## PRELIMINARY CORAL ENVIRONMENTAL RISK CHARACTERIZATION

A small subset of the exposure studies reviewed conducted a complementary preliminary environmental risk characterization for EHMC (Tsui et al. 2014; He et al. 2019a), benzophenones (Tsui et al. 2014, 2017; He et al. 2019b), and OC (He et al. 2019a). To conduct a risk assessment, it is critical to use high‐quality and reliable environmental exposure (e.g., predicted or monitoring) and toxicological (hazard) data (Leonards et al. 2013; Moermond et al. 2016). Often, risk is calculated deterministically, where an risk quotient is calculated by dividing exposure (e.g., predicted or measured) by a predicted‐no‐effect concentration (PNEC). When an risk quotient is ≥1, a risk is present. Regulatory environmental risk‐assessment (ERA) methodologies applied in the United States and Europe are generally aimed at the temperate freshwater environment (Fantke et al. 2018). Assumptions that are based on freshwater species are leveraged to the marine environment simply by increasing the assessment or uncertainty factor applied to toxicity data when no marine data are available (European Chemicals Agency 2008). However, these assessments are designed to protect the environment as a whole, rather than a particular species or taxon (e.g., coral). Currently, there is no established ERA approach specifically for coral (e.g., toxicity test recommendations, endpoints to include, appropriate assessment factors), but the studies reviewed mark an initial step toward how we can assess the impact of chemical stressors on coral.

To characterize UV filter exposure, Tsui et al. (2014) used a worst‐case scenario by taking the highest MEC from their monitoring data set (referred to as MEC_worst_). He et al. (2019a, 2019b) calculated a best‐ and worst‐case MEC using the minimum and maximum MECs collected in their studies and/or the previous data set reported by Tsui et al. (2014). Tsui et al. (2017) took a different approach to characterizing exposure: rather than using a surface‐water MEC, they used internal coral concentrations (MEC_internal_) derived from coral tissue they collected. Overall, a limited amount of monitoring data were considered or are even currently available for coral exposure characterization.

In terms of hazard characterization, a PNEC is calculated by dividing an LC(EC)50 (acute data) or NOEC/EC10 (chronic data) by an assessment factor (accounts for inter‐ and intraspecies variability; European Chemicals Agency 2008). Tsui et al. (2014) derived PNECs using data available in the literature (Danovaro et al. 2008; Downs et al. 2016), whereas He et al. (2019a, 2019b) calculated a PNEC based on each endpoint in their toxicity studies (rather than just the most sensitive). Tsui et al. (2017) took a nonstandard approach by multiplying existing toxicity data (Downs et al. 2016) by their field‐based coral bioaccumulation factors to get an EC(LC)50_internal_ and then applying an assessment factor. The resulting PNEC_internal_ was then comparable to their MEC_internal_. In all 4 studies, an assessment factor of 1000 was applied. This value was derived from outdated guidance from the European Commission (2003), which has now been replaced by guidance developed for the implementation of the Registration, Evaluation, Authorization and Restriction of Chemicals program (European Chemicals Agency 2008). By not following these guidelines, existing data were not considered that could impact the assessment factor applied and provide a more holistic marine UV filter risk characterization. According to this guidance, the PNEC should be based on the NOEC rather than the LOEC. Therefore, according to these established approaches, the PNECs derived by He et al. (2019a, 2019b) were not sufficiently conservative for preliminary risk‐screening purposes; however, this was likely overcome by applying such a large assessment factor to chronic empirical data. Beyond the assessment factor, there appear to be significant data reliability issues across toxicity studies used to derive PNECs (see previous section *Common methodological issues with characterizing UV filter hazard to coral*). This could be due to the lack of established guidance for the toxicological assessment of coral, but regardless, several aspects key to any toxicological study were missing (Moermond et al. 2016) including missing controls, lack of analytical confirmation of exposure concentrations, and missing mass to volume exposure concentrations (Danovaro et al. 2008; Downs et al. 2016). The reliability of the Downs et al. (2016) coral toxicity data has been questioned previously (Schaap and Slijkerman 2018), which was used for PNEC derivation by Tsui et al. (2014, 2017).

**Figure 7:**
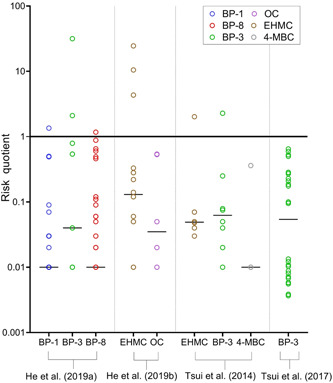
Summary of preliminary coral risk characterizations reported in the literature. A line is drawn at *y* = 1 to indicate when risk quotients exceed 1 (i.e., risk present), but note the multiple data quality issues with the underlying toxicity and exposure data identified in the review. The median risk quotient is identified per study per ultraviolet (UV) filter by the horizontal line. All risk quotients were retrieved from the supplemental data files provided in each study, with the exception of Tsui et al. (2017) where internal coral risk quotients were recalculated for the water column to make them comparable with the other data sets (see Supplemental Data for details). The UV filter abbreviations are listed in Table 1. BP = benzophenone; EHMC = ethylhexyl methoxycinnamate; 4‐MBC = 4‐methylbenzylidene camphor; OC = octocrylene.

In each of the reviewed studies, risk quotients were derived deterministically (i.e., MEC/PNEC), and the results are summarized in Figure 7. As mentioned, He et al. (2019a, 2019b) used Tsui et al. (2014) monitoring data; therefore, it is mainly the differences in PNEC that are driving the differences in risk quotients between the studies. Based on the median risk quotient, EHMC presents the greatest risk, followed closely by BP‐3 (Figure 7). All median risk quotients fall below 1, indicating that possible risks are minimal; however, the existence of particular study sites where risk quotients exceed 1 indicates that further investigations into the impacts UV filters may pose to coral are warranted (Figure 7).

We must acknowledge the limitations of the current data set in terms of methodological issues and completeness (as reviewed) for both monitoring and toxicological data, so much so that drawing any conclusions pertaining to risk of these compounds to coral would be premature. However, regardless of these concerns, a paucity of coral toxicological data restrains the ability of risk assessors and decision‐makers to derive reliable PNECs for coral. Alternatively to calculating a PNEC and an risk quotient, a margin of safety could be calculated by directly comparing a NOEC to the environmental concentration (NOEC/M[P]EC). This permits conclusions over the magnitude of the margin of safety (e.g., 1, 10, 100) rather than high versus low risk. Use of the margin of safety is also a reasonable alternative to the not‐fit‐for‐purpose large assessment factors (i.e., 1000) used in all of the coral risk assessments reviewed despite the risk assessments being species‐specific (not whole ecosystem) and using coral‐specific toxicity data. Others have circumvented this issue by deriving PNECs for closely related taxonomic groups, such as algae (Office of Protected Resources 2016; Schaap and Slijkerman 2018). To address this issue, reliable coral hazard data need to be generated (see Moermond et al. 2016). In addition, efforts should be made to investigate and justify the most sensitive and ecologically relevant coral test species and acute and chronic toxicological endpoints for hazard‐ and risk‐characterization purposes. Earlier analysis of toxicological data in the present review indicated that *S. caliendrum* along with larval settlement and adult coral polyp retraction endpoints were most sensitive (He et al. 2019a, 2019b), but data reliability issues related to these studies mean that their findings should be considered with caution.

A key point to consider when conducting ecotoxicological tests and subsequent ERA for coral is the paradigm in which we approach ERA. Priority is placed on higher levels of biological assemblage (individual < species < population < ecosystems) such that population‐level effects are of greater importance than effects on the individual. We know environmental responses to be variable, and as such, an effect observed on an individual may not translate into a change in the population. Therefore, careful consideration must be paid to endpoint selection such that it is relevant to an impact on the population rather than the individual. Research is needed to understand how the common coral toxicological endpoints translate into population‐level effects. In the context of the coral endpoints studied in response to UV filters thus far, mortality and larval settlement are population‐relevant endpoints, whereas the relevance to population‐level effects from larval deformity or polyp retraction endpoints is less clear. Those relating to the health of coral symbiosis (i.e., bleaching and loss of algae) and/or the impact to the symbiont itself (i.e., photosynthetic efficiency) are often used as precursors to coral mortality, although it is possible for corals to recover if the stressor is removed.

## CONCLUSION AND RECOMMENDATIONS

Organic UV filters do occur in the environment, but according to our analysis, there is limited evidence to suggest that their presence is causing significant harm to coral reefs. Although toxicity studies have observed effects, the majority of studies result in NOECs or LOECs, often observed at the highest concentrations tested. A few EC50 concentrations have been derived and, in the case of BP‐3, an LC50. The lowest EC50 across the UV filters was reported for BP‐3 (deformity, 49 µg/L), and the second lowest was for BP‐1 (larval settlement, 184.1 µg/L). These toxicological thresholds exceed 98 and 100%, respectively, of environmental concentrations observed thus far near coral reefs. But it should be noted that 2% of BP‐3 reef concentrations that exceed the EC50 are clear outliers, as reviewed. However, based on the current data set, it would be premature to conclude that environmental concentrations of UV filters do not adversely impact coral reefs. This is due to both extensive data gaps in terms of reliable and relevant environmental exposure and toxicity data. The major recommendations based on the findings from our analyses pertain to research needed on the following 3 aspects: environmental exposure and fate, toxicity testing, and risk assessment. These are summarized in Tables 4 and 5. Table 4 outlines our findings and provides recommendations for lower‐tier studies that should be addressed prior to conducting a preliminary risk assessment. If unacceptable risks are identified in the preliminary assessment, further refinement is needed, per the recommendations for higher‐tier studies presented in Table 5.

**Table 4:** Summary of findings and proposed recommendations for ultraviolet filter exposure studies and coral toxicity tests for the purpose of conducting a preliminary coral environmental risk assessment

Category	Finding or conclusion	Recommendations
Exposure	There are limited or no environmental exposure data available for many UV filters in coral habitats including both the water column and sediment. The majority of data available are only representative of a snapshot in time. Sample replication is also limited. Several data sets are lacking critical quality assurance/control components (e.g., matrix recovery and compensation strategy, limits of detection/quantification, lab and field blanks, inappropriate sampling containers and storage). Lack of filtering prior to extraction, difficult‐to‐relate results to dissolved fraction because many UV filters are highly hydrophobic.	Conduct marine environmental exposure modeling with limited refinements (e.g., excluding site‐specific dilution, wash‐off, etc.) to generate conservative predictions suitable for lower‐tier ERA. If small monitoring campaigns are conducted, ensure methods, quality assurance/control, and data reporting are complete and transparent; making use of supplementary files in publications should be encouraged. o Focus monitoring studies at various coral reefs sites (e.g. for the United States in Hawaii, Florida, and Puerto Rico).
Toxicity	Many studies currently available are of unknown relevance to population‐level endpoints, and all contain significant flaws that reduce their reliability and thus suitability for risk assessment. Existing endpoints may under‐ or overestimate toxicity thresholds. Most coral toxicity studies do not analytically verify their exposure concentrations, and those that do are of limited utility given their sampling design but do demonstrate significant losses of the parent compound. Many UV filters are relatively insoluble, and multiple solvents have been used in toxicity tests.	Develop a standard coral toxicity test that can generate reliable acute and chronic endpoints from dose–response relationships using appropriate test durations. Evaluate UV filters to identify the most suitable and sensitive population‐relevant biological endpoints (e.g., bleaching, growth, reproduction) and life stages for coral toxicity assessments (i.e., LC50, NOEC). Ensure use of appropriate solvents that do not enhance chemical uptake or potentially alter conditions of oxidative stress (e.g., MeOH, not DMSO). A comprehensive and robust test setup that ensures consistent toxic‐dose delivery. o A strategy to maintain UV filter concentrations throughout the exposure should be included, for example, a flow‐through design. o An analytical sampling plan suitable to calculate robust time‐weighted exposure concentrations to derive reliable toxicity thresholds.
Risk assessment	A limited number of coral ERAs have been conducted but utilize generalized (large) assessment factors and measured environmental concentrations and coral toxicity data that are not robust. Current research suggests risks of UV filters to coral could be present, and further investigation and refinement of the risk assessment is needed. Reliable and relevant toxicity and exposure data are currently lacking but essential for well‐informed decision‐making/risk mitigation.	Environmental monitoring and toxicity testing recommendations should be implemented, resulting in a robust data set for preliminary deterministic ERA calculation, which would allow for a more accurate risk determination of UV filters to corals. Develop a tiered coral‐specific ERA framework. The framework should incorporate existing data where possible, including leveraging data for other species with suitable assessment factors. If using coral data, apply the margin‐of‐safety approach rather than assessment factor–driven risk quotients. The lower tiers of the assessment should be deterministic rather than probabilistic because that requires higher‐tier modeled exposure data.

ERA = environmental risk assessment; LC50 = median lethal concentration; NOEC = no‐observed‐effect concentration; UV = ultraviolet.

**Table 5:** Summary of findings and proposed recommendations for ultraviolet filter exposure and fate studies and coral toxicity tests for the purpose of refining a coral environmental risk assessment^a^

Category	Finding or conclusion	Recommendations
Exposure and fate	Concentrations of UV filters are highly variable through space and time, and there are limited data at environmentally relevant depths (i.e., at coral reef depth or in the surface microlayer where coral larvae live).	Conduct environmental monitoring studies at varying depths in the water column at various coral reef sites with repeated sampling at the same sites over various timescales (tidal cycle, day, week, month, and season). Include hydrological, water quality, and other potential covariable descriptions at sites.
	There is some evidence that UV filter concentrations are correlated with recreational activity, although it is limited and study‐dependent, with some studies showing high UV filter concentrations in areas of no or limited recreational activity. There are multiple sources of UV filters in marine environments including plastics, textiles, paints, wastewater effluent, and overland runoff.	Conduct comprehensive monitoring programs at sites that differ in anthropogenic influence (i.e., WWTP, recreation, urban versus rural land runoff, or remote sites). Include potential chemical tracers for source apportionment studies (e.g., sucralose as a wastewater tracer). Conduct follow‐on UV filter source apportionment studies. These could include coupling isotopic analysis and leaching studies from common household products to determine the potential sources, for example, from plastics or textiles.
	Many UV filters are highly lipophilic and likely to partition to particles in seawater or organisms. The distribution between the particulate and dissolved phase is understudied and has implications for exposure modeling and uptake by corals.	Assess the partitioning of UV filters between the dissolved and particulate phases (using filtration techniques) in seawater from laboratory and environmental settings.
	Multiple studies have found loss of UV filters in toxicity test exposure solutions and variability in field concentrations during the course of the day.	Study the persistence, degradation (e.g., by photolysis), and metabolism of a variety of UV filters in laboratory studies under conditions pertinent for tropical coral reefs (e.g., spectral quantity and quality, interaction with dissolved organic matter).
	Corals were shown to bioaccumulate some UV filters; but the rate of uptake is unknown, and limited data exist only for a few UV filters in 2 studies.	Conduct laboratory or in situ coral transplant bioaccumulation studies, determine uptake and depuration rates for UV filters in adult coral fragments, and identify metabolites generated.
Toxicity	There are limited data on the acute and chronic toxicity of UV filters to intact hard coral species in both adult and larval life stages. Impacts of UV filters differ with species and life stage and are not consistent (i.e., some UV filters are more toxic to larvae, whereas others are more toxic to adult fragments). Obtaining sufficient adult hard coral fragments and coral larvae for exposure experiments is challenging given permitting requirements, large numbers required for toxicity tests and for larvae the limited potential sampling window of time for collection.	Standard tests should be developed using at least 2 coral species and multiple life stages and require toxicity tests for each compound to be conducted in multiple coral species using adult fragments and larvae. Assess species for development of a standard cnidarian test species for the rapid screening of UV filter toxicity (e.g., the symbiotic tropical anemone [*Aiptasia* spp.], the symbiotic soft coral [*Xenia* spp.], or the fast‐growing symbiotic hard coral [*Galaxea* sp.]).
Risk assessment	Deterministic lower‐tier coral environmental risk assessment oversimplifies coral UV filter exposures and assumes the worst‐case exposure scenario.	A probabilistic approach could be a suitable strategy to refine coral exposure. This would require higher‐tier marine exposure modeling but would provide a more realistic indication of exposure and risk probability.
	A holistic multistressor risk assessment could be important for ensuring effective management and conservation of reef environments given that; Multiple UV filters are present in seawater and sediment near coral reefs and in coral tissues. Exposure of corals to chemical contaminants and costressors (e.g., temperature) results in increased toxicity, although there is very limited evidence for this with UV filters. The relative risk of known coral stressors can be ranked against risks from UV filters or other organic pollutants.	An eco‐epidemiological approach could be a useful strategy for evaluating combinations of physical, chemical, and environmental conditions through time to identify dominant stressors.^b^ The approach can help optimize management strategies, reveal potential causal relationships, or target site selection for monitoring. This approach is also consistent with the decision framework presented for the persistence and resilience of coral reefs.^c^ Based on the outcome of the eco‐epidemiological approach: o Toxicity studies could be conducted to assess the impact of multiple UV filters to corals. o Studies could be conducted to assess the synergistic or additive effect of costressors (e.g., temperature changes) on the toxicity of UV filters to corals.

^a^ These studies should be considered after lower‐tier recommendations have been filled (Table 4) and a potential risk is demonstrated.

^b^ Posthuma et al. (2016), Kapo et al. (2014).

^c^ National Academies of Sciences, Engineering and Medicine (2019).

UV = ultraviolet; WWTP = wastewater‐treatment plant.

The first aspect, addressed in Table 4, concerns UV filter environmental exposure. Although representative monitoring studies with adequate quality control are useful for establishing UV filter exposure in coral habitats, efforts to model these concentrations are encouraged to predict concentrations that can be used in a preliminary risk assessment. If needed, these predicted data can also inform higher‐tier targeted monitoring programs (Table 5). These higher‐tier monitoring studies should adequately capture temporal and spatial fluctuations, particularly in relation to where coral is found within the environment. This includes studying depth distribution and characterization of the surface microlayer, both important aspects for understanding the true exposure of coral through its various life stages. Temporal variability also needs to be better understood; this could be achieved through the study of exposure on various timescales (e.g., tidal, daily, monthly, seasonal). Also important is the characterization and selection of a variety of sites that include varying levels of anthropogenic (e.g., wastewater effluent impacted, recreational, pristine) and hydrodynamic (e.g., low and high tidal influence, sheltered) influence. Ideally, additional chemical tracers could be used to help determine potential source inputs (e.g., sucralose for wastewater effluent; Oppenheimer et al. 2011). This information can also be used to gain an understanding of UV filter source apportionment. Greater effort should also be put into describing both the particulate and dissolved phases of water samples collected because this could enhance the understanding of exposure pathways of these particularly hydrophobic chemicals. Together, this UV filter fate and occurrence information can be fed into spatially explicit coastal exposure modeling activities, which should be explored as a complementary approach for assessing coral exposure.

In addition to field monitoring, a series of laboratory‐based fate studies could provide a wealth of information on the partitioning, persistence, and degradation of UV filters in simulated reef conditions (e.g., salinity, temperature, and light). For example, incubation experiments of UV filters in seawater, with and without simulated “natural” UV light conditions and dissolved organic carbon, could be conducted to assess stability, partitioning, and degradation in seawater. Laboratory fate studies with coral also need to be conducted to determine the uptake, bioaccumulation, and metabolism of UV filters in a variety of coral species. Because there is evidence to suggest that coral UV filter metabolism occurs (Tsui et al. 2017; Stien et al. 2019), analytical detection of metabolites when conducting analyses of coral tissue and/or exposure media would be useful to determine the extent of UV filter metabolism and its role in toxicity. More coral tissue data from the field are also needed and can provide complementary information to laboratory studies to aid in the assessment of exposure, uptake, and bioaccumulation (i.e., partitioning between the dissolved and particulate phases). Characterization of coral dietary exposure (e.g., zooplankton) is also required for this purpose. Finally, a complementary coral health assessment should be completed with any environmental samples near reefs to serve as a reference when discussing exposure. This information could also form a key data set to use within an eco‐epidemiological approach (Table 5).

The second major research aspect is a need to conduct more controlled laboratory toxicity tests with justified toxicity endpoints and robust study designs. Based on the studies reviewed, a standardized protocol for coral toxicity testing would be of great value because there is currently no standard toxicity test for corals (Table 4). Research should be conducted to identify sensitive and appropriate coral model species or even surrogate species (e.g., anemones) for toxicity studies using multiple life stages (i.e., adults and larvae). Selected species should be easily cultured and maintained in a laboratory setting for weeks (for chronic tests), provide adequate material for regular testing, and result in minimal variation between tests and species with respect to negative and positive control responses. It is imperative that these studies follow good ecological testing practice to generate reliable data suitable for risk assessment. Acute and chronic tests need to be differentiated and have endpoints included that are suitable for ERA. Acute (<96 h) exposures should focus on lethality and derive an LC50. These acute toxicity tests would ideally include 2 or more species of stony coral and expose adult coral fragments generated from multiple, genetically different individual “parents.” Higher‐tier chronic exposures should include a suite of sublethal endpoints, preferably those related to growth and reproduction, although research is needed to determine additional suitable and sensitive endpoints (e.g., bleaching, photosynthetic activity, and chlorophyll content); but the goal should be to derive an EC10/NOEC suitable for ERA (e.g., European Chemicals Agency, US Environmental Protection Agency). Because it is still unclear as to the most sensitive life stage to use for a coral, the acute and chronic toxicity studies outlined above could also be conducted in the early life stages (larvae/planulae) of 2 or more coral species including additional developmental endpoints and assessment of life‐stage sensitivities. Assessing single‐compound toxicity should be the priority for all UV filters. As the body of robust toxicity data develops, investigations into UV filter mixtures, major metabolites, or degradation products could be considered.

This brings us to the third major research aspect, conducting a risk assessment. There is a need to develop a targeted and suitably protective coral ERA framework. This framework could be based on existing marine ERA frameworks (e.g., European Chemicals Agency 2008) and tiered, leveraging data from standard species into the assessment while also addressing coral‐specific concerns, thereby enabling researchers and decision‐makers to put environmental exposures and coral hazard in the context of risk for corals and marine ecosystems more generally. A systematic framework to determine the risk of UV filters will also help to prioritize risk in terms of other coral contaminants, thereby directing resources to where they will have greatest impact. Ultimately, the environmental risk of UV filters may be realized as a costressor with other factors, such as ocean temperature. In addition, the relative risks of UV filters compared to costressors may be evaluated. There is a potential to investigate these interactions through the eco‐epidemiological approach (Table 5), which could provide a mechanism for conducting a holistic ERA for coral. This would allow regulators, policymakers, and scientists to optimize conservation and management activities while enabling the identification of priority stressors that should be most urgently addressed.

There is currently limited evidence to suggest that corals are adversely impacted by environmental exposure to UV filters; however, these major data gaps immediately need to be addressed with high‐quality monitoring, fate, and toxicity studies. Together these studies can be used to appropriately quantify the risk of coral to UV filters, thus enabling assessors to make informed, evidence‐based decisions that will truly be of benefit for coral health.

## Supplemental Data

The Supplemental Data are available on the Wiley Online Library at https://doi.org/10.1002/etc.4948.

## Supporting information

This article contains online‐only Supplemental Data.

Supporting information.

## Data Availability

All data are available within the article and Supplemental Data.

## References

[etc4948-bib-0001] Amid C , Olstedt M , Gunnarsson JS , Le Lan H , Tran Thi Minh H , Van den Brink PJ , Hellström M , Tedengren M . 2018. Additive effects of the herbicide glyphosate and elevated temperature on the branched coral *Acropora formosa* in Nha Trang, Vietnam. Environ Sci and Pollut Res 25:13360–13372. 10.1007/s11356-016-8320-7 PMC5978828 28111719

[etc4948-bib-0002] Antweiler RC . 2015. Evaluation of statistical treatments of left‐censored environmental data using coincident uncensored data sets: II. Group comparisons. Environ Sci Technol 49:13439–13446. 26490190 10.1021/acs.est.5b02385

[etc4948-bib-0003] Apel C , Tang J , Ebinghaus R . 2018. Environmental occurrence and distribution of organic UV stabilizers and UV filters in the sediment of Chinese Bohai and Yellow Seas. Environ Pollut 235:85–94. 29275272 10.1016/j.envpol.2017.12.051

[etc4948-bib-0004] Bargar TA , Alvarez DA , Garrison VH . 2015. Synthetic ultraviolet light filtering chemical contamination of coastal waters of Virgin Islands National Park, St. John, U.S. Virgin Islands. Mar Pollut Bull 101:193–199. 26581812 10.1016/j.marpolbul.2015.10.077

[etc4948-bib-0005] Bejarano A . 2018. Critical review and analysis of aquatic toxicity data on oil spill dispersants. Environ Toxicol Chem 37:2989–3001. 30125977 10.1002/etc.4254

[etc4948-bib-0104] Benedé JL , Chisvert A , Salvador A , Sánchez‐Quiles D , Tovar‐Sánchez A . 2014. Determination of UV filters in both soluble and particulate fractions of seawaters by dispersive liquid‐liquid microextraction followed by gas chromatography‐mass spectrometry. Anal Chim Acta 812:50–58. 10.1016/j.aca.2013.12.033 24491764

[etc4948-bib-0006] Boix C , Ibáñez M , Sancho JV , Rambla J , Aranda JL , Ballester S , Hernández F . 2015. Fast determination of 40 drugs in water using large volume direct injection liquid chromatography‐tandem mass spectrometry. Talanta 131:719–727. 25281164 10.1016/j.talanta.2014.08.005

[etc4948-bib-0007] Brown BE , Dunne RP , Goodson MS , Douglas AE . 2000. Bleaching patterns in reef corals. Nature 404:142–143. 10.1038/35004657 10724156

[etc4948-bib-0008] Cadena‐Aizaga ML , Montesdeoca‐Esponda S , Torres‐Padrón ME , Sosa‐Ferrera Z , Santana‐Rodríguez JJ . 2020. Organic UV filters in marine environments: An update of analytical methodologies, occurrence and distribution. Trends Environ Anal Chem 25:e00079.

[etc4948-bib-0009] Chisvert A , Salvador A . 2007. UV filters in sunscreens and other cosmetics: Regulatory aspects and analytical methods. In Salvador A , Chisvert A , eds, Analysis of Cosmetic Products. Elsevier, Oxford, UK, pp 83–120.

[etc4948-bib-0010] Danovaro R , Bongiorni L , Corinaldesi C , Giovannelli D , Damiani E , Astolfi P , Greci L , Pusceddu A . 2008. Sunscreens cause coral bleaching by promoting viral infections. Environ Health Perspect 116:441–447. 18414624 10.1289/ehp.10966 PMC2291018

[etc4948-bib-0011] de Goeij JM , van Duyl FC . 2007. Coral cavities are sinks of dissolved organic carbon (DOC). Limnol Oceanogr 52:2608–2617.

[etc4948-bib-0012] Downs CA , Fauth JE , Downs VD , Ostrander GK . 2010. In vitro cell‐toxicity screening as an alternative animal model for coral toxicology: Effects of heat stress, sulfide, rotenone, cyanide, and cuprous oxide on cell viability and mitochondrial function. Ecotoxicology 19:171–184. 19757033 10.1007/s10646-009-0403-5

[etc4948-bib-0013] Downs CA , Kramarsky‐Winter E , Segal R , Fauth J , Knutson S , Bronstein O , Ciner FR , Jeger R , Lichtenfield Y , Woodley CM , Pennington P , Cadenas K , Kushmaro A , Loya Y . 2016. Toxicopathological effects of the sunscreen UV filter, oxybenzone (benzophenone‐3), on coral planulae and cultured primary cells and its environmental contamination in Hawaii and the U.S. Virgin Islands. Arch Environ Contam Toxicol 70:265–288. 26487337 10.1007/s00244-015-0227-7

[etc4948-bib-0014] Duprey NN , Yasuhara M , Baker DM . 2016. Reefs of tomorrow: Eutrophication reduces coral biodiversity in an urbanized landscape. Glob Chang Biol 22:3550–3565. 27414018 10.1111/gcb.13432

[etc4948-bib-0015] European Chemicals Agency . 2008. Chapter R.10: Characterisation of dose [concentration]–response for environment. In *Guidance on Information Requirements and Chemical Safety Assessment*. Helsinki, Finland.

[etc4948-bib-0016] European Chemicals Agency . 2020. Registered substances. Helsinki, Finland. [cited 2020 June 3]. Available from: https://echa.europa.eu/information-on-chemicals/registered-substances

[etc4948-bib-0017] European Commission . 2003. Technical guidance document on risk assessment in support of commission directive 93/67/EEC on risk assessment for new notified substances, commission regulation (EC) No. 1488/94 on risk assessment for existing substances, and directive 98/8/EC of the European Parliament and of the Council Concerning the Placing of Biocidal Products on the Market. Part II. EUR 20418 EN/2. Joint Research Centre, Ispra, Italy.

[etc4948-bib-0018] Fantke P , Aurisano N , Bare J , Backhaus T , Bulle C , Chapman PM , De Zwart D , Dwyer R , Ernstoff A , Golsteijn L , Holmquist H , Jolliet O , McKone TE , Owsianiak M , Peijnenburg W , Posthuma L , Roos S , Saouter E , Schowanek D , van Straalen NM , Vijver MG , Hauschild M . 2018. Toward harmonizing ecotoxicity characterization in life cycle impact assessment. Environ Toxicol Chem 37:2955–2971. 30178491 10.1002/etc.4261 PMC7372721

[etc4948-bib-0019] Fel JP , Lacherez C , Bensetra A , Mezzache S , Béraud E , Léonard M , Allemand D , Ferrier‐Pagès C . 2019. Photochemical response of the scleractinian coral *Stylophora pistillata* to some sunscreen ingredients. Coral Reefs 38:109–122.

[etc4948-bib-0020] Fent K , Zenker A , Rapp M . 2010. Widespread occurrence of estrogenic UV‐filters in aquatic ecosystems in Switzerland. Environ Pollut 158:1817–1824. 20004505 10.1016/j.envpol.2009.11.005

[etc4948-bib-0021] Ferrier‐Pagès C , Witting J , Tambutté E , Sebens KP . 2003. Effect of natural zooplankton feeding on the tissue and skeletal growth of the scleractinian coral *Stylophora pistillata* . Coral Reefs 22:229–240.

[etc4948-bib-0022] Forbes VE , Galic N , Schmolke A , Vavra J , Pastorok R , Thorbek P . 2016. Assessing the risks of pesticides to threatened and endangered species using population modeling: A critical review and recommendations for future work. Environ Toxicol Chem 35:1904–1913. 27037541 10.1002/etc.3440

[etc4948-bib-0023] Furlong ET , Kanagy CJ , Kanagy LK , Coffey LJ , Burkhardt MR . 2014. Determination of human‐use pharmaceuticals in filtered water by direct aqueous injection‐high‐performance liquid chromatography/tandem mass spectrometry: U.S. Geological Survey techniques and methods. In *Section B, Methods of the National Water Quality Laboratory. Book 5, Laboratory Analysis*. US Geological Survey, Reston, VA.

[etc4948-bib-0024] Giokas DL , Salvador A , Chisvert A . 2007. UV filters: From sunscreens to human body and the environment. Trends Anal Chem 26:360–374.

[etc4948-bib-0025] Goksøyr A , Tollefsen KE , Grung M , Løken K , Lie E , Zenker A , Fent K , Schlabach M , Huber S . 2009. Balsa raft crossing the Pacific finds low contaminant levels. Environ Sci Technol 43:4783–4790. 19673265 10.1021/es900154h

[etc4948-bib-0026] Harris CA , Scott AP , Johnson AC , Panter GH , Sheahan D , Roberts M , Sumpter JP . 2014. Principles of sound ecotoxicology. Envriron Sci Technol 48:3100–3111. 10.1021/es4047507 24512103

[etc4948-bib-0027] Hata H , Kudo S , Yamano H , Kurano N , Kayanne H . 2002. Organic carbon flux in Shiraho coral reef (Ishigaki Island, Japan). Mar Ecol Prog Ser 232:129–140.

[etc4948-bib-0028] He T , Tsui MMP , Tan CJ , Ma CY , Yiu SKF , Wang LH , Chen TH , Fan TY , Lam PKS , Murphy MB . 2019a. Toxicological effects of two organic ultraviolet filters and a related commercial sunscreen product in adult corals. Environ Pollut 245:462–471. 30458376 10.1016/j.envpol.2018.11.029

[etc4948-bib-0029] He T , Tsui MMP , Tan CJ , Ng KY , Guo FW , Wang LH , Chen TH , Fan TY , Lam PKS , Murphy MB . 2019b. Comparative toxicities of four benzophenone ultraviolet filters to two life stages of two coral species. Sci Total Environ 651:2391–2399. 30336428 10.1016/j.scitotenv.2018.10.148

[etc4948-bib-0030] Hoegh‐Guldberg O , Poloczanska ES , Skirving W , Dove S . 2017. Coral reef ecosystems under climate change and ocean acidification. Front Mar Sci 4:158.

[etc4948-bib-0031] Horricks RA , Tabin SK , Edwards JJ , Lumsden JS , Marancik DP . 2019. Organic ultraviolet filters in nearshore waters and in the invasive lionfish (*Pterois volitans*) in Grenada, West Indies. PLoS One 14:e0220280. 31339964 10.1371/journal.pone.0220280 PMC6655699

[etc4948-bib-0032] Hughes TP , Anderson KD , Connolly SR , Heron SF , Kerry JT , Lough JM , Baird AH , Baum JK , Berumen ML , Bridge TC , Claar DC , Eakin CM , Gilmour JP , Graham NAJ , Harrison H , Hobbs JPA , Hoey AS , Hoogenboom M , Lowe RJ , McCulloch MT , Pandolfi JM , Pratchett M , Schoepf V , Tordda G , Wilson SK . 2018. Spatial and temporal patterns of mass bleaching of corals in the Anthropocene. Science 359:80–83. 29302011 10.1126/science.aan8048

[etc4948-bib-0033] Imbs AB . 2013. Fatty acids and other lipids of corals: Composition, distribution and bio‐synthesis. J Mar Biol 39:153–168.

[etc4948-bib-0034] Imhof HK , Sigl R , Brauer E , Feyl S , Giesemann P , Klink S , Leupolz K , Löder MGJ , Löschel LA , Missun J , Muszynski S , Ramsperger AFRM , Schrank I , Speck S , Steibl S , Trotter B , Winter I , Laforsch C . 2017. Spatial and temporal variation of macro‐, meso‐ and microplastic abundance on a remote coral island of the Maldives, Indian Ocean. Mar Pollut Bull 116:340–347. 28109654 10.1016/j.marpolbul.2017.01.010

[etc4948-bib-0035] Kais B , Schneider KE , Keiter S , Henn K , Ackermann C , Braunbeck T . 2013. DMSP modifies the permeability of the zebrafish (*Danio rerio*) chorion—Implications for the fish embryo test (FET). Aquat Toxicol 140:229–238. 23831690 10.1016/j.aquatox.2013.05.022

[etc4948-bib-0036] Kapo KE , Holmes CM , Dyer SD , de Zwart D , Posthuma L . 2014. Developing a foundation for eco‐epidemiological assessment of aquatic ecological status over large geographic regions utilizing existing data resources and models. Environ Toxicol Chem 33:1665–1677. 24648183 10.1002/etc.2557

[etc4948-bib-0037] Key West City Commission . 2019. Details of ordinance file #18‐3253. Key West, FL, USA. [cited 2020 April 30]. Available from: https://keywest.legistar.com/LegislationDetail.aspx?ID=3763135&GUID=EFF5%20D76E-F043-4AFF-A898-42EB20A25953

[etc4948-bib-0038] Kim S , Choi K . 2014. Occurrences, toxicities, and ecological risks of benzophenone‐3, a common component of organic sunscreen products: A mini‐review. Environ Int 70:143–157. 24934855 10.1016/j.envint.2014.05.015

[etc4948-bib-0039] Kroon FJ , Berry KLE , Brinkman DL , Kookana R , Leusch FDL , Melvin SD , Neale PA , Negri AP , Puotinen M , Tsang JJ , van de Merwe JP , Williams M . 2020. Sources, presence and potential effects of contaminants of emerging concern in the marine environments of the Great Barrier Reef and Torres Strait, Australia. Sci Total Environ 719:135140. 31859059 10.1016/j.scitotenv.2019.135140

[etc4948-bib-0040] Kuanui P , Chavanich S , Viyakarn V , Omori M , Fujita T , Lin C . 2020. Effect of light intensity on survival and photosynthetic efficiency of cultured corals of different ages. Estuar Coast Shelf Sci 235:106515.

[etc4948-bib-0041] Kung TA , Lee SH , Yang TC , Wang WH . 2018. Survey of selected personal care products in surface water of coral reefs in Kenting National Park, Taiwan. Sci Total Environ 635:1302–1307. 29710583 10.1016/j.scitotenv.2018.04.115

[etc4948-bib-0042] Labille J , Slomberg D , Catalano R , Robert S , Apers‐Tremelo ML , Boudenne JL , Manasfi T , Radakovitch O . 2020. Assessing UV filter inputs into beach waters during recreational activity: A field study of three French Mediterranean beaches from consumer survey to water analysis. Sci Total Environ 706:136010. 31855634 10.1016/j.scitotenv.2019.136010

[etc4948-bib-0043] Leonards P , Eadsforth C , Schowanek D . 2013. Monitoring base surfactants—A database specifically for storage of environmental data on surfactants in Europe. Tenside Surfactants Detergents 50:325–331.

[etc4948-bib-0044] McCoshum SM , Schlarb AM , Baum KA . 2016. Direct and indirect effects of sunscreen exposure for reef biota. Hydrobiologia 776:139–146.

[etc4948-bib-0045] Ministries of The Netherlands . 2020. Plan for land & water: Nature and environmental policy plan Caribbean Netherlands. The Hague, The Netherlands. [cited 2020 April 30]. Available from: https://english.rijksdienstcn.com/documents/publications/ezk/nature-and-environment-policy-plan/nature-and-environment-policy-plan/index

[etc4948-bib-0046] Mitchelmore CL , He K , Gonsior M , Hain E , Heyes A , Clark C , Younger R , Schmitt‐Kopplin P , Feerick A , Conway A , Blaney L . 2019. Occurrence and distribution of UV‐filters and other anthropogenic contaminants in coastal surface water, sediment, and coral tissue from Hawaii. Sci Total Environ 670:398–410. 30904653 10.1016/j.scitotenv.2019.03.034

[etc4948-bib-0047] Moberg F , Folke C . 1999. Ecological goods and services of coral reef ecosystems. Ecol Econ 29:215–233.

[etc4948-bib-0048] Moermond C , Beasley A , Breton R , Junghans M , Laskowski R , Solomon K , Zahner H . 2017. Assessing the reliability of ecotoxicological studies: An overview of current needs and approaches. Integr Environ Assess Manag 13:640–651. 27869364 10.1002/ieam.1870

[etc4948-bib-0049] Moermond CTA , Kase R , Korkaric M , Ågerstrand M . 2016. CRED: Criteria for reporting and evaluating ecotoxicity data. Environ Toxicol Chem 35:1297–1309. 26399705 10.1002/etc.3259

[etc4948-bib-0050] National Academies of Sciences, Engineering, and Medicine . 2017. Using 21st Century Science to Improve Risk‐Related Evaluations. National Academies, Washington, DC. 28267305

[etc4948-bib-0051] National Academies of Sciences, Engineering, and Medicine . 2019. A Decision Framework for Interventions to Increase the Persistence and Resilience of Coral Reefs. National Academies, Washington, DC.

[etc4948-bib-0052] Negri AP , Hoogenboom MO . 2011. Water contamination reduces the tolerance of coral larvae to thermal stress. PLoS One 6:e0019703. 10.1371/journal.pone.0019703 PMC3092768 21589934

[etc4948-bib-0053] Nelson CE , Alldredge AL , McCliment EA , Amaral‐Zettler LA , Carlson CA . 2011. Depleted dissolved organic carbon and distinct bacterial communities in the water column of a rapid‐flushing coral reef system. ISME J 5:1374–1387. 21390080 10.1038/ismej.2011.12 PMC3146267

[etc4948-bib-0054] Office of Protected Resources . 2016. Biological opinion on EPA Pesticides General Permit for Discharge of Pollutants into U.S. Waters. FPR‐2016‐9154. National Marine Fisheries Service, Silver Spring, MD, USA.

[etc4948-bib-0055] O'Malley E , McLachlan MS , O'Brien JW , Verhagen R , Mueller JF . 2021. The presence of selected UV filters in a freshwater recreational reservoir and fate in controlled experiments. Sci Total Environ 751:142373. 10.1016/j.scitotenv.2020.142373 33254898

[etc4948-bib-0056] Oppenheimer J , Eaton A , Badruzzaman M , Haghani AW , Jacangelo JG . 2011. Occurrence and suitability of sucralose as an indicator compound of wastewater loading to surface waters in urbanized regions. Water Res 45:4019–4027. 21665241 10.1016/j.watres.2011.05.014

[etc4948-bib-0057] Organisation for Economic Co‐operation and Development . 1992. Test No. 210: Fish, early‐life stage toxicity test. *OECD Guidelines for the Testing of Chemicals*. Paris, France.

[etc4948-bib-0058] Organisation for Economic Co‐operation and Development . 2011. Test No. 201: Freshwater alga and cyanobacteria, growth inhibition test. *OECD Guidelines for the Testing of Chemicals*. Paris, France.

[etc4948-bib-0059] Organisation for Economic Co‐operation and Development . 2012. Test No. 211: *Daphnia magna* reproduction test. *OECD Guidelines for the Testing of Chemicals*. Paris, France.

[etc4948-bib-0060] Organisation for Economic Co‐operation and Development . 2013. Test No. 236: Fish embryo acute toxicity (FET) test. *OECD Guidelines for the Testing of Chemicals*. Paris, France.

[etc4948-bib-0061] Organisation for Economic Co‐operation and Development . 2019a. Test No. 203: Fish, acute toxicity test. *OECD Guidelines for the Testing of Chemicals*. Paris, France.

[etc4948-bib-0062] Organisation for Economic Co‐operation and Development . 2019b. Guidance document on aquatic toxicity testing of difficult substances and mixtures. OECD Series on Testing and Assessment, No. 23. ENV/JM/MONO(2000)6/REV1. Paris, France.

[etc4948-bib-0063] Owen R , Mitchelmore C , Woodley C , Trapido‐Rosenthal H , Galloway T , Depledge M , Readman J , Buxton L , Sarkis S , Jones R , Knap A . 2005. A common sense approach for confronting coral reef decline associated with human activities. Mar Pollut Bull 51:481–485. 16199062 10.1016/j.marpolbul.2005.08.011

[etc4948-bib-0064] Pawlowski S , Lanzinger AC , Dolich T , Füßl S , Salinas ER , Zok S , Weiss B , Hefner N , Van Sloun P , Hombeck H , Klingelmann E , Petersen‐Thierry M . 2019. Evaluation of the bioaccumulation of octocrylene after dietary and aqueous exposure. Sci Total Environ 672:669–679. 30974358 10.1016/j.scitotenv.2019.03.237

[etc4948-bib-0065] Posthuma L , Dyer SD , de Zwart D , Kapo K , Holmes CM , Burton GA . 2016. Eco‐epidemiology of aquatic ecosystems: Separating chemicals from multiple stressors. Sci Total Environ 573:1303–1319. 27519323 10.1016/j.scitotenv.2016.06.242

[etc4948-bib-0066] Raffa RB , Pergolizzi JV , Taylor R , Kitzen JM . 2019. Sunscreen bans: Coral reefs and skin cancer. J Clin Pharm Ther 44:134–139. 30484882 10.1111/jcpt.12778

[etc4948-bib-0067] Ramos S , Homem V , Alves A , Santos L . 2015. Advances in analytical methods and occurrence of organic UV‐filters in the environment—A review. Sci Total Environ 526:278–311. 25965372 10.1016/j.scitotenv.2015.04.055

[etc4948-bib-0068] Remengesau TE Jr . 2018. Subject: Signing statement SB no. 10‐135, SD1, HD1 (the Responsible Tourism Education Act of 2018). [cited 2020 April 30]. Available from: http://extwprlegs1.fao.org/docs/pdf/pau181409.pdf

[etc4948-bib-0069] Schaap I , Slijkerman DME . 2018. An environmental risk assessment of three organic UV‐filters at Lac Bay, Bonaire, southern Caribbean. Mar Pollut Bull 135:490–495. 30301064 10.1016/j.marpolbul.2018.07.054

[etc4948-bib-0070] Schneider SL , Lim HW . 2019. Review of environmental effects of oxybenzone and other sunscreen active ingredients. J Am Acad Dermatol 80:266–271. 29981751 10.1016/j.jaad.2018.06.033

[etc4948-bib-0071] Schutter M , Crocker J , Paijmans A , Janse M , Osinga R , Verreth AJ , Wijffels RH . 2010. The effect of different flow regimes on the growth and metabolic rates of the scleractinian coral *Galaxea fascicularis* . Coral Reefs 29:737–748.

[etc4948-bib-0072] Schutter M , van der Ven RS , Janse M , Verreth JAJ , Wijffels RH , Osinga R . 2012. Light intensity, photoperiod duration, daily light flux and coral growth of *Galaxea fascicularis* in an aquarium setting: A matter of photons? J Mar Biol Assoc UK 92:703–712.

[etc4948-bib-0073] Sebens KP , Helmuth B , Carrington E , Agius B . 2003. Effects of water flow on growth and energetics of the scleractinian coral *Agaricia tenuifolia* in Belize. Coral Reefs 22:35–47.

[etc4948-bib-0074] Sirois J . 2019. Examine all available evidence before making decisions on sunscreen ingredient bans. Sci Total Environ 674:211–212. 31004897 10.1016/j.scitotenv.2019.04.137

[etc4948-bib-0075] Smith LW , Birkeland C . 2007. Effects of intermittent flow and irradiance level on back reef *Porites* corals at elevated seawater temperatures. J Exp Mar Biol Ecol 341:282–294.

[etc4948-bib-0076] Sobanska M , Scholz S , Nyman A‐M , Cesnaitis R , Gutierrez Alonso S , Klüver N , Kühne R , Tyle H , de Knecht J , Dang Z , Lundbergh I , Carlon C , De Coen W . 2018. Applicability of the fish embryo acute toxicity (FET) test (OECD 236) in the regulatory context of Registration, Evaluation, Authorisation, and Restriction of Chemicals (REACH). Environ Toxicol Chem 37:657–670. 29226368 10.1002/etc.4055

[etc4948-bib-0077] Spalding MD , Brown BE . 2015. Warm‐water coral reefs and climate change. Science 350:769–771. 26564846 10.1126/science.aad0349

[etc4948-bib-0078] State of Hawaii Senate . 2018. Details of Bill S.B. No. 2571, S.D. 2, H.D. 2, C.D. 1. A bill for an act. Hawaii State Capitol, Honolulu, HI, USA. [cited 2020 April 30]. Available from: https://www.capitol.hawaii.gov/session2018/bills/SB2571_CD1_.HTM

[etc4948-bib-0079] Stien D , Clergeaud F , Rodrigues AMS , Lebaron K , Pillot R , Romans P , Fagervold S , Lebaron P . 2019. Metabolomics reveal that octocrylene accumulates in *Pocillopora damicornis* tissues as fatty acid conjugates and triggers coral cell mitochondrial dysfunction. Anal Chem 91:990–995. 30516955 10.1021/acs.analchem.8b04187

[etc4948-bib-0080] Stien D , Suzuki M , Rodrigues AMS , Yvin M , Clergeaud F , Thorel E , Lebaron P . 2020. A unique approach to monitor stress in coral exposed to emerging pollutants. Sci Rep 10:1–11. 32541793 10.1038/s41598-020-66117-3 PMC7295770

[etc4948-bib-0081] Sunda W , Kieber DJ , Kiene RP , Huntsman S . 2002. An antioxidant function for DMSP and DMS in marine algae. Nature 418:317–320. 12124622 10.1038/nature00851

[etc4948-bib-0105] Swain TD , Schellinger JL , Strimaitis AM , Reuter KE . 2015. Evolution of anthozoan polyp retraction mechanisms: Convergent functional morphology and evolutionary allometry of the marginal musculature in order Zoanthidea (Cnidaria: Anthozoa: Hexacorallia). BMC Evol Biol 15:123. 26123288 10.1186/s12862-015-0406-1 PMC4486433

[etc4948-bib-0082] Tanaka Y , Miyajima T , Watanabe A , Nadaoka K , Yamamoto T , Ogawa H . 2011. Distribution of dissolved organic carbon and nitrogen in a coral reef. Coral Reefs 30:533–541.

[etc4948-bib-0083] Tashiro Y , Kameda Y . 2013. Concentration of organic sun‐blocking agents in seawater of beaches and coral reefs of Okinawa Island, Japan. Mar Pollut Bull 77:333–340. 24139648 10.1016/j.marpolbul.2013.09.013

[etc4948-bib-0084] Tsui MMP , Chen L , He T , Wang Q , Hu C , Lam JCW , Lam PKS . 2019. Organic ultraviolet (UV) filters in the South China Sea coastal region: Environmental occurrence, toxicological effects and risk assessment. Ecotoxicol Environ Saf 181:26–33. 31154117 10.1016/j.ecoenv.2019.05.075

[etc4948-bib-0085] Tsui MMP , Lam JCW , Ng TY , Ang PO , Murphy MB , Lam PKS . 2017. Occurrence, distribution, and fate of organic UV filters in coral communities. Environ Sci Technol 51:4182–4190. 28351139 10.1021/acs.est.6b05211

[etc4948-bib-0086] Tsui MMP , Leung HW , Kwan BKY , Ng KY , Yamashita N , Taniyasu S , Lam PKS , Murphy MB . 2015. Occurrence, distribution and ecological risk assessment of multiple classes of UV filters in marine sediments in Hong Kong and Japan. J Hazard Mater 292:180–187. 25804793 10.1016/j.jhazmat.2015.03.025

[etc4948-bib-0087] Tsui MMP , Leung HW , Wai TC , Yamashita N , Taniyasu S , Liu W , Lam PKS , Murphy MB . 2014. Occurrence, distribution and ecological risk assessment of multiple classes of UV filters in surface waters from different countries. Water Res 67:55–65. 25261628 10.1016/j.watres.2014.09.013

[etc4948-bib-0088] US Environmental Protection Agency . 1996. OCSPP 850.1350: Mysid chronic toxicity test. EPA‐712‐C‐96‐120. Washington, DC.

[etc4948-bib-0089] US Environmental Protection Agency . 2007. Method 1694: Pharmaceuticals and personal care products in water, soil, sediment, and biosolids by HPLC/MS/MS. EPA‐821‐R‐08‐002. Washington, DC.

[etc4948-bib-0091] US Environmental Protection Agency . 2016. OCSPP 850.1300: Daphnid chronic toxicity test. EPA‐712‐C‐16‐005. Washington, DC.

[etc4948-bib-0092] US Environmental Protection Agency . 2020a. *Estimation Programs Interface Suite*™ *for Microsoft*® *Windows*, Ver 4.11. Washington, DC.

[etc4948-bib-0093] US Environmental Protection Agency . 2020b. Chemistry Dashboard: Trolamine salicylate. Washington, DC. [cited 2020 June 3]. Available from: https://comptox.epa.gov/dashboard/dsstoxdb/results?search=Trolamine%20salicylate

[etc4948-bib-0094] US Geological Survey . 2006. Techniques of water‐resources investigations. Book 9: Handbooks for water‐resources investigations. Reston, VA. [cited 2020 April 16]. Available from: https://pubs.usgs.gov/twri/index090905.html

[etc4948-bib-0095] US Virgin Islands . 2019. Bill no. 33‐0043. Legislature of the United States Virgin Islands, Charlotte Amalie, St. Thomas. [cited 2020 April 30]. Available from: https://stthomassource.com/wp-content/uploads/sites/2/2019/05/33-0043-1.pdf

[etc4948-bib-0096] van Dam JW , Negri AP , Uthicke S , Mueller JF . 2011. Chemical pollution on coral reefs: Exposure and ecological effects. In Sanchez‐Bayo F , van den Brink PJ , Mann RM , eds, Ecological Impacts of Toxic Chemicals. Sharjah, United Arab Emirates, Bentham, pp 187–211.

[etc4948-bib-0097] Watanabe T , Utsunomiya Y , Yuyama I . 2007. Long‐term laboratory culture of symbiotic coral juveniles and their use in ecotoxicological study. J Exp Mar Bio Ecol 352:177–186.

[etc4948-bib-0098] Wijgerde T , van Ballegooijen M , Nijland R , van der Loos L , Kwadijk C , Osinga R , Murk A , Slijkerman D . 2020. Adding insult to injury: Effects of chronic oxybenzone exposure and elevated temperature on two reef‐building corals. Sci Total Environ 733:139030. 32446051 10.1016/j.scitotenv.2020.139030

[etc4948-bib-0099] Wood E . 2018. Impacts of sunscreens on corals. International Coral Reef Initiative, Ministry of the Environment and Energy, Stockholm, Sweden. [cited 2020 April 30]. Available from: https://www.icriforum.org/icri-documents/icri-publications-reports-and-posters/impacts-sunscreens-coral-reefs

[etc4948-bib-0100] Woodhead AJ , Hicks CC , Norström AV , Williams GJ , Graham NAJ . 2019. Coral reef ecosystem services in the Anthropocene. Funct Ecol 33:1023–1034.

[etc4948-bib-0102] Xie W‐H , Shiu W‐Y , Mackay D . 1997. A review of the effect of salts on the solubility of organic compounds in seawater. Mar Environ Res 44:429–444.

[etc4948-bib-0103] Yahel G , Sharp JH , Marie D , Häse C , Genin A . 2003. In situ feeding and element removal in the symbiont‐bearing sponge *Theonella swinhoei*: Bulk DOC is the major source of carbon. Limnol Oceanogr 48:141–149.

