# Supplementary material for: A Critical Review of Organic Ultraviolet Filter Exposure, Hazard, and Risk to Corals
Source: Environ Toxicol Chem. 2021 Feb 2;40(4):967–88. doi: 10.1002/etc.4948 (PMC8048829; doi:10.1002/etc.4948)
Supplement: Supplementary file 1 — Supporting information. [file ETC-40-967-s001.pdf]

Supporting Information For:

A CRITICAL REVIEW OF ORGANIC UV FILTER EXPOSURE, HAZARD, AND RISK TO CORALS

Content in Supporting Information

**Text:**	**Page Number**
Text S1: Methods detailing the approach to the review.	S4
Text S2: Data analysis methods including substitution for LODs and LOQs.	S4-S5
Text S3: Toxicity endpoint distributions and methods for converting LOECs to NOECs.	S5-S7
Text S4: Additional details on risk assessment methods.	S7
Text S5: Further information on UV filter fate.	S8
Text S6: Methods detailing approach to sediment data assessment for Table 2	S8-S9
Text S7: Methods detailing approach to coral data assessment for Table 3	S10
Text S8: Additional references	S11

Tables:

Table S1. List of organic UV filters measured in seawater near coral reefs and some of their details and physical and chemical properties.	S12-S13
Table S2. Detailed summary of the twelve studies reporting concentrations of organic UV filters in seawater near or on coral reef locations.	S14-S15
Table S3: Summary of the analytical methods and analyses, including quality control and assurance details used in the twelve studies reporting concentrations of organic UV filters in seawater.	S16-S17
Table S4. Reported limits of detection (LOD), limits of quantitation (LOQ) and extraction recoveries for UV filters in seawater samples.	S18-S19
Table S5**:** Detailed summary of the nine laboratory toxicity studies in intact hard	

and soft corals species exposed to organic UV filters. S20-S25

Table S6: Predicted no-effect concentration (PNEC) derivation for risk quotient (RQ) calculations reported in Table S7. S26-S27

Table S7: The risk quotients (RQs) used in Figure 7 in the main text. S28-S31

Supplementary Text:

Text S1: Methods detailing the approach to the review.

Two approaches were taken to obtain the data. First, combinations of the search terms "organic UV filter", "coral", "marine", "exposure", and "risk" were put into the Google Scholar search engine to identify relevant papers. Second, a search was conducted with the Web of Science database using the same terms and additional ones containing the specific UV filter chemical name (all variations e.g. "BP-3", "oxybenzone", "benzophenone" etc.) with results screened for any papers that contained chemical monitoring data in tropical regions and/or toxicity data in corals. Inorganic UV filters are beyond the scope of this review and subsequently in this text the term 'UV filter' exclusively refers to organic UV filters.

Text S2: Data analysis methods including substitution for LODs and LOQs.

When multiple study sites were reported within a near-reef exposure study, all sites were included (e.g. beach and reef) unless sampling locations were located in regions where coral are not found (e.g. the Arctic; Tsui et al. 2014). If site replicates were reported, they were averaged (e.g. Tsui et al. 2017; Mitchelmore et al. 2019) and when temporal data was reported from the same site, it was considered an independent sample (e.g. Barger et al. 2015). Expanded datasets were obtained when possible from authors who only reported their monitoring data in a table or figure (i.e. Tsui et al. 2014; Bargar et al. 2015). Given the different approaches in reporting limits of detection (LOD) and limits of quantitation (LOQ) (including a lack of reporting) we applied a consistent approach across all studies for handling non-detects.

Left censoring of the environmental data was required when authors reported less than the limit of detection (<LOD). This was achieved by using the equation proposed by Antweiler

(2015). For example, when a sample or replicate fell below the LOD data substitution according to Equation 1 was undertaken (Antweiler 2015).

$$Substitution = \frac{\sqrt{2}}{2} * LOD \qquad [1]$$

Select studies did not report a compound-specific LOD and instead reported a range (Tashiro and Kameda, 2013; Kung et al. 2018). In the case of Tashiro and Kameda (2013) the lower value in the range was used to replace <LOD values for all UV filters in the dataset. This method was chosen because for each of the compounds requiring data replacement, a measured value was reported that was much less that the upper end of the reported LOD range and close to the lower end. In the case of Kung et al. 2018 a more conservative approach could be taken on inspection of the data. The highest LOD reported in the range was used in the left censoring equation. We also evaluated using the lowest LOD in the range and this only changed the data replacement value slightly (i.e. two places after the decimal), therefore it was determined that the use of either LOD would not significantly change the reported results and the highest was used.

If a paper only reported <LOD for a particular compound, no data was reported in Figure 3 and 0% detection frequency given. Data replacement was not used in these instances.

Another issue encountered was when authors reported <LOQ. While the Antweiler (2015) equation is not for this purpose, we applied it in the same manner as the <LOD values for consistency. This LOQ was used in the data replacement equation rather than the LOD. This was required for Schaap and Slijkerman (2018), Mitchelmore et al. (2019) and Downs et al. (2016).

Text S3: Toxicity endpoint distributions and methods for converting LOECs to NOECs.

All reported LOECs were converted to NOECs according to guidance provided in ECHA (2008). This guidance can be found in Table R.10.1. The LOEC can be converted to a NOEC by

taking the next concentration level down (e.g. no significant effect was observed). In the case where the LOEC is observed at the lowest test concentration or a lower test concentration is not reported by the study, the NOEC can be estimated by dividing the LOEC by 2, when the effect is between 10 and 20%. If the effect percentage is unknown a NOEC cannot be calculated. In the vast majority of cases, the treatment concentration below the LOEC was used as the NOEC. We recognize that this is not a perfect solution; however, a NOEC is preferable from a risk assessment perspective than a LOEC. It should also be noted that a dose-response relationship needs to be established to use any of these conversion methods. In certain cases this was not observed, for example several of the endpoints reported by He et al. (2019a,b) reported the LOEC at the highest concentration tested. Therefore, the NOECs reported in Figure 5 (main text) should be treated with caution and seen as preliminary.

Cumulative endpoint ecotoxicity distributions used the nominal exposure values, given that only a few studies attempted to conduct analytical verification and their monitoring was insufficient to understand the mean exposure concentration for each treatment.

The endpoint distributions presented in Figure 5 were derived from the toxicity endpoints summarized in Table S5 that are relevant for risk assessments (i.e. LC/EC50 values from acute tests and NOECs from chronic studies). A total of 75 endpoints were able to be included, while five studies were unable to be included either because effect concentrations were not presented as mass L^{-1} with insufficient data to convert (Danovaro et al., 2008; McCoshum et al., 2016) or lacked a quantitative evaluation of the studied endpoint (Stien et al., 2019; 2020) or did not provide a concentration response test (Wijgerde et al. 2020). The remaining data collected consisted of a variety of statistical endpoints including lowest observed effect concentrations (LOECs), no observed effect concentrations (NOECs), many of which were no observed effect

concentrations at the highest concentration tested (HNOECs), median effect concentration (EC50), and median lethal concentration (LC50). In order to see how these endpoints compare, a small amount of standardization was undertaken. This is because LC(EC)50 and NOEC are common endpoints used in risk assessment, while LOECs are not and should be converted to NOECs as described in detail earlier (ECHA, 2008). Where multiple endpoints were reported those most suitable for risk assessment were used (i.e. LC50s for mortality instead of NOECs or LC20s from Downs et al. 2016). Furthermore, where multiple timepoints were assessed in the same study the most conservative final endpoint was used (i.e. 24 hr not 8 hr for planula mortality in the Downs et al. 2016 study). Endpoints were ordered from lowest effect concentration and the data points number from lowest to highest (e.g. 1 to 75). The list of numbers (equivalent to the number of endpoints in the dataset) were normalized where the lowest number equals zero percent and the highest 100%. This data was then plotted with endpoint concentration on the X-axis and percentile on the Y-axis. This procedure was followed to create endpoint distribution, all analysis and graphs were created using Graphpad Prism software (Graphpad Software, 2017).

Text S4: Additional details on risk assessment methods.

Risk assessments were summarized in a single figure by plotting the risk quotient (RQ) reported for each compound assessed as described (e.g. Tsui et al. 2014). To make the Tsui et al. (2017) risk assessment comparable with the others, risk quotients were calculated based on applying the assessment factor (AF) to the hazard data they selected (Danovaro et al. 2008; Downs et al. 2016) and dividing this by the water column data reported per site in each season.

Text S5: Further information on UV filter fate.

Even amongst freshwater solubility estimates, substantial variability is observed. For example the measured solubility for BP-3 reported in the ECHA database is 6 mg/L (Table 1), whereas values of 68 to 210 mg/L are reported in publications (Table S1). In addition to salinity, the fate of UV filters will also be dependent upon the water chemistry and physical environment (e.g. temperature). Many UV filters photodegrade and the extent is influenced by salinity and dissolved organic matter. For example Li et al. (2016) suggested that deprotonation of BP-3 in saltwater leads to faster photodegradation, whereas in freshwater the interaction of light with organic matter and presence of hydroxyl radicals was more important. Ge et al. (2019) suggested that other components of water chemistry such as nitrate may influence the fate of UV filters. Degradation rates and pathways have been proposed for some UV filters, such as BP-3 (Gong et al. 2019) and EHMC (Vione et al. 2015), but the presence of breakdown products in the environment have yet to be verified.

Text S6: Methods detailing approach to sediment data assessment for Table 2.

The studies all reported data differently and therefore to represent it in Table 2 required slightly different approaches. Mitchelmore et al. (2019) provided the concentrations from each replicate along the site averages (n=3 replicates), but site averages were only reported for frequently detected compounds. Site averages were calculated for less frequently detected compounds (i.e. avobenzone). Data replacement was used according to Equation 1 (main text) when 1 of the three site replicates fell below the LOD, in order to calculate a site average. When two replicates were <LOD, no data substitution was used and the site average was recorded as <LOD. This approach was also taken for the water column data reported in Figures 2 & 3 of the

main text. Multiple LODs are reported for sediment, therefore the lowest LOD was used in the data substitution equation, (e.g. for avobenzone, 0.05 ng/g dw was the LOD used in Eqn. 1). To summarize the data, the median and range was calculated from the means reported for each of the 19 study sites.

The Tsui et al. (2017) data was also reported per site for a total of 7 sites (4 in the wet season and 3 in the dry season). The median, minimum and maximum were calculated based on the means from the 7 sites. No data substitution was required to summarize this dataset.

The Tsui et al (2015) and the Apel et al. (2018) data were presented differently and required a slight adjustment of the approach. In both studies multiple regions were studied and the mean (Apel et al. 2018) or median (Tsui et al. 2015) along with the range were reported based on the number of samples collected in each region. In both cases the median reported in the main text (Table 2) was calculated based on the mean/median reported for each region. For example, the median is based on the region means (Laizhou Bay, Bohai Sea, Yellow sea) reported by Apel et al. (2018). The minimum and maximum was based on the ranges reported for each region, rather than the four means/medians. The number of samples was a summation of the number of samples collected in each region. For example, Tsui et al. (2015) reported 13 samples collected on three separate occasions in Hong Kong and 8 samples collected once in Tokyo Bay for a total of 74 samples. The detection frequency % is based on the total number of samples collected and calculated from the detection frequency presented in each region. No data substitution was required but it should be noted that the median is based on the per region mean/median rather than the results from the full dataset (e.g. 47 samples and 74 samples reported by Tsui et al. 2015 and Apel et al. 2018, respectively) as full datasets were not available unlike for the Mitchelmore et al. (2019) and Tsui et al. (2017) papers.

Text S7: Methods detailing approach to coral data assessment for Table 3.

The coral data was summarized similarly to the sediment data. Mitchelmore et al. (2019) provided data for each replicate and the average of the three replicates as the sample (each site = 3 replicates), for a total of 19 samples. The median, minimum and maximum is based the averages reported per site. For certain UV filters, there were limited detections so the data was not summarized in as an average per site (i.e. EHMC, 4-MBC, AVO, EDP and CN). CN, EHMC and EDP were detected in various replicates; however, the detections were not consistent (only 1 replicate of three) therefore data substitution technique applied in our analysis to calculate a sample average would not be appropriate because more that 60% of the data would need to be replaced (Antweiler 2015). In the case of 4-MBC and AVO, samples averages were calculated from the replicate data provided by the authors. When data substitution was required in order to calculate a sample mean (e.g. one replicate was a <LOD) the lowest LOD for coral reported by Mitchelmore et al. (2019) was used in the data substitution equation (see main text, Equation 1). The LODs used were 4.85 and 1.21 ng/g dry weight for 4-MBC and AVO, respectively.

For the Tsui et al. (2017) data, data for multiple coral species is reported per study site. An average per species per site is provided. For comparability with the Mitchelmore et al. (2019) dataset, a site average was calculated from the species-specific data. This give a total of 7 samples (similar to their sediment dataset). The median, minimum, maximum detection frequency were calculated from this 7 sample set. Data substitution was required in order to calculate site averaged for BP-8, EDP and OC. Similarly to the other datasets, if more than one of the three replicates (e.g. >30%) of the data was <LOD, then the average was <LOD. The LODs used in the data substitution equation were 0.99, 0.22 and 0.12 for BP-8, EDP and OC, respectively.

Text S8: Additional References:

Antweiler RC. 2015. Evaluation of statistical treatments of left-censored environmental data using coincident uncensored data sets: II. Group comparisons. *Environ Sci Technol* 49:13439-13446. doi:10.1021/acs.est.5b02385.

ECHA. 2008. Guidance on information requirements and chemical safety assessment: Chapter R.10: Characterisation of dose [concentration]-response for environment.

Ge J, Huang D, Han Z, Wang X, Wang X, Wang Z. 2019. Photochemical behavior of benzophenone sunscreens induced by nitrate in aquatic environments. *Water Res* 153:178-186. doi:10.1016/j.watres.2019.01.023.

Gong P, Yuan H, Zhai P, Xue Y, Li H, Dong W, Mailhot G. 2015. Investigation on the degradation of benzophenone-3 by UV/H2O2 in aqueous solution. *Chem Eng J* 277: 97–103. doi:10.1016/j.cej.2015.04.078.

Graphpad Software, 2017. Graphpad Prism version 7.00 for Windows. Graphpad Software, La Jolla California USA. www.graphpad.com.

Kameda, Y., Kimura, K., Miyazaki, M., 2011. Occurrence and profiles of organic sunblocking agents in surface water and sediment in Japanese rivers and lakes. Environ. Pollut. 159, 1570–1576.

Li Y, Qiao X, Zhou C, Zhang Y, Fu Z, Chen J. 2016. Photochemical transformation of sunscreen agent benzophenone-3 and its metabolite in surface freshwater and seawater. *Chemosphere* 153:494-499. doi:10.1016/j.chemosphere.2016.03.080.

Supporting Information Tables:

Table S1: Summary list of the organic UV filters measured in seawater near coral reefs (using the abbreviations suggested in Table 1 and in parentheses additional ones used in the literature), their chemical structures and some of the physical and chemical properties reported in the literature or predicted by EPISuite.

**Name of UV Filter: ACD/IUPAC listed first with others reported in papers (common name)**	**Abbreviations**	**CAS Number**	**Molecular Formula**	**Molecular Weight (g/mol)**	**pKa**	**Log K_{ow}**	**Solubility (mg L^{-1})^**	**Structure**
(2,4-Dihydroxyphenyl) (phenyl)methanone *or* 4-dihydroxybenzophenone (Benzoresorcinol or Benzophenone-1)	BP-1	131-56-6	$C_{13}H_{10}O_3$	214.22	7.72	2.96[c,#]<br>3.15[e]	413.4[#]	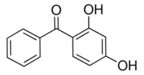
Bis(2,4-dihydroxyphenyl)methanone *or* 2,2',4,4'-tetrahydroxybenzophenone (Benzophenone-2)	BP-2	131-55-5	$C_{13}H_{10}O_5$	246.22	6.98	2.78[#]	398.5[#]	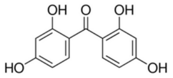
(2-Hydroxy-4-methoxyphenyl)(phenyl)methanone *or* Benzophenone-3 (Oxybenzone)	BP-3	131-57-7	$C_{14}H_{12}0_3$	228.24	7.56	3.52[a,#]<br>3.79[b]<br>3.64[a]	68.56[d,#]<br>210[f]	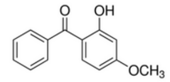
Sulisobenzone *or* 5-Benzoyl-4-hydroxy-2-methoxybenzenesulfonic acid<br><br>(also Benzophenone-4)	BP-4<br><br>(SSB)	4065-45-6	$C_{14}H_{12}O_6S$	308.31	-0.70	0.89[a,#]<br>0.37[a]	2.029[e+004,#]<br>650[f]	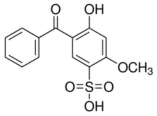
Dioxybenzone *or* (2-Hydroxy-4-methoxyphenyl)(2-hydroxyphenyl)methanone (Benzophenone-8)	BP-8	131-53-3	$C_{14}H_{12}O_4$	244.24	7.11	3.82[c,#]<br>4.31[e]	52.73[#]	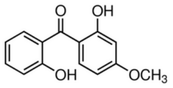
Octocrylene *or* 2-Ethylhexyl 2-cyano-3,3-diphenylacrylate	OC	6197-30-4	$C_{24}H_{27}NO_2$	361.48		6.88[a,#]	0.003808[#]	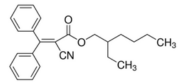
2-Ethylhexyl (2E)-3-(4-methoxyphenyl)acrylate *or* 2-Ethylhexyl 4-methoxycinnamate (Octinoxate)	EHMC<br>(or OMC[i])	5466-77-3	$C_{18}H_{26}O_3$	290.40		5.8[b,#]	0.1548[#]	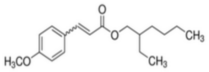
(3E)-1,7,7-Trimethyl-3-(4-methylbenzylidene)bicyclo[2.2.1]heptan-2-one *or* 3-(4-methylbenzylidene) camphor (Enzacamene)	4-MBC	36861-47-9	$C_{18}H_{22}0$	254.37		4.95[a]<br>5.47[b]<br>5.92[#]	0.1966	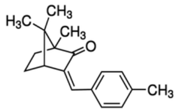

Table S1 continued…

Name of UV Filter:ACD/IUPAC listed first with others reported in papers (common name)	Abbreviations	CAS Number	Molecular Formula	Molecular Weight (g/mol)	pKa	Log K_{ow}	Solubility (mg L^{-1})^	Structure
2-Ethylhexyl 4-(dimethylamino) benzoate<br><br>(Padimate O)	EDP<br>(ODPABA or OD-PABA)	21245-02-3	$C_{17}H_{27}NO_2$	277.40	2.39	6.15[a]<br>5.77[b,#]	0.1977[#]	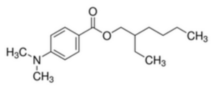
1-(4-Methoxyphenyl)-3-[4-(2-methyl-2-propanyl)phenyl]-1,3-propanedione<br>(Avobenzone)	AVO<br>(BMDMB<br>or BMDM[g,h])	70356-09-1	$C_{20}H_{22}O_3$	310.39	9.74	4.51[a,#]<br>2.41[d]	1.517[#]	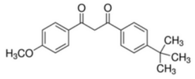
2-Ethylhexyl salicylate<br><br>(Octisalate)	EHS[g,h]<br>(or OS)	118-60-5	$C_{15}H_{22}O_3$	250.33		5.97[b,#]	0.7171[#]	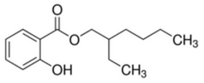
3,3,5-Trimethylcyclohexyl salicylate<br><br>(Homosalate)	HMS	118-56-9	$C_{16}H_{22}O_3$	262.35	8.13	6.16[b,#]	0.4195[#]	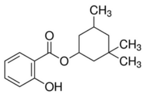
2-Ethoxyethyl (2E)-3-(4-methoxyphenyl)acrylate<br><br>Cinoxate	CIN<br>(CX)	104-28-9	$C_{14}H_{18}O_4$	250.29		2.65[#]	127.4[#]	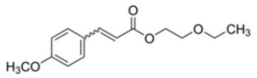
2-Hydroxybenzoic acid - 2,2',2"-nitrilotriethanol (1:1)<br><br>Trolamine salicylate	TEAS	2174-16-5	$C_{13}H_{21}NO_6$	287.31		-	-	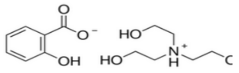
2-Phenyl-1H-benzimidazole-5-sulfonic acid<br>Ensulizole	PSA<br>(or ESZ)	27503-81-7	$C_{13}H_{10}N_2O_3S$	274.30	-0.87	-0.16[#]	2.262[e+004#]	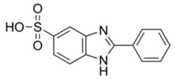
3-Methylbutyl (2E)-3-(4-methoxyphenyl)acrylate<br><br>(Amiloxate)	IPM<br>(or IAMC)	71617-10-2	$C_{15}H_{20}O_3$	248.32		4.33[a,#]	4.858[#]	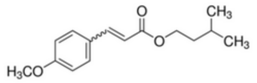
Benzyl salicylate	BZS	118-58-1	$C_{14}H_{12}O_3$	228.24	8.11	4.31[#]	24.59[#]	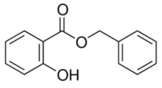

#; As predicted by the US Environmental Protection Agency's EPISuite. ^ Solubility predicted at 25 deg C (mg/L). As listed in [a]: Rodil et al. 2008 [b]; Kameda et al. 2011 [c]: Sanchez-Brunete et al. 2011 [d]: Giokas et al. 2007 [e]: Kim and Choi, 2014 [f]: Diaz-cruz et al. 2008 [g]: Tsui et al. 2019 [h]: Tsui et al. 2014a. [i]: Horricks et al. 2019.

Table S2: Summary of the analytical methods and analyses, including quality control and assurance details used in the twelve studies reporting concentrations of organic UV filters in seawater.

	Bargar et al. 2015	Goksoyr et al 2009 (SML)	Horricks et al. 2019	Kung et al. 2018	Mitchelmore et al. 2019	Tashiro & Kameda 2013	Tsui et al. 2014	Tsui et al. 2019	Tsui et al. 2017	Downs et al. 2016	He et al. 2019a	Schaap & Slijkerman, 2018
**Method Blanks**	N.R.	N.R.	Taken but N.R.[1]	N.R.	Yes	Lab equipment not reported	N.R.	Yes	Yes	N.R.	Same as Tsui 2014	N.R.
**Bottle Blanks**	N.R.	N.R.	Taken but N.R.[1]	N.R.	Yes	N.R.	N.R.	Yes	N.R.	N.R.	Same as Tsui 2014	N.R.
**Transport Blanks**	N.R.	N.R.	Taken but N.R.[1]	N.R.	No	N.R.	N.R.	Unknown	N.R.	N.R.	Same as Tsui 2014	N.R.
**Field equipment Blanks**	N.R.	N.R.	Taken but N.R.[1]	N.R.	Yes	N.R.	N.R.	N.R.	N.R.	N.R.	Same as Tsui 2014	N.R.
**Matrix Spikes**	No[8]	N.R.	N.R.	N.R.[2]	Yes	Yes[3]	Yes	Yes	Yes	None	Same as Tsui 2014[6]	N.R.[9]
**Surrogate Spikes**	N.R.	N.R.	Yes D5-oxybenzone no data reported	N.R.	No	Yes	Yes C13 BP-3	Yes C13 BP-3	Yes C13 BP-3	Yes - but only for GC-MS	None	N.R.
**Check Standards**	N.R.	N.R.	N.R.	N.R.	No	None	N.R.	N.R.	N.R.	N.R.	N.R.	N.R.
**Spike Method Blanks**	Yes	N.R.	N.R.	N.R.	N.R.	N.R.	N.R.	N.R.	Yes	N.R.	N.R.	N.R.
**Reference Material**	N.R.	N.R.	N.R.	N.R.	None	None	None	None	None	None	None	N.R.
**Analytical Replicates**	N.R.	N.R.	Yes	N.R.	Yes	None	Yes	Yes	Yes	N.R.	None for reef	N.R.
**Field Replicates**	None - composites	N.R.	N.R.	Yes	Yes	Some	N.R.	N.R.	Yes	N.R.	None for reef	N.R.
**Internal standards**	1 (p-terphenyl-d14)	1 (benzophenone-d10)	N.R.	N.R.	Yes	None	None - used standard additions	None - used standard additions	None - used standard additions	N.R.	Same as Tsui 2014	N.R.
**SPE material**	Oasis HLB	Sep-Pak Vac C18	None used	None used	Oasis HLB	(DiscoveryDSC-18LT + Discovery DSC-PH)	Bond Elut C18	Bond Elut C18	Bond Elut C18	C18E Phenomenex	Bond Elut C18	ENVI-Chrom-P
**Extraction solvent**	80:20 DCM:methyl–tetra–butyl ether	Methanol and DCM	Methylene Chloride	n-octanol	ACN	DCM	Ethyl acetate:methanol 50:50	Ethyl acetate:methanol 50:50	Ethyl acetate:methanol 50:50	GC-MS ace-ne + DCM, LC-MS/MS methanol	Same as Tsui 2014	Methanol
**Holding time**	<24hrs (SPE frozen)	N.R.	<4hrs then water frozen -80	Unknown	N.R.	N.R.	N.R.	2 weeks[5]	N.R.	Field - N.R.	Same as Tsui 2014	N.R.
**Bottles Used**	Glass	Glass	Plastic	Glass	Glass	N.R.	Glass, Stainless steel, plastic[4]	Glass	N.R.	Glass	Same as Tsui 2014	Glass
**Instrument Used**	GC-MS	GC-MS	LC-MS/MS	LC-MS/MS	LC-MS/MS	GC-MS	LC-MS/MS	LC-MS/MS	LC-MS/MS	GC-MS and LC-MS/MS	LC-MS/MS	LC-MS/MS
**Filtered/ unfiltered**	Filtered	Microlayer filtered	N.R.	Unfiltered	Filtered	Filtered (1 um)	Unfiltered	Unfiltered	Unfiltered	Unfiltered	Unfiltered[7]	N.R.

[1] 2 negative controls, not clear what the matrix was or if 2 samples represent a bottle/transport blank
[2] Standards made in seawater; how the standard was processed is not described and recoveries are reported but it is unclear how these are different from the standards
[3] Spikes made in seawater or "ultra pure" water. The authors do not differentiate which matrix was used with each compound
[4] The authors did not indicate what bottle type was used for any one sample collection, all samples stored in glass

[5] The authors do not provide time of extraction only time of analysis
[6] The authors used spiked toxicology test waters as matrix spikes instead of actual seawater
[7] Environmental samples were unfiltered while experimental samples were filtered.
[8] Spikes made into seawater.
[9] Recoveries are reported but the authors do not say how they were done
SML: surface microlayer samples
N.R.: not reported

Table S3: Reported limits of detection (LOD), limits of quantitation (LOQ) (ng L^{-1}) and extraction recoveries (R, %) for UV filters in seawater samples detailed in the exposure monitoring studies.

**Sampling Location**	**[BP-3]**	**[EHMC]**	**[OC]**	**[HMS]**	**[ODPABA]**	**[4-MBC]**	**OTHER [UV filters]**	**Reference**
USVI, St John	LOD 3.3 - 5<br>LOQ 3.3 - 100<br>R%<10 -100		LOD 0.7 - 5<br>LOQ 3.3 - 25<br>R%11 -85	LOD 0.3 - 1.7<br>LOQ 1.7 - 33<br>R%<10 -100	LOD 0.3 - 5<br>LOQ 1.7 - 25<br>R%<11 -68	LOD 0.3 - 5<br>LOQ 1.7 - 25<br>R%68 -100		Bargar et al. 2015#
USVI, St John<br><br>Hawaii, Oahu and Maui	N.R.<br><br>LOD 100<br>LOQ 5000<br>R% >83							Downs et al. 2016
Japan, Okinawa: (reef and beach sites)	LOD 0.1<br>LOQ N.R.<br>R% 113.2 ± 11.3	LOD 0.1<br>LOQ N.R.<br>R% 92.5 ± 3.5	LOD 0.3<br>LOQ N.R.<br>R% 94.8 ± 11.1	LOD 0.4<br>LOQ N.R.<br>R% 95.6 ± 5.8	LOD 0.2<br>LOQ N.R.<br>R% 97.4 ± 6.5	LOD 0.1<br>LOQ N.R.<br>R% 111.2 ± 4.3	BZS: LOD 3.0<br>R% 106.9 ± 12.3<br>OS: LOD 0.4<br>R% 104.4 ± 2.6	Tashiro & Kameda, 2013^
Hong Kong (reef sites)	LOD 0.04<br>LOQ N.R.<br>R% 93 ± 8	LOD 0.41<br>LOQ N.R.<br>R% 83 ± 4	LOD 1.38<br>LOQ N.R.<br>R% 76 ± 5		LOD 0.03<br>LOQ N.R.<br>R% 73 ± 4	LOD 0.28<br>LOQ N.R.<br>R% 83 ± 4	BP-1: LOD 0.11<br>R% 106 ± 8<br><br>BP-8: LOD 0.03<br>R% 100 ± 6	Tsui et al. 2017
Hong Kong (reef sites only)	LOD 0.03<br>LOQ N.R.<br>R% 93 ± 8	LOD 0.41<br>LOQ N.R.<br>R% 83 ± 4	LOD 1.38<br>LOQ N.R.<br>R% 76 ± 5	LOD 0.11<br>LOQ N.R.<br>R% 65 ± 3	LOD 0.03<br>LOQ N.R.<br>R% 73 ± 4	LOD 0.28<br>LOQ N.R.<br>R% 83 ± 4	BP-1: LOD 0.11<br>R% 106 ± 8<br>BP-8: LOD 0.03<br>R% 100 ± 6	Tsui et al. 2014
Hawaii, Oahu	LOD 0.1<br>LOQ 0.3	LOD 1.5<br>LOQ 5.0	LOD 0.3<br>LOQ 1.0	LOD 6.06<br>LOQ 20	LOD 0.1<br>LOQ 0.3	LOD 1.5<br>LOQ 5.0	BP-8:<br>LOD 1.5 LOQ 5.0<br>CIN:<br>LOD 0.3 LOQ 1.0<br>TEAS:<br>LOD 0.3 LOQ 1.0<br>ESZ:<br>LOD 0.3 LOQ 1.0<br>OS:<br>LOD 6.06 LOQ 20	Mitchelmore et al. 2019
Taiwan, Kenting (beach sites)	LOD 0.01<br>LOQ 0.034<br>% R 86.2 - 109.3					LOD 0.01<br>LOQ 0.036<br>R% 95 - 109.7	BP-1 LOD 0.012<br>LOQ 0.042<br>R% 94.4 - 103.5	Kung et al. 2018

							BP-8 LOD 0.01 - 0.31 % R 96.6 - 103.5	
Pacific Ocean near Polynesia	N.R.	N.R.				N.R.		Goksoyr et al. 2009
Hong Kong: South China Sea	LOD 0.04<br>LOQ N.R.<br>R % 93 ± 8	LOD 0.41<br>LOQ N.R.<br>R % 83 ± 4	LOD 1.38<br>LOQ N.R.<br>R % 76 ± 5	LOD 0.11<br>LOQ N.R.<br>R % 65 ± 3	LOD 0.03<br>LOQ N.R.<br>R % 73 ± 4	LOD 0.28<br>LOQ N.R.<br>R % 83 ± 4	BP-1: LOD 0.11<br>R% 106± 8<br>BP-4: LOD 0.03<br>R% 103 ± 4<br>BP-8: LOD 0.03<br>R% 100 ± 6<br>BMDM:LOD 0.13<br>R% 74 ± 6<br>EHS(OS):LOD 0.1<br>R% 63 ± 1	Tsui et al. 2019[e]
West Indies, Grenada	N.R.	N.R.			N.R.	N.R.		Horricks et al., 2019
Taiwan, Kenting	LOD 0.04<br>LOQ N.R.<br>R% 91 ± 2						BP-1 LOD 0.11 %<br>R% 97 ± 4<br>BP-4 LOD 0.03<br>R% 99 ± 8<br>BP-8 LOD 0.03<br>R% 95 ± 10	He et al., 2019a
Lac Bay, Bonaire	LOD 6.3<br>LOQ 10<br>R% 80		LOD 3.2<br>LOQ 20<br>R% 63			LOD 5.2<br>LOQ 10<br>R% 73		Schaap & Slijkerman, 2018

#Bargar et al. (2015): Detection limits and recoveries varied with time (i.e. samples collected at different times).
^The QA QC for this study is actually from Kameda et al. (2011).
NOTE: Numbers reported as method detection limits (MDL) are treated as LOD.

Table S4: Summary of the twelve studies reporting concentrations of organic UV filters in seawater near or on coral reef locations. Concentrations reported as ranges in parts per trillion (ng L^{-1}). Samples >1000 ng L^{-1} are in bold all others are <1000 ng L^{-1}.

**Sampling Location**	**Sample Date (mth/yr)**	**Sample Depth***	**[BP-3]**	**[EHMC]**	**[OC]**	**[HMS]**	**[ODPABA]**	**[4-MBC]**	**OTHER [UV filters]**	**Total No. sites**	**Replicate samples?**	**Reference (Total number of data points in study)[f]**
USVI, St John	4/13<br>6/13<br>12/13<br>12/13<br>6/14	S<br>S<br>S<br>ML<br>S	<LOD-310<br>18-**4,700**<br><LOD-110<br><LOD<br>27-**6,400**	<LOD-67<br><LOD-33<br><LOD-41<br><LOD-140<br><LOD-67		<LOD-87<br>110-700<br><LOD-86<br><LOD-210<br>27-**1,800**	<LOD-<LOQ<br><LOD<br><LOD<br><LOD<br><LOD	<LOD<br><LOD<br><LOD<br><LOD<br><LOD		18<br>18<br>9<br>9<br>19	No	Bargar et al. 2015# (n=72)
USVI, St John<br><br>Hawaii, Oahu<br><br>Hawaii, Maui	4/07<br><br>5/11<br><br>6/11	S<br><br>S<br><br>S	<LOD-**1,395,000**<br><LOQ-**19,200**[c]<br><LOQ							8<br><br>5<br><br>2	No	Downs et al. 2016 (n=15)
Japan:<br>- Okinawa (Reef sites)<br>- Okinawa[h] (Beach sites)	4-9/11 (4-5 times)<br>4-10/11 (5 times)	S<br><br>S	<LOD-3.8<br><br>*<LOD - **1,258**[g]*	<LOD-2.3<br><br>*<LOD-143[g]*	<LOD-8.1<br><br>*<LOD-79[g]*	<LOD-3.2<br><br>*<LOD-214[g]*	<LOD<br><br>*<LOD-0.8[g]*		OS:<LOD-0.6<br>BZS:<LOD<br>*OS:<LOD-10[g]*<br>*BZS:<LOD[g]*	2<br><br>4	No<br><br>Some; n=1-3	Tashiro & Kameda, 2013 *(n=31)[g]*
Hong Kong (reef sites)	4/15<br><br>8/15	D<br><br>D	13.7 - 31.9<br><br>12.9-26.1	<LOD<br><br><LOD	10.7-14.2<br><br>8.7-13.2		<LOD<br><br>13.2-22.7	<LOD<br><br><LOD	BP-1:<LOD<br>BP-8:<LOD<br>BP-1:<LOD<br>BP-8:<LOD	3<br><br>4	Yes; n=2	Tsui et al. 2017 (n=14)
Hong Kong (reef sites only[d])	8/12<br>2/13<br>6/13	S<br>S<br>S	40-100<br><LOD-38<br>4-**1,189**	37-147<br><LOD-96<br>63-310	56-231<br>46-112<br>86-674	10-72<br><LOD-68<br>56-140	<LOD<br><LOD<br><LOD	<LOD-92<br><LOD<br><LOD	BP-8:21-117<br>BP-8:12-113<br>BP-8:49-83	9[d]<br>9[d]<br>9[d]	No<br>No<br>No	Tsui et al. 2014b# (n=27)
Hawaii, Oahu	10/2017	S	<LOQ-142.7	<LOD-<LOQ	<LOQ-45.5	<LOD-686.6	<LOD-12.4	<LOD-<LOQ	OS:<LOD-121.2<br>BMDBM:<LOD<br>CN:<LOD<br>ENZ:<LOD<br>SSB:<LOD<br>BP-8:<LOD<br>TEAS:<LOD-<LOQ	19	Yes; n=3	Mitchelmore et al. 2019 (n=57)
Taiwan, Kenting NP[h] (beach sites)	7/16	S	*18.8-**1,233**[g]*					*2.40-7.93[g]*	BP-1*:<LOD-2.11[g]*<br>BP-8:*<LOD[g]*	8	Yes; n=3	Kung et al. 2018 *(n=8)[g]*
Pacific Ocean near Polynesia	7/06	ML	5-6	13- 92^$				18-30		2	No	Goksoyr et al. 2009 (n=2)

Hong Kong; South China Sea[e]	8/12; 2/13; /36/13	S	*2.21-36.65*	*3.84-55.65*	*2.15-71.90*	*3.60-41.84*	*1.07-29.16*	*1.44-98.67*	*BP-1:82-135*<br>*BP-4:54-389*<br>*BP-8:64-117*<br>*BMDM:24-721*<br>*IAMC:63-173*<br>*EHS(OS):61-**1,030***	20	No	Tsui et al. 2019[e] *(n=range of 60 samples)*[g]
West Indies, Grenada	9/17; 2/18; 3/18<br>9/17; 2/18; 3/18	S<br>D	<LOD-123<br><LOD-7	<LOD<br><LOD-8			<LOD<br><LOD	<LOD<br><LOD		4<br>3	No	Horricks et al., 2019 (n=7)
Taiwan, Kenting	4/16	S	<LOD-361.44						BP-1:<LOD-50.18<br>BP-4:<LOD-12.72<br>BP-8:<LOD-65.08	3	No	He et al., 2019a (n=3)
Lac Bay, Bonaire[i]	8/16<br><br>11/16	S<br>ML<br>S<br>ML	<LOQ-**1,540**<br><LOQ-900<br><LOQ<br><LOQ-280		<LOQ-810<br><LOQ-**1,950**<br><LOQ-160<br><LOQ-100			<LOQ<br><LOQ<br><LOQ<br><LOQ		3<br>3<br>3<br>3	No	Schaap and Slijkerman, 2018

<LOD: limit of detection (the same as <MOD; method detection limit). <LOQ (the same as <MOQ) is the limit of quantitation. Note LODs/LOQs when reported vary for each UV filter and between each study.

^; combined total of both congeners (E/Z),

$; potential sample contamination,

*; sample depths are surface seawater samples (S), samples at coral depth or deep collections (D), or microlayer samples (ML).

#; additional data on individual sample concentrations provided by the authors.

[c]; only 1 of 5 samples had a measured value, 4 of 5 were <LOQ.

[d]; the author (Dr. Tsui) provided a dataset of all individual sample numbers for all UV filters measured at sites near coral reefs which were 9 sites in total (site numbers 9-17)

[e]; of the n=60 sites, most are not marine samples or near coral reefs but marine coral reef data is indistinguishable using the data in the paper and supplementary file.

[f]: this is the number of individual discrete data points used in this table (and Figure 2).

[g]; italics indicate average values are used for the whole or part of the range, all other ranges are determined from discrete single samples.

[h]; beach sites, unclear how close they are to coral reefs

[i]; 3 locations; Sorbon Beach, Mangrove Forest and Reef channel

Table S5: Detailed summary of the nine laboratory toxicity studies in hard and soft corals exposed to organic UV filters. The toxicity thresholds reported in the publications are presented together with details of additional biological endpoints that do not report a toxicity threshold.

Coral Species and Life Stage	UV filter	Nominal exposure, µg L^{-1} (solvent and its concentration)	Sampling Interval	Exposure type: time and solution renewal details (if appropriate)	Replicates (# corals/ replicate)	Endpoint(s) Investigated/ Toxicity Thresholds reported[$]	Toxicity Thresholds[$] (µg L^{-1}) Nominal or (measured, if applicable)	Study Issues/ Caveats - notes	Reference
S. pistillata- larvae	BP-3	2.28-228,000 (DMSO, 5 µl L^{-1})	Not done	Acute: 24 hr (light & dark) Static: 8hrs	n=4 (?)	Mortality; LC50	139 (light)[a]<br>799 (dark)[b]	Endpoint reporting inconsistent[abc], DMSO solvent used	Downs et al. 2016[c]
S. pistillata- larvae	BP-3	2.28-228,000 (DMSO, 5 µl L^{-1})	Not done	Acute: 24 hr (light & dark) Static: 8hrs	n=4 (?)	Deformity; EC50	49 (light)<br>137 (dark)	" "	Downs et al. 2016
S. pistillata- larvae	BP-3	2.28-228,000 (DMSO, 5 µl L^{-1})	Not done	Acute: 8 hr (light & dark) Static: none	n=4 (?)	Chlorophyll fluorescence; LOEC	2.28 (light)<br>22.8 (dark)[d]	Endpoint reporting inconsistent[d], DMSO solvent used	Downs et al. 2016
S. pistillata- larvae	BP-3	2.28-228,000 (DMSO, 5 µl L^{-1})	Not done	Acute: 8 hr (light & dark) Static: none	n=4 (?)	DNA damage (Abasic lesions); NOEC	22.8 (light) [e]<br>22.8 (dark)	Endpoint reporting inconsistent[e],	Downs et al. 2016
S. pistillata - In vitro cell culture	BP-3	0.57-228,000 (DMSO, 5 µl L^{-1})	Not done	Acute: 4 hr (light) Static: none	n=4	Cell death; LC50	42	DMSO solvent used	Downs et al. 2016
S. pistillata - In vitro cell culture	BP-3	0.57-228,000 (DMSO, 5 µl L^{-1})	Not done	Acute: 4 hr (dark) Static: none	n=4	Cell death; LC50	671	" "	Downs et al. 2016
P. damicornis -In vitro cell culture	BP-3	0.57-228,000 (DMSO, 5 µl L^{-1})	Not done	Acute: 4 hr (light) Static: none	n=4	Cell death; LC50	8	" "	Downs et al. 2016
A. cervicornis - In vitro cell culture	BP-3	0.57-228,000 (DMSO, 5 µl L^{-1})	Not done	Acute: 4 hr (light) Static: none	n=4	Cell death; LC50	9	" "	Downs et al. 2016
M. annularis - In vitro cell culture	BP-3	0.57-228,000 (DMSO, 5 µl L^{-1})	Not done	Acute: 4 hr (light) Static: none	n=4	Cell death; LC50	74	" "	Downs et al. 2016
M. cavernosa -In vitro cell culture	BP-3	0.57-228,000 (DMSO, 5 µl L^{-1})	Not done	Acute: 4 hr (light) Static: none	n=4	Cell death; LC50	52	" "	Downs et al. 2016
P. asteroides- In vitro cell culture	BP-3	0.57-228,000 (DMSO, 5 µl L^{-1})	Not done	Acute: 4 hr (light) Static: none	n=4	Cell death; LC50	340	" "	Downs et al. 2016

P. divaricata -In vitro cell culture	BP-3	0.57-228,000 (DMSO, 5 µl L^{-1})	Not done	Acute: 4 hr (light) Static: none	n=4	Cell death; LC50	36	“ ”	Downs et al. 2016
S. caliendrum-larvae	BP-1<br>BP-3<br>BP-4<br>BP-8	0.1-1,000 (MeOH, 2 µl/10 ml)	Day 0 (stock solutions) & Day 14	Acute?:14 days Static: no renewal	n=3-4 (10)	Mortality; LC50/LOEC	>1,000 / *>1,000*<br>>1,000 / 1,000<br>>1,000 */ >1,000*<br>>1,000 / 500	One initial dose only; used lower light intensity; no exposure solution renewal	^He et al. 2019a
S. caliendrum-larvae	BP-1<br>BP-3<br>BP-4<br>BP-8	0.1-1,000 (MeOH, 2 µl/10 ml)	Day 0 (stock solutions) & Day 14	Acute?:14 days Static: no renewal	n=3-4 (10)	Bleaching; LOEC	500<br>1,000<br>*>1,000*<br>250	“ ”	^He et al. 2019a
S. caliendrum-larvae	BP-1<br>BP-3<br>BP-4<br>BP-8	10-1,000 (MeOH, 2 µl/10 ml)	Day 0 (s-ck solutions) & Day 14	Acute?:14 days Static: no renewal	n=3-4 (10)	Larval settlement; EC50/LOEC	184.1/10<br>*>1000 (as 1000 n.sig.)*<br>*>1000 (as 1000 n.sig.)*<br>530.1/10	“ ”	^He et al. 2019a
S. caliendrum–adult	BP-1<br>BP-3<br>BP-4<br>BP-8	0.1-1,000 (N.R.)	Day 0 & 7	Acute?:7 days Static: no renewal	n=3 (4)	Mortality; LOEC	*1000*<br>*>1,000*<br>*>1,000*<br>100	No exposure renewal; Measurements only on n=6/ exposure	^He et al. 2019a
S. caliendrum–adult	BP-1<br>BP-3<br>BP-4<br>BP-8	0.1-1,000 (N.R.)	Day 0 & 7	Acute?:7 days Static: no renewal	n=3 (4)	Bleaching; LOEC	*1,000*<br>*1,000*<br>*>1,000*<br>100	“ ”	^He et al. 2019a
S. caliendrum–adult	BP-1<br>BP-3<br>BP-4<br>BP-8	0.1-1,000 (N.R.)	Day 0 & 7	Acute?:7 days Static: no renewal	n=3 (4)	Algal density; LOEC	*1,000*<br>*>1,000*<br>*>1,000*<br>100	No exposure renewal; Measurements only on n=3/ exposure	^He et al. 2019a
S. caliendrum–adult	BP-1<br>BP-3<br>BP-4<br>BP-8	0.1-1,000 (N.R.)	Day 0 & 7	Acute?:7 days Static: no renewal	n=3 (4)	Polyp retraction; LOEC	*1,000*<br>10<br>*>1,000*<br>10	No exposure renewal; Measurements only on n=6/ exposure	^He et al. 2019a
P. damicornis-larvae	BP-1<br>BP-3<br>BP-4<br>BP-8	0.1-1,000 (MeOH,2ul/10ml)	Day 0 (s-ck solutions) & 14	Acute?:14 days Static: no renewal	n=3-4 (10)	Mortality; LOEC	*>1,000*<br>*>1,000*<br>*>1,000*<br>*>1,000*	One initial dose only; used lower light intensity; no renewal of exposure solutions	^He et al. 2019a
P. damicornis-larvae	BP-1<br>BP-3<br>BP-4<br>BP-8	0.1-1,000 (MeOH, 2 µl/10 ml)	Day 0 (s-ck solutions) & Day 14	Acute?:14 days Static: no renewal	n=3-4 (10)	Bleaching; LOEC	*>1,000*<br>*>1,000*<br>*>1,000*<br>*>1,000*	“ ”	^He et al. 2019a
P. damicornis-adult	BP-1<br>BP-3<br>BP-4	0.1-1,000 (N.R.)	Day 0 & 7	Acute?:7 days Static: no renewal	n=3 (4)	Mortality; LOEC	1,000<br>*>1,000*<br>*>1,000*	No exposure renewal;	^He et al. 2019a

	BP-8						1,000	Measurements only on n=6/ exposure	
P. damicornis-adult	BP-1<br>BP-3<br>BP-4<br>BP-8	0.1-1,000<br>(N.R.)	Day 0 & 7	Acute?:7 days Static: no renewal	n=3 (4)	Bleaching; LOEC	1,000<br>N.R.<br>N.R.<br>1,000	" "	^He et al. 2019a
P. damicornis-adult	BP-1<br>BP-3<br>BP-4<br>BP-8	0.1-1,000<br>(N.R.)	Day 0 & 7	Acute?:7 days Static: no renewal	n=3 (4)	Algal density; LOEC	*>1,000*<br>*>1,000*<br>*>1,000*<br>1,000	No exposure renewal; Measurements only on n=3/ exposure	^He et al. 2019a
P. damicornis-adult	BP-1<br>BP-3<br>BP-4<br>BP-8	0.1-1,000<br>(N.R.)	Day 0 & 7	Acute?:7 days Static: no renewal	n=3 (4)	Polyp retraction; LOEC	1,000<br>*>1,000*<br>*>1,000*<br>1,000	No exposure renewal; Measurements only on n=6/ exposure	^He et al. 2019a
Acropora sp.-adult	EHMC<br>BP-3<br>4-MBC<br>AVO<br>OC<br>EHS	33 µl L^{-1}*	Not done	N.R.<br>(in situ^^)	n=3	Bleaching	91% at 24 hr<br>86% at 48 h<br>63% at 48 h<br>No bleaching<br>No bleaching<br>No bleaching	Active ingredient concentration N.R.; Exposure conditions may lead - unhealthy water quality which was N.R.	Danovaro et al. 2008[f]
A. pluchra - adult	4-MBC<br>EHMC<br>BP-3	50 µl L^{-1}*	Not done	N.R.<br>(in situ^^)	n=3	Visual Bleaching	95% at 62 h<br>91% at 96 h<br>93 % at 96 h	" "	Danovaro et al. 2008[f]
Xenia spp. (soft coral species) - adult	BP-3<br>#(& others)	0.26 ml L^{-1}<br>(n.a.)	Not done	Acute: 72 h (with 28 days R) (light)	n=140	Growth	Reduction at 28 days at 0.26 ml L^{-1}	Soft coral species used	McCoshum et al. 2016
S. caliendrum – adult	EHMC<br>OC	0.1-1,000<br>(MeOH; 0.01%)	Day 0, 1 & 7	Acute?:7 days Static: no renewal	n=3 (3)	Mortality; LOEC	1,000<br>*>1,000*	No exposure renewal; Measurements only on n=6/ exposure	He et al. 2019b
S. caliendrum – adult	EHMC<br>OC	0.1-1,000<br>(MeOH; 0.01%)	Day 0, 1 & 7	Acute?:7 days Static: no renewal	n=3 (3)	Visual Bleaching; LOEC	1,000<br>*>1,000*	" "	He et al. 2019b
S. caliendrum – adult	EHMC<br>OC	0.1-1,000<br>(MeOH; 0.01%)	Day 0, 1 & 7	Acute?:7 days Static: no renewal	n=3 (3)	Algal density; LOEC	*>1,000*[g]<br>*>1,000*	No exposure renewal; Measurements only on n=3/ exposure	He et al. 2019b
S. caliendrum – adult	EHMC<br>OC	0.1-1,000<br>(MeOH; 0.01%)	Day 0, 1 & 7	Acute?:7 days Static: no renewal	n=3 (3)	Polyp retraction; LOEC	10<br>1,000	No exposure renewal; Measurements only on n=6/ exposure	He et al. 2019b

P. damicornis – adult	EHMC OC	0.1-1,000 (MeOH; 0.01%)	Day 0, 1 & 7	Acute?:7 days Static: no renewal	n=3 (3)	Mortality; LOEC	*>1,000* *>1,000*	“ ”	He et al. 2019b
P. damicornis – adult	EHMC OC	0.1-1,000 (MeOH; 0.01%)	Day 0, 1 & 7	Acute?:7 days Static: no renewal	n=3 (3)	Visual Bleaching; LOEC	*>1,000* *>1,000*	“ ”	He et al. 2019b
P. damicornis – adult	EHMC OC	0.1-1,000 (MeOH; 0.01%)	Day 0, 1 & 7	Acute?:7 days Static: no renewal	n=3 (3)	Algal Density; LOEC	*>1,000* *>1,000*	No exposure renewal; Measurements only on n=3/ exposure	He et al. 2019b
P. damicornis – adult	EHMC OC	0.1-1,000 (MeOH; 0.01%)	Day 0, 1 & 7	Acute?:7 days Static: no renewal	n=3 (3)	Polyp retraction; LOEC	1,000 1,000	No exposure renewal; Measurements only on n=6/ exposure	He et al. 2019b
S. caliendrum – adult	EHMC :OC mix	100:5-1,500:100 (MeOH; 0.01%)	Day 0, 1 & 7	Acute?:7 days Static: no renewal	n=3 (3)	Mortality; LOEC	1,500:100	“ ”	He et al. 2019b
S. caliendrum – adult	EHMC :OC mix	100:5-1,500:100 (MeOH; 0.01%)	Day 0, 1 & 7	Acute?:7 days Static: no renewal	n=3 (3)	Visual bleaching; LOEC	1,500:100	“ ”	He et al. 2019b
S. caliendrum – adult	EHMC :OC mix	100:5-1,500:100 (MeOH; 0.01%)	Day 0, 1 & 7	Acute?:7 days Static: no renewal	n=3 (3)	Algal density; LOEC	1,500:100	No exposure renewal; Measurements only on n=3/ exposure	He et al. 2019b
S. caliendrum – adult	EHMC :OC mix	100:5-1,500:100 (MeOH; 0.01%)	Day 0, 1 & 7	Acute?:7 days Static: no renewal	n=3 (3)	Polyp retraction; LOEC	100:5	No exposure renewal; Measurements only on n=6/ exposure	He et al. 2019b
P. damicornis – adult	EHMC :OC mix	100:5-1,500:100 (MeOH; 0.01%)	Day 0, 1 & 7	Acute?:7 days Static: no renewal	n=3 (3)	Mortality; LOEC	1,500:100	“ ”	He et al. 2019b
P. damicornis – adult	EHMC :OC mix	100:5-1,500:100 (MeOH; 0.01%)	Day 0, 1 & 7	Acute?:7 days Static: no renewal	n=3 (3)	Visual bleaching; LOEC	1,500:100	“ ”	He et al. 2019b
P. damicornis – adult	EHMC :OC mix	100:5-1,500:100 (MeOH; 0.01%)	Day 0, 1 & 7	Acute?:7 days Static: no renewal	n=3 (3)	Algal density; LOEC	1,500:100	No exposure renewal; Measurements only on n=3/ exposure	He et al. 2019b
P. damicornis – adult	EHMC :OC mix	100:5-1,500:100 (MeOH; 0.01%)	Day 0, 1 & 7	Acute?:7 days Static: no renewal	n=3 (3)	Polyp retraction; LOEC	400:30	No exposure renewal; Measurements only on n=6/ exposure	He et al. 2019b

S. pistillata – adult	OC<br>AVO<br>ET	100-5,000<br>(MeOH, 67 µl L^{-1})	2 h after introduction of compound	Chronic: 35 days<br>Static: 7 day renewal	n=3 (3)	Pho-. Efficiency (F_v/F_m); NOEC	1,000 (519)<br>1,000 (87)<br>5,000 (177)	Measured concentrations much lower than nominal	Fel et al. 2019
S. pistillata – adult	DT	10-5,000<br>(MeOH, 67 µl L^{-1})	2 h after introduction of compound	Chronic: 35 days<br>Static: 7 day renewal	n=3 (3)	F_v/F_m; NOEC	5,000 (305)	" "	Fel et al. 2019
S. pistillata – adult	TSDA	10-5,000<br>(n.a.)	2 h after introduction of compound	Chronic: 35 days<br>Static: 7 day renewal	n=3 (3)	F_v/F_m; NOEC	5,000 (5,025)	" "	Fel et al. 2019
S. pistillata – adult	AVO	100-5,000<br>(MeOH, 67 µl L^{-1})	2 h after introduction of compound	Chronic: 35 days<br>Static: 7 day renewal	n=3 (3)	Mortality observed[h]	5,000 (>516)	" "	Fel et al. 2019[h]
P. damicornis – adult	OC	5-1,000<br>(DMSO, 0.25%)	Not done	Chronic: 7 days<br>Static: 24 h renewal	n= N.R. (3)	Polyp retraction effect reported	300	No mortality, bleaching or detrimental impacts reported, DMSO solvent used	Stien et al. 2019
P. damicornis – adult	OC	5-1,000<br>(DMSO, 0.25%)	Not done	Chronic: 7 days<br>Static: 24 h renewal	n= N.R. (3)	Metabolomic change effect reported	50	" "	Stien et al. 2019
S. pistillata - adult	BP-3	1 µg L^{-1}/0.06 µg L^{-1} measured (DMSO, 0.01 mL L^{-1})	Twice weekly (controls every other week)	Chronic: 6 weeks<br>Flow-through: 33% turnover per day, header dosed every 48 h	n = 5 (1)	Survival, Growth rate, Algal density	No effects	DMSO solvent used, measured concentration much lower than nominal, data did not fully support conclusions	Wijgerde et al. 2020
S. pistillata - adult	BP-3	1 µg L^{-1}/0.06 µg L^{-1} measured (DMSO, 0.01 mL L^{-1})	Twice weekly (controls every other week)	Chronic: 6 weeks<br>Flow-through: 33% turnover per day, header dosed every 48 h	n = 5 (1)	PSII yield	4-5% decrease	" "	Wijgerde et al. 2020
S. pistillata - adult	BP-3	1 µg L^{-1}/0.06 µg L^{-1} measured (DMSO, 0.01 mL L^{-1})	Twice weekly (controls every other week)	Chronic: 6 weeks<br>Flow-through: 33% turnover per day, header dosed every 48 h	n = 5 (1)	Microbiome changes	Increase in *Verrucomicrobiaceae*	" "	Wijgerde et al. 2020
A. tenuis - adult	BP-3	1 µg L^{-1}/0.06 µg L^{-1} measured (DMSO, 0.01 mL L^{-1})	Twice weekly (controls every other week)	Chronic: 6 weeks<br>Flow-through: 33% turnover per day, header dosed every 48 h	n = 5 (3)	Survival<br>(i.e. mortality)	No effect	" "	Wijgerde et al. 2020
A. tenuis - adult	BP-3	1 µg L^{-1}/0.06 µg L^{-1} measured (DMSO, 0.01 mL L^{-1})	Twice weekly (controls every other week)	Chronic: 6 weeks<br>Flow-through: 33% turnover per day, header dosed every 48 h	n = 5 (3)	PSII yield	5% decrease	" "	Wijgerde et al. 2020

P. damnicornis - adult	OC<br>BP-3<br>EHS	5 – 1,000 (2,000 for BP-3 only)<br>(DMSO, 0.25%)	Not done	Chronic 7 days<br>Static: 24 h renewal	n = 1 (5)	Metabolomic changes effect reported	50<br>2,000<br>50	All replicate frags in one container per concentration. High DMSO solvent used	Stien et al. 2020
P. damnicornis - adult	OC	5 – 1,000<br>(DMSO, 0.25%)	Not done	Chronic 7 days<br>Static: 24 h renewal	n = 1 (5)	Mitochondrial function change effect reported	50	" "	Stien et al. 2020
P. damnicornis - adult	EHS	5 – 1,000<br>(DMSO, 0.25%)	Not done	Chronic 7 days<br>Static: 24 h renewal	n = 1 (5)	Stress/ inflammatory response induction effects reported	300	" "	Stien et al. 2020
P. damnicornis - adult	HMS	5 – 1,000<br>(DMSO, 0.25%)	Not done	Chronic 7 days<br>Static: 24 h renewal	n = 1 (5)	Polyp Retraction effect reported	1,000	" "	Stien et al. 2020

NOTE: *S. pistillata* is *Stylophora pistillata*; *P. damicornis* is *Pocillopora damicornis*; *S. caliendrum* is *Seriatopora caliendrum* ; *A. cervicornis* is *Acropora cervicornis*; *M. annularis* is *Montastraea annularis*; *M. cavernosa* is *Montipora cavernosa*; *P. asteroides* is *Porites asteroides*; *P. divaricate* is *Porites divaricate*.
LC50 is the lethal concentration that causes death (mortality) in 50% of the test population, EC50 is the effective concentration that causes 50% of the maximum response; NOEC is the no-observed effect concentration, LOEC is the lowest-observed effect concentration. N.R. = not reported.
[$]; toxicity thresholds (i.e. LC/EC50, LOEC or NOECs) are those reported in the publications, those listed in italics are thresholds not implicitly reported but ones we have inferred based on the data presented in the main text or supplementary files, however, it should be noted that these were not reported by the authors or are derived statistically. When multiple toxicity thresholds are reported for the same biological endpoint the one most appropriate for use in a risk assessment is presented. If multiple toxicity thresholds are reported over the time course of an experiment the final timepoint toxicity threshold is reported.
#; exposure was to a volume of sunscreen product/formulation containing multiple active/inactive ingredients
^; this study looked at a number of sublethal endpoints in larvae and adult nubbins (bleaching, settlement failure) many resulting in no effect at the highest concentrations reported,
*; study used volume of active ingredient of unknown concentration.
[a]; this endpoint was also reported as 1.39 in the manuscripts Table 1 but multiple times as 139 in the main text/abstract/supplementary file (Downs et al. 2016).
[b]; this endpoint was also reported as 779 in the abstract but in the text and Table 1 as 799 (Downs et al. 2016).
[c]; toxicity thresholds reported are different depending upon the statistical methods used (i.e. probit versus regression for LC50/EC50; see Table 1). Results are also presented as NOECs although statistical problems for the endpoints are noted. (Downs et al. 2016).
[d]; Figure 2B shows statistical difference between control and 2.28 μg/L concentration but text reports LOEC at 22.8 μg/L.
[e]; Figure 7A shows that the 22.8 μg/L concentration is statistically different from the control but text reports a NOEC at 22.8 μg/L.
[f]; this study also exposed corals to sunscreen products and are not reported in this table, these sunscreen product exposures used *Acropora* sp. and additional coral species (*S. pistillata* and *Millepora complanate*) and reported bleaching, zooxanthellae damage and viral load endpoints.
[g]; reported in Table 1 as 1000 but text and supplementary figures note a lack of significance at this concentration.
[h]: mortality was not specifically measured but inferred as it is stated in the text, no information as to statistical significance is provided.
^^; this study placed coral nubbins in plastic bags with 2 L seawater, sealed the bags, and placed them on the coral reef.
n.sig.=not significant from controls

Table S6. Predicted no-effect concentration (PNEC) derivation for risk quotient (RQ) calculations reported in Table S7. Information is taken from the Supplementary Files from the relevant publications.

Compound	Test organism	Species	Endpoint	Effect Concentration (µg/L)	Assessment factor	PNEC (µg/L)	Reference (effect concentration)	Reference
4-MBC	Hard coral	*Acropora* sp.	Bleaching (LOEC)	1053	1000	1.053	Danovaro et al. (2008)	Tsui et al. (2014)
4-MBC	Hard coral	*A. pulchra*	Bleaching (LOEC)	1596	1000	1.596	Danovaro et al. (2008)	
EHMC	Hard coral	*Acropora* sp.	Bleaching (LOEC)	2000	1000	2	Danovaro et al. (2008)	
EHMC	Hard coral	*A. pulchra*	Bleaching (LOEC)	3030	1000	3.03	Danovaro et al. (2008)	
BP-3	Hard coral	*Acropora* sp.	Bleaching (LOEC)	2376	1000	2.376	Danovaro et al. (2008)	
BP-3	Coral planula	*S. pistillata*	Mortality (LC50)	139	1000	0.139[A]	Downs et al. (2016)	
BP-3	Coral planula	*S. pistillata*	Deformity (EC50)	49	1000	0.049[B]	Downs et al. (2016)	
BP-3	Hard coral	*Acropora* sp.	Bleaching (LOEC)	2376	1000	2.376[C]	Danovaro et al. (2008)	
BP-3	Hard coral	*A. pulchra*	Bleaching (LOEC)	3600	1000	3.6[D]	Danovaro et al. (2008)	
BP-1/BP-8	Coral larvae	*S. caliendrum*	Settlement (LOEC)	N.R. by author	100	N.R. by author	He et al. (2019a)	He et al. (2019a)
BP-1/BP-3/BP-8	Coral larvae	*S. caliendrum*	Bleaching	N.R. by author	100	N.R. by author	He et al. (2019a)	
BP-1/BP-3/BP-8	Coral larvae	*S. caliendrum*	Death	N.R. by author	100	N.R. by author	He et al. (2019a)	
BP-1/ BP-8	Coral larvae	*S. caliendrum*	Settlement (EC50)	N.R. by author	100	N.R. by author	He et al. (2019a)	

BP-1/BP-3/BP-8	Adult hard coral	*S. caliendrum*	Bleaching	N.R. by author	100	N.R. by author	He et al. (2019a)	
BP-1/BP-3/BP-8	Adult hard coral	*S. caliendrum*	Death	N.R. by author	100	N.R. by author	He et al. (2019a)	
BP-1/BP-3/BP-8	Adult hard coral	*S. caliendrum*	Total polyp retraction	N.R. by author	100	N.R. by author	He et al. (2019a)	
BP-1/BP-3/BP-8	Adult hard coral	*S. caliendrum*	Zooxanthellae density	N.R. by author	100	N.R. by author	He et al. (2019a)	
BP-1/BP-8	Adult hard coral	*P. damicornis*	Bleaching	N.R. by author	100	N.R. by author	He et al. (2019a)	
BP-1/BP-8	Adult hard coral	*P. damicornis*	Death	N.R. by author	100	N.R. by author	He et al. (2019a)	
BP-1/BP-8	Adult hard coral	*P. damicornis*	Total polyp retraction	N.R. by author	100	N.R. by author	He et al. (2019a)	
BP-8	Adult hard coral	*P. damicornis*	Zooxanthellae density	N.R. by author	100	N.R. by author	He et al. (2019a)	
EHMC/OC	Adult hard coral	*S. caliendrum*	Bleaching	N.R. by author	100	N.R. by author	He et al. (2019b)	He et al. (2019b)
EHMC/OC	Adult hard coral	*S. caliendrum*	Death	N.R. by author	100	N.R. by author	He et al. (2019b)	
EHMC/OC	Adult hard coral	*S. caliendrum*	Total polyp retraction	N.R. by author	100	N.R. by author	He et al. (2019b)	
EHMC/OC	Adult hard coral	*P. damicornis*	Total polyp retraction	N.R. by author	100	N.R. by author	He et al. (2019b)	

The Tsui et al. (2017) calculated and reported four separate PNECs and these are noted by the A, B, C, and D letter beside the PNEC and the corresponding RQs in Table S7.
N.R.: not reported

Table S7. The risk quotients (RQs) used in Figure 7 in the main text. The RQs were either taken directly from the Supplementary Information of the corresponding publication or in the case of Tsui et al. (2017) re-calculated based - reflect MEC and PNEC rather than coral $MEC_{internal}$ and $PNEC_{internal}$. The same assessment factor was applied to the endpoint and compared with the concentration measured in the water column, rather than internally.

Site	Compound												Note	Reference
	EHMC		BP-3		4-MBC		OC		BP-1		BP-8			
	RQ_{best}	RQ_{worst}	RQ_{best}	RQ_{worst}	RQ_{best}	RQ_{worst}	RQ_{best}	RQ_{worst}	RQ_{best}	RQ_{worst}	RQ_{best}	RQ_{worst}		
Hong Kong		2.02		2.28		0.36							Risk quotient data in reported in Table S6 of the Supplementary Information. The worst-case hazard quotient was used, calculated by dividing the highest measured concentration per location by the lowest PNEC. Authors report <0.01 which needs to be substituted with just 0.01 as monitoring data used is N.R.	Tsui et al. (2014)
Tokyo Bay		0.048		0.04		0.01								
Thailand		0.05		0.05		0.01								
New York		0.07		0.075		0.01								
Los Angeles		0.07		0.25		0.01								
Shantou		0.04		0.079		0.01								
Chaozhou		0.04		0.02		0.01								
Arctic		0.03		0.01		0.01								
Wu Pai (wet season)			0.10[A]										Risk quotients were calculated based on the water column data and the hazard data they used to calculate their PNECs. This needed to be done to make the risk quotients comparable to other studies. This is because to calculate their risk quotients they used the coral tissue MEC and	Tsui et al. (2017)
Sharp Island (wet season)			0.18[A]											
Ung Kong (wet season)			0.19[A]											
Sung Kong (wet season)			0.09[A]											
Wu Pai (dry season)			0.10[A]											
Sharp Island (dry season)			0.23[A]											
Ung Kong (dry season)			0.21[A]											

Wu Pai (wet season)				0.28[B]									compared this to a PNEC$_{internal}$. PNEC$_{internal}$ was calculated by applying the assessment factor (1000) to the toxicity endpoint sand then multiplying by their highest and lowest field derived BAF. This gave them the best and worst RQ, both of which were reported in the paper. RQs were calculated for all endpoints similarly to how they were presented in the original test. PNEC derivation is reported in Table 1.	
Sharp Island (wet season)				0.50[B]										
Ung Kong (wet season)				0.53[B]										
Sung Kong (wet season)				0.27[B]										
Wu Pai (dry season)				0.28[B]										
Sharp Island (dry season)				0.65[B]										
Ung Kong (dry season)				0.59[B]										
Wu Pai (wet season)			0.006[C]											
Sharp Island (wet season)			0.010[C]											
Ung Kong (wet season)			0.011[C]											
Sung Kong (wet season)			0.006[C]											
Wu Pai (dry season)			0.006[C]											
Sharp Island (dry season)			0.013[C]											
Ung Kong (dry season)			0.012 [C]											
Wu Pai (wet season)			0.0039[D]											
Sharp Island (wet season)			0.0068[D]											
Ung Kong (wet season)			0.0072[D]											
Sung Kong (wet season)			0.0037[D]											

Wu Pai (dry season)			0.0038[D]											
Sharp Island (dry season)			0.0088[D]											
Ung Kong (dry season)			0.0081[D]											
Hong Kong			-	-					0.09	1.35	0.12	1.17	The RQs reported are taken from the He et al. 2019a Supplementary File. Table S10 for Hong Kong and Table S11 for Taiwan. The HQ_{best} and HQ_{worst} is reported for both species (*S. caliendrum* and *P. damicornis*) based on PNECs derived from several endpoints reported in the study. Authors report <0.01 which needs to be substituted with just 0.01 as monitoring data used is N.R.	He et al. (2019a)
Hong Kong			0.01	0.54					0.01	0.03	0.01	0.05		
Hong Kong			0.01	0.54					0.01	0.02	0.01	0.02		
Hong Kong			-	-					0.01	0.07	0.01	0.02		
Hong Kong			0.01	0.54					0.01	0.01	0.01	0.11		
Hong Kong			0.01	0.54					0.01	0.01	0.01	0.11		
Hong Kong			0.01	31.61					0.01	0.01	0.09	0.88		
Hong Kong			0.01	0.54					0.01	0.01	0.01	0.11		
Hong Kong			-	-					0.01	0.01	0.01	0.01		
Hong Kong			-	-					0.01	0.01	0.01	0.01		
Hong Kong			-	-					0.01	0.01	0.01	0.01		
Hong Kong			-	-					-	-	0.01	0.01		
Taiwan			-	-					0.49	0.5	0.6	0.65		
Taiwan			0.01	0.04					0.01	0.01	0.02	0.03		
Taiwan			0.01	0.04					0.01	0.01	0.01	0.01		
Taiwan			-	-					0.03	0.03	0.01	0.01		
Taiwan			0.01	0.04					0.01	0.01	0.06	0.06		
Taiwan			0.01	0.04					0.01	0.01	0.06	0.06		
Taiwan			0.79	2.1					0.01	0.01	0.46	0.49		
Taiwan			0.01	0.04					0.01	0.01	0.06	0.06		
Taiwan			-	-					0.01	0.01	0.01	0.01		
Taiwan			-	-					0.01	0.01	0.01	0.01		
Taiwan			-	-					0.01	0.01	0.01	0.01		
Taiwan			-	-					-	-	0.01	0.01		
Hong Kong	0.01	0.28					-	-					These RQs are those reported in Table S10 of the He et al. 2019b Supplementary Material. The RQ worst and best for EHMC and OC for	He et al. (2019b)
Hong Kong	0.01	0.28					-	-						

Hong Kong	0.22	24.52					0.01	0.54					monitoring sites from Hong Kong and Taiwan. Only the species-specific risk assessment is included (i.e. assessment factor of 100). Authors report <0.01 which needs to be substituted with just 0.01 as monitoring data used is N.R.	
Hong Kong	0.01	0.33					0.01	0.53						
Taiwan	0.05	0.12					-	-						
Taiwan	0.05	0.12					-	-						
Taiwan	4.33	10.48					0.02	0.05						
Taiwan	0.06	0.14					0.02	0.05						
